# Sigma‐1 receptor attenuates osteoclastogenesis by promoting ER‐associated degradation of SERCA2

**DOI:** 10.15252/emmm.202115373

**Published:** 2022-05-25

**Authors:** Xiaoan Wei, Zeyu Zheng, Zhenhua Feng, Lin Zheng, Siyue Tao, Bingjie Zheng, Bao Huang, Xuyang Zhang, Junhui Liu, Yilei Chen, Wentian Zong, Zhi Shan, Shunwu Fan, Jian Chen, Fengdong Zhao

**Affiliations:** ^1^ Department of Orthopaedic Surgery Sir Run Run Shaw Hospital Zhejiang University School of Medicine Hangzhou China; ^2^ Key Laboratory of Musculoskeletal System Degeneration and Regeneration Translational Research of Zhejiang Province Hangzhou China

**Keywords:** ER‐associated degradation, dimemorfan, osteoporosis, SERCA2, Sigma‐1 receptor, Musculoskeletal System

## Abstract

Sigma‐1 receptor (Sigmar1) is a specific chaperone located in the mitochondria‐associated endoplasmic reticulum membrane (MAM) and plays a role in several physiological processes. However, the role of Sigmar1 in bone homeostasis remains unknown. Here, we show that mice lacking Sigmar1 exhibited severe osteoporosis in an ovariectomized model. In contrast, overexpression of Sigmar1 locally alleviated the osteoporosis phenotype. Treatment with Sigmar1 agonists impaired both human and mice osteoclast formation *in vitro*. Mechanistically, SERCA2 was identified to interact with Sigmar1 based on the immunoprecipitation‐mass spectrum (IP‐MS) and co‐immunoprecipitation (co‐IP) assays, and Q615 of SERCA2 was confirmed to be the critical residue for their binding. Furthermore, Sigmar1 promoted SERCA2 degradation through Hrd1/Sel1L‐dependent ER‐associated degradation (ERAD). Ubiquitination of SERCA2 at K460 and K541 was responsible for its proteasomal degradation. Consequently, inhibition of SERCA2 impeded Sigmar1 deficiency enhanced osteoclastogenesis. Moreover, we found that dimemorfan, an FDA‐approved Sigmar1 agonist, effectively rescued bone mass in various established bone‐loss models. In conclusion, Sigmar1 is a negative regulator of osteoclastogenesis, and activation of Sigmar1 by dimemorfan may be a potential treatment for osteoporosis in clinical practice.

## Graphical abstract

Activation of Sigma‐1 receptor by dimemorfan promoted SERCA2 degradation, causing reduction in osteoclast formation. Targeting Sigma‐1 receptor and dimemorfan may be a novel potential therapeutic approach for osteoporosis.

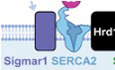

**The paper explained**

**Problem**

Over activation of osteoclasts disturbs the balance between osteoclasts and osteoblasts, leading to bone‐loss diseases. Sigma‐1 receptor has been proved to take part in multiple physiological processes, and its role in regulating bone homeostasis remains unknown.

**Results**

Knockdown of Sigmar1 exacerbated osteoporosis phenotype in OVX mouse model, whereas overexpression of Sigmar1 exerted protective effects. Activation of Sigmar1 by dimemorfan inhibited osteoclast formation *in vitro* and attenuated bone loss in several pathological mouse models. In addition, Sigmar1 was found to interact with SERCA2 and mediated SERCA2 degradation by Hrd1/Sel1L‐dependent ER‐associated degradation.

**Impact**

Sigmar1 acts as a negative regulator in osteoclast formation in pathological conditions, providing new therapeutic target for bone‐loss disease treatment.

## Introduction

The skeleton is of prominent importance in maintaining the normal functions of humans, such as hematopoiesis, hormone excretion, and mechanical support (Garrett & Emerson, 2009; Morrison & Scadden, 2014). The dynamic balance between bone formation and bone resorption is critical to maintain normal bone density and mineral homeostasis (Sobacchi *et al*, 2013), and the process is coupled both in time and space. Osteoclasts are the principal, if not the only cells, to resorb old bone. Excessive osteoclast activity contributes to osteoporosis, Paget’s disease, and rheumatoid arthritis (Singer & Leach, 2010; Walsh & Gravallese, 2010; Compston *et al*, 2019). Current drugs for osteoporosis are mainly aimed at preventing bone resorption; however, in clinical practice, these drugs are still facing many problems and challenges, such as unclear long‐term efficacy, atypical fractures, and increased cardiovascular diseases risks (Khosla & Hofbauer, 2017; Compston *et al*, 2019; Reid, 2020). Therefore, understanding the underlying mechanism that regulates osteoclast differentiation and developing novel drugs for bone‐loss disease treatment are necessary.

Sigma‐1 receptor (Sigmar1) is a nonopioid and evolutionarily isolated receptor with no homolog to any other known human protein (Hanner *et al*, 1996). Crystal analysis revealed that Sigmar1 was a single‐pass transmembrane receptor and formed a trimer structure for ligand binding (Schmidt *et al*, 2016). Normally, Sigmar1 resides specifically at the mitochondria‐associated endoplasmic reticulum (ER) membrane (MAM) and binds with GRP78 (78‐kD glucose‐related protein, also known as Bip) (Hayashi *et al*, 2009). Upon ligand stimulation or ER stress, Sigmar1 dissociates from GRP78 and translocates to the ER lumen to regulate calcium homeostasis and the unfolded protein reaction (UPR) to alleviate ER stress (Hayashi & Su, 2007; Su *et al*, 2016; Rosen *et al*, 2019). Thus, activation of Sigmar1 may be a potential therapeutic target for different diseases. Dimemorfan, an analog of dextromethorphan, is a selective Sigmar1 agonist and a nonopioid antitussive drug that has been safely used in the clinic in Japan for more than 40 years (Ida, 1997; Shen *et al*, 2017). Available studies related to Sigmar1 or dimemorfan mainly focused on neurodegenerative, neuromotor, and respiratory diseases (Luty *et al*, 2010; Al‐Saif *et al*, 2011; Francardo *et al*, 2014; Lenze *et al*, 2020). However, the role of Sigmar1 and the potential application of dimemorfan in bone mineral homeostasis are unknown.

Sacro/endoplasmic reticulum Ca^2+^‐ATPases (SERCAs) are a family of proteins that are involved in calcium homeostasis in multiple cells (Periasamy *et al*, 2017). These proteins are encoded by a multigenic family consisting of SERCA1‐3 (*Atp2a1‐3*). SERCA2b, encoded by *Atp2a2*, is ubiquitously expressed in smooth muscle and nonmuscle tissues, including neurons (Kim *et al*, 2013). By catalyzing adenosine triphosphate (ATP), SERCA2 is the major calcium transport to reuptake calcium ions from the cytoplasm to the endoplasmic reticulum, and this process is essential for intracellular calcium oscillation (Dolmetsch *et al*, 1998; Negishi‐Koga & Takayanagi, 2009). Knocking down SERCA2 remarkably suppressed calcium oscillation at both frequency and peak values, in contrast, overexpression of SERCA2 notably promoted calcium oscillation (Zhao *et al*, 2001; Morita & Kudo, 2010). Heterozygote SERCA2 (+/‐) mice exhibited an osteopetrosis phenotype, and bone marrow‐derived macrophages (BMMs) from these heterozygote mice displayed weak calcium oscillations and less osteoclast formation in the presence of receptor activator of nuclear factor‐κB ligand (RANKL) than those from wild‐type (WT) littermates (Yang *et al*, 2009). A recent study demonstrated that TMEM64 bound to SERCA2 and regulated SERCA2 activity, and knockout of TMEM64 resulted in reduced SERCA2 activity and decreased osteoclast formation (Kim *et al*, 2013).

Here, we investigated the role of Sigmar1 in osteoclastogenesis and found that Sigmar1 global knockout (gKO) mice exhibited severe osteoporosis after ovariectomy surgery (OVX) compared with WT littermates. Activation of Sigmar1 by dimemorfan significantly inhibited osteoclast formation both *in vivo* and *in vitro*. Furthermore, SERCA2 was found to interact with Sigmar1, leading to its proteasomal degradation via the ERAD pathway. The results presented herein demonstrated that Sigmar1 had a profound effect on bone homeostasis and could be a potential therapeutic target for treating osteoporosis.

## Results

### Sigmar1 deletion has no influence on bone mass under steady conditions

**Figure 1:**
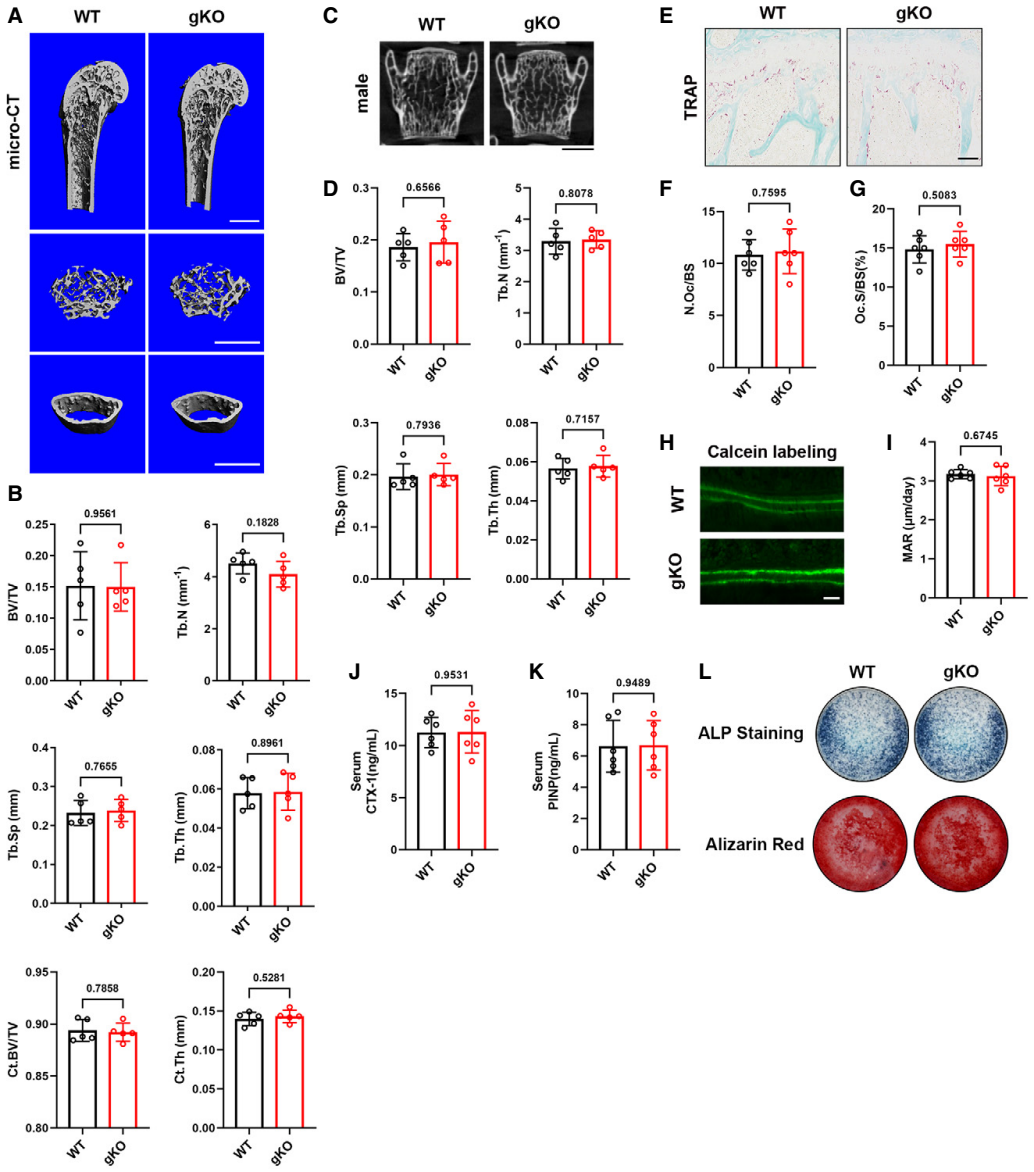
Sigmar1 deletion has no influence on bone mass under steady conditions AMicrocomputed tomography (micro‐CT) images of the proximal femur from 12‐week‐old male WT and Sigmar1 gKO mice. Scale bars, 1 mm.BQuantification of bone volume per tissue volume (BV/TV), trabecular number (Tb. N), trabecular separation (Tb. Sp), trabecular thickness (Tb. Th), cortical region BV/TV (Ct. BV/TV), and cortical thickness (Ct. Th, mm) (*n* = 5 biological replicates).CCoronal images of the fifth lumbar spine. Scale bars, 1 mm.DQuantification of trabecular bone parameters of lumbar spine (*n* = 5 biological replicates).ETRAP staining of tibias from male WT and Sigmar1 gKO mice. Scale bars, 50 μm.F, GQuantification of osteoclast number per bone surface (N. Oc/BS) and percentage of osteoclast surface per bone surface (Oc. S/BS) (*n* = 6 biological replicates).H, IRepresentative images and quantitative analysis of calcein double labeling. Scale bars, 20 μm (*n* = 6 biological replicates).J, KSerum PINP (procollagen I N‐terminal propeptide) and CTX‐I (C‐terminal telopeptide of type I collagen) concentrations measured by ELISA in male Sigmar1 gKO mice and their WT littermates (*n* = 6 biological replicates).LMSCs from male WT or Sigmar1 gKO mice underwent osteogenic differentiation for 7 or 21 days and staining for alkaline phosphatase or alizarin red, respectively. Data information: All results are representative data generated from at least three independent experiments. Data are presented as mean ± SD. The unpaired two‐tailed Student’s *t*‐test (B, D, F, G, I, J, and K) was used for statistical analysis.

To investigate whether loss of Sigmar1 influenced bone mass *in vivo*, we examined 12‐week‐old male Sigmar1 gKO mice and their WT littermates. Compared with wild‐type littermates, Sigmar1 gKO mice displayed normal body size, weight, and fertility. Using micro‐CT, no increase or decrease in trabecular bone mass and cortical bone in the femur and the lumbar spine was observed between the two groups (Fig 1A–D). Similar results were shown in female Sigmar1 gKO mice versus WT littermates (Appendix Fig S1A–D). Tartrate‐resistant acidic phosphatase (TRAP)‐stained sections showed equal osteoclast formation in Sigmar1 gKO versus WT (Fig 1E–G). We performed a calcein labeling experiment and found the same bone formation rate in Sigmar1 gKO versus WT (Fig 1H and I). Immunofluorescence staining of SOST also indicates similar osteocyte number in both genotypes (Appendix Fig S1E and F). Furthermore, the serum procollagen I N‐terminal propeptide (PINP) and C‐terminal telopeptide of type I collagen (CTX‐1), which represent bone formation and bone resorption, respectively, were also unchanged between the two mice in both sexes (Fig 1J and K and Appendix Fig S1G and H). In an *in vitro* bone formation assay, we isolated mesenchymal stem cells (MSCs) from the two gene types and induced them using an osteogenic cell culture medium. ALP and Alizarin Red staining showed similar extracellular calcium deposits between gKO and WT MSCs, indicating that deletion of Sigmar1 had little effect on the osteogenic process (Fig 1L). These results demonstrated that Sigmar1 knockout had no influence on bone mass under steady conditions.

### Loss of Sigmar1 exacerbates OVX‐induced bone loss and promotes osteoclastogenesis *in vitro*

**Figure EV1:**
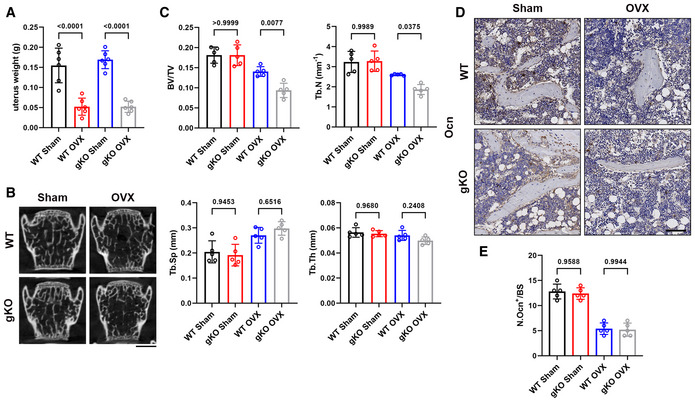
Sigmar1 deletion results in severe osteoporosis in the OVX model and promotes osteoclastogenesis *in vitro* AUterus weight from different groups (*n* = 6 biological replicates).BCoronal images of the fifth lumbar spine. Scale bars, 1 mm.CQuantification of trabecular bone parameters of lumbar spine (*n* = 5 biological replicates).D, EImmunohistochemistry staining of osteocalcin (Ocn) in femur sections (D) and quantification of Ocn‐positive osteoblast on trabecular bone surface (E) (*n* = 5 biological replicates). Data information: All results are representative data generated from at least three independent experiments. Data are presented as mean ± SD. The one‐way ANOVA with the Tukey’s multiple comparison test (A, C, and E) was used for statistical analysis. Source data are available online for this figure.

**Figure 2:**
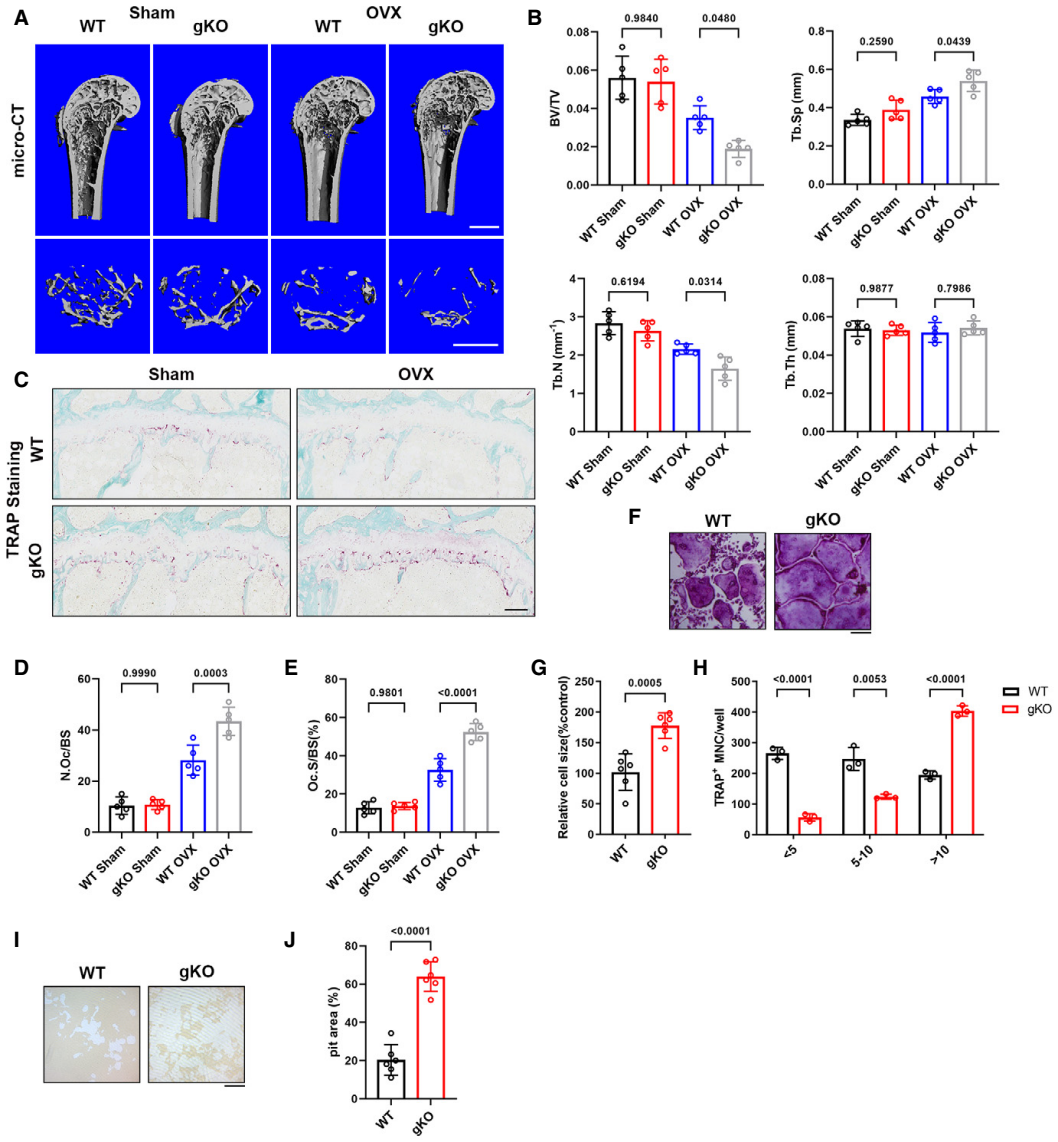
Sigmar1 deletion results in severe osteoporosis in the OVX model and promotes osteoclastogenesis *in vitro* AMicro‐CT images of the proximal femur from female WT or Sigmar1 gKO mice that received sham or ovariectomy surgery for 6 weeks. Scale bars, 1 mm.BQuantification of bone volume per tissue volume (BV/TV), trabecular number (Tb. N), trabecular separation (Tb. Sp), and trabecular thickness (Tb. Th) (*n* = 5 biological replicates).CTRAP staining of femur sections from the four groups. Scale bars, 200 μm.D, EQuantification of osteoclast number per bone surface (N. Oc/BS) and percentage of osteoclast surface per bone surface (Oc. S/BS) (*n* = 5 biological replicates).FTRAP staining to detect osteoclastogenesis of BMMs from WT or Sigmar1 gKO mice. Scale bars, 200 μm.G, HQuantification of the size and nuclei numbers of TRAP‐positive multinuclear cells (*n* = 6 biological replicates for G and *n* = 3 biological replicates for H).I, JRepresentative images and quantification of the relative pit resorption area of hydroxyapatite‐coated plates. WT or Sigmar1 gKO BMMs were seeded on hydroxyapatite‐coated plates and treated with 50 ng/ml RANKL (*n* = 6 biological replicates). Data information: All results are representative data generated from at least three independent experiments. Data are presented as mean ± SD. The one‐way ANOVA with the Tukey’s multiple comparison test (B, D, and E) and unpaired two‐tailed Student’s *t*‐test (G, H, and J) were used for statistical analysis.

To further unveil the role of Sigmar1 in bone homeostasis, we performed ovariectomy surgery (OVX) to investigate events under pathological conditions. Briefly, Sigmar1 gKO and WT littermates were ovariectomized at 12 weeks of age, and 6 weeks later, these mice were harvested for radiographic and histologic analyses. Significant uterus mass loss confirmed the successful establishment of OVX surgery (Fig EV1A). Micro‐CT revealed that Sigmar1 gKO‐OVX mice exhibited a severe osteoporosis phenotype with lower bone volume/tissue volume (BV/TV), trabecular number (Tb. N), and elevated trabecular separation (Tb. Sp) (Fig 2A and B). Radiographic analysis of the lumbar spine also indicated severe bone loss in Sigmar1 gKO mice after OVX surgery (Fig EV1B and C). Next, we performed TRAP staining for osteoclasts and immunohistochemistry (IHC) staining of osteocalcin (Ocn) for osteoblasts in the femur sections and found that Sigmar1 knockout in OVX mice resulted in more osteoclast number, whereas the osteoblast number decreased after OVX surgery and showed no difference between WT and Simgar1 gKO mice (Figs 2C–E and EV1D and E).

**Figure EV2:**
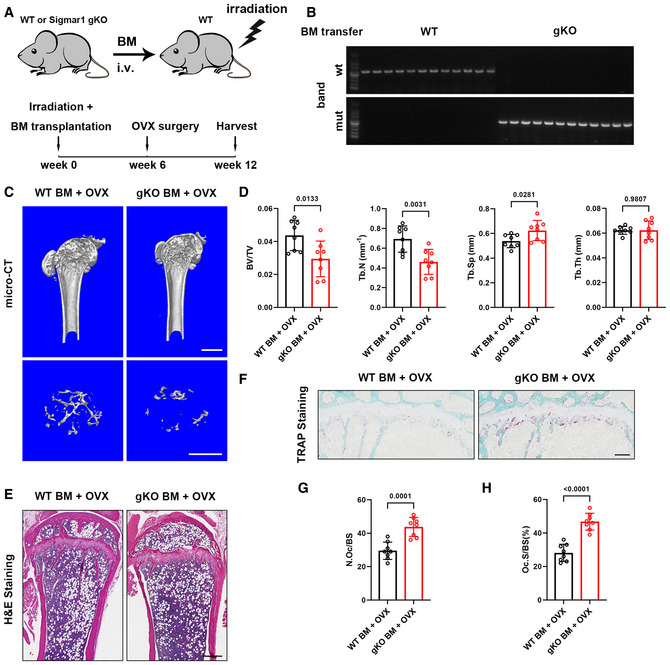
Bone marrow transfer of Sigmar1 gKO cells exacerbates OVX‐induced osteoporosis AThe schematic illustrates the protocol for bone marrow transfer experiment and OVX surgery.BPCR bands for identifying success transfer of WT and Sigmar1 gKO bone marrow cells. Sigmar1 gKO cells had negative wt bands (upper), and positive mut bands (lower) and WT cells had the opposite results.CMicro‐CT images of the proximal femur from female ovariectomized mice that transferred with WT or Sigmar1 gKO bone marrow cells previously. Scale bars, 1 mm.DQuantification of bone volume per tissue volume (BV/TV), trabecular number (Tb. N), trabecular separation (Tb. Sp), and trabecular thickness (Tb. Th) (*n* = 8 biological replicates).EH&E staining of femur sections. Scale bars, 200 μm.FTRAP staining of femur sections. Scale bars, 200 μm.G, HQuantification of osteoclast number per bone surface (N. Oc/BS) and percentage of osteoclast surface per bone surface (Oc. S/BS) (*n* = 8 biological replicates). Data information: All results are representative data generated from at least three independent experiments. Data are presented as mean ± SD. Unpaired two‐tailed Student’s *t*‐test (D and G and H) was used for statistical analysis.

To further investigate the influence of knockout of Sigmar1 on bone mass, we carried out a bone marrow transfer mice model. 6‐week‐old WT mice were subjected to sublethal irradiation and WT or Sigmar1 gKO bone marrow cells were transferred to these mice through tail vein injection (Fig EV2A). 6 weeks after the transfer, we collected mice blood for genotype identification (Fig EV2B), those successfully transferred with WT or Sigmar1 gKO mice were ovariectomized, and all these mice were collected 6 weeks after the OVX surgery for further analysis. Micro‐CT scanning of the femurs showed that mice transferred with Sigmar1 gKO bone marrow exhibited severe osteoporosis after the OVX (Fig EV2C and D). Histological staining also confirmed that Sigmar1 gKO bone marrow transferred mice displayed less trabecular bone and more osteoclast number (Fig EV2E–H).

To elucidate the impact of Sigmar1 on osteoclastogenic cells *in vitro*, we isolated bone marrow‐derived macrophages (BMMs) from both Sigmar1 gKO and WT mice for osteoclast induction. We found that Sigmar1 knockout significantly promoted osteoclast formation (Fig 2F), which showed an increasing number and size of TRAP‐positive cells (Fig 2G and H). Bone resorption analysis indicated that Sigmar1 knockout osteoclast had higher bone resorption capacity (Fig 2I and J). Further RT‐qPCR analysis revealed that marker gene expressions, such as a nuclear factor of activated T cells 1 (*Nfatc1*), *C‐fos*, *Acp5*, cathepsin K (*Ctsk*), *Dc‐stamp* and *Atp6v0d2*, were higher in gKO BMMs than that in WT BMMs (Appendix Fig S2A). These results showed that knockout of Sigmar1 exacerbated bone loss under pathological conditions by promoting osteoclastogenesis.

### Overexpression of Sigmar1 rescues OVX‐induced bone loss

**Figure 3:**
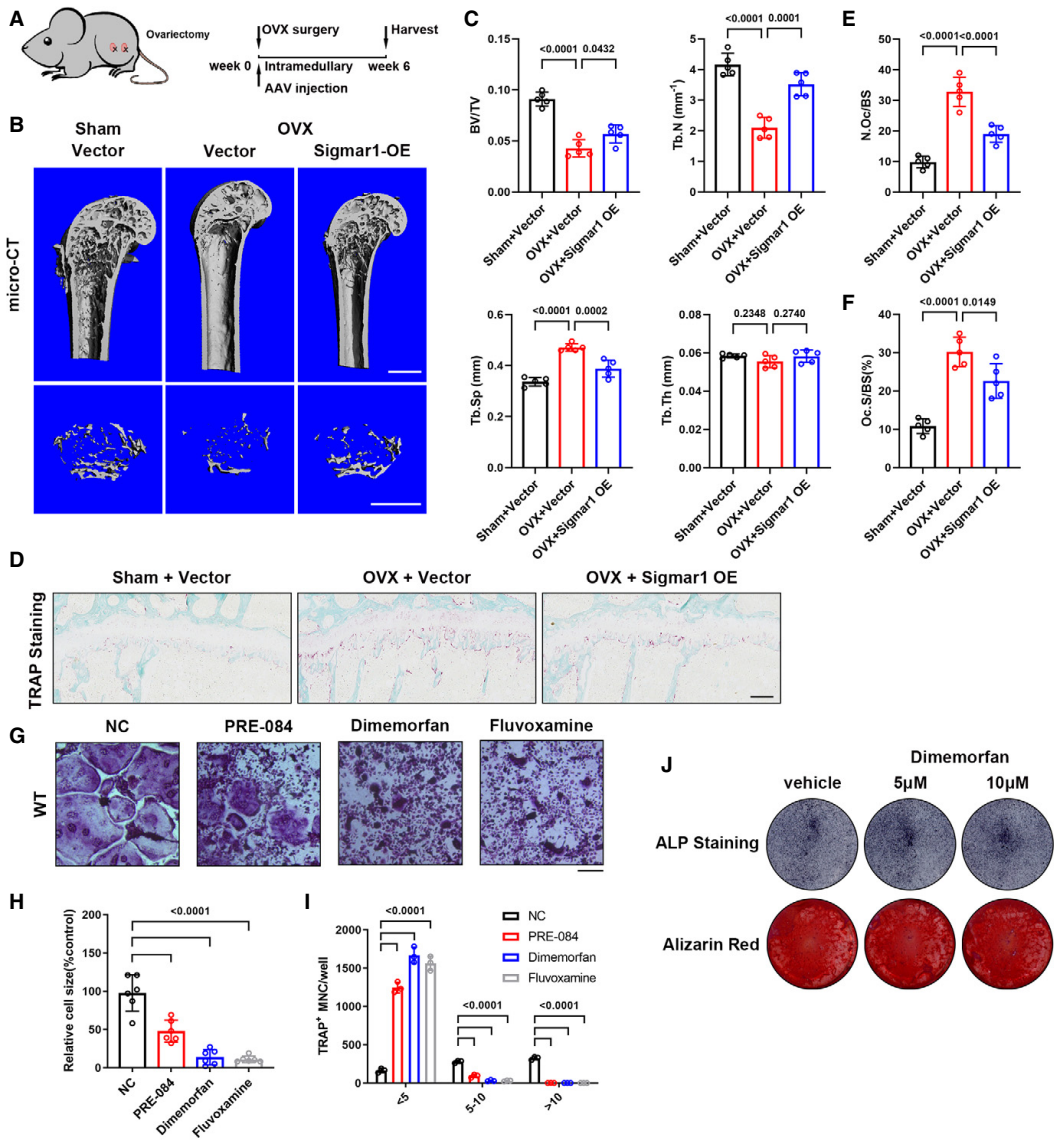
Overexpression of Sigmar1 rescues OVX‐induced bone loss ASchematic illustrating the protocol for OVX‐induced bone loss and AAV‐Sigmar1 treatment. Briefly, 12‐week‐old mice were subjected to either sham or ovariectomy surgery together with different AAV intramedullary injection, and radiological analysis was conducted 6 weeks later.BMicro‐CT images of the proximal femur from sham or OVX mice with different AAV injections. Scale bars, 1 mm.CQuantification of bone volume per tissue volume (BV/TV), trabecular number (Tb. N), trabecular separation (Tb. Sp), and trabecular thickness (Tb. Th) (*n* = 5 biological replicates).DTRAP staining of femur sections from the three groups. Scale bars, 200 μm.E, FQuantification of osteoclast number per bone surface (N. Oc/BS) and percentage of osteoclast surface per bone surface (Oc. S/BS) (*n* = 5 biological replicates).GTRAP staining to detect osteoclastogenesis of BMMs treated with different Sigmar1 agonists (10 μM) or vector. Scale bars, 200 μm.H, IQuantification of the size and nuclei numbers of TRAP‐positive multinuclear cells (*n* = 6 biological replicates for H and *n* = 3 biological replicates for I).JWT MSCs underwent osteogenic differentiation for 7 or 21 days in the presence or absence of dimemorfan and was stained for alkaline phosphatase or alizarin red, respectively. Data information: All results are representative data generated from at least three independent experiments. Data are presented as mean ± SD. The one‐way ANOVA with the Tukey’s multiple comparison test (C, E, F, I, and K–O) was used for statistical analysis.

Next, we conducted an *in vivo* experiment to evaluate the effect of Sigamr1 overexpression on bone loss. Adeno‐associated virus (AAV) was used to overexpress Sigmar1 in osteoclast precursor cells by local injection into bone marrow cavities of the lower limbs. Simultaneously, ovariectomy surgery was also conducted on these mice (Fig 3A). Overexpressing of Sigmar1 in BMMs by AAV injection was further confirmed by western blotting (Appendix Fig S3A). Micro‐CT analysis after six weeks showed that Sigmar1‐overexpressing AAV injection markedly rescued bone loss in OVX mice (Fig 3B and C). TRAP and Ocn staining of the femur sections indicated increased osteoclast number and decreased osteoblast number after the OVX, injection of Sigmar1‐overexpressing AAV caused reduced osteoclast number and had little effect on osteoblast number (Fig 3D–F and Appendix Fig S3B and C).

Then, we speculated whether Sigmar1 agonists could inhibit osteoclastogenesis. Three Sigmar1 agonists (PRE‐084, dimemorfan, and fluvoxamine) were used in this experiment. After 5 days of osteoclast inductions (Fig 3G), we found that all of these agonists could reduce the osteoclast number and size (Fig 3H and I). However, this inhibitory effect was absent when Sigmar1 gKO BMM was treated with these agonists (Appendix Fig S4A–C). Dimemorfan, an FDA‐approved antitussive drug, was further examined in osteoclastogenesis for its potential clinical application value. We treated WT BMMs with different concentrations of dimemorfan and RANKL for 3 days, and the cells were analyzed by RT‐qPCR. Marker genes for osteoclastogenesis were significantly decreased in a dose‐dependent manner compared with the vehicle group (Appendix Fig S4D). Consistently, NFATc1 and c‐fos protein expression together with nuclear translocation of NFATc1 were both impaired after dimemorfan treatment (Appendix Fig S4E and F). Utilizing dimemorfan also inhibited osteoclast resorption capacity, with a reduced pit area observed on the bone plate (Appendix Fig S4G and H). Besides, *in vitro* osteogenic assay of MSCs showed that dimemorfan treatment had no influence on the osteogenic process (Fig 3J). The above results indicated that overexpression or activation of Sigmar1 exerted protective effects on bone loss.

### Sigmar1 interacts with SERCA2 and mediates its degradation

**Figure EV3:**
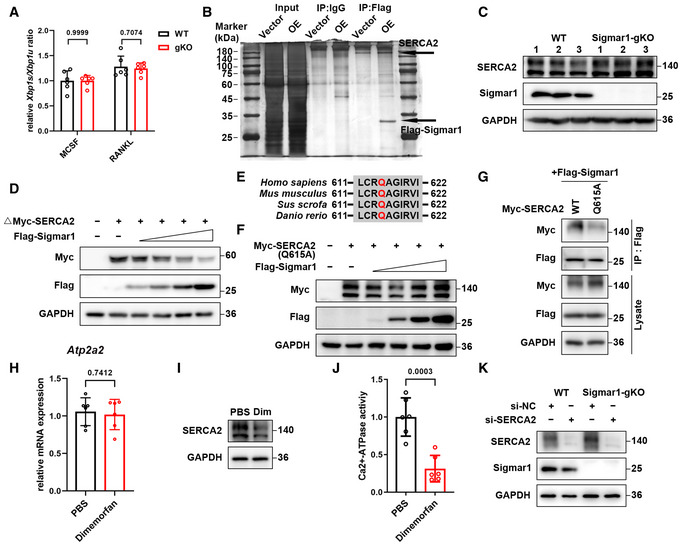
Sigmar1 interacts with SERCA2 and mediates its degradation Relative *xbp1* splicing level of WT or Sigmar1 gKO BMMs during osteoclast formation measured by RT‐qPCR (*n* = 6 biological replicates).Immunoprecipitation of flag‐Sigmar1 interacting protein was visualized by silver gel staining.Western blots showing relative SERCA2 expression in WT and Sigmar1 gKO BMMs.Western blots showing truncated SERCA2 (314–807 aa) expression in HEK‐293T cells transfected with different amounts of Sigmar1 plasmids. The cells were transfected with 1 μg truncated SERCA2 plasmid and Sigmar1 plasmid (0.125, 0.25, 0.5, and 1 μg).Sequence alignment of glutamine residues from SERCA2 orthologs of different species.Western blots showing Q615A mutant full‐length SERCA2 expression in HEK‐293T cells in with different amounts of Sigmar1 plasmids. The cells were transfected with 1 μg Q615A mutant full‐length SERCA2 plasmid and Sigmar1 plasmid (0.125, 0.25, 0.5, and 1 μg).Interactions between Sigmar1 and WT or Q615 mutants of full‐length SERCA2 were detected by Co‐IP assays.Relative mRNA expression of SERCA2 in BMMs treated with PBS or 10 μM dimemorfan for 2 days (*n* = 6 biological replicates).Western blots showing SERCA2 expression in BMMs treated with PBS or 10 μM dimemorfan for 2 days.Relative SERCA2 activity in BMMs treated with PBS or dimemorfan was detected by the Ca2+‐ATPase assay (*n* = 6 biological replicates).Knock down of SERCA2 by siRNA in BMMs was verified by western blotting. Data information: All results are representative data generated from at least three independent experiments. Data are presented as mean ± SD. The unpaired two‐tailed Student’s *t*‐test (A, H and J) was used for statistical analysis. Source data are available online for this figure.

**Figure 4:**
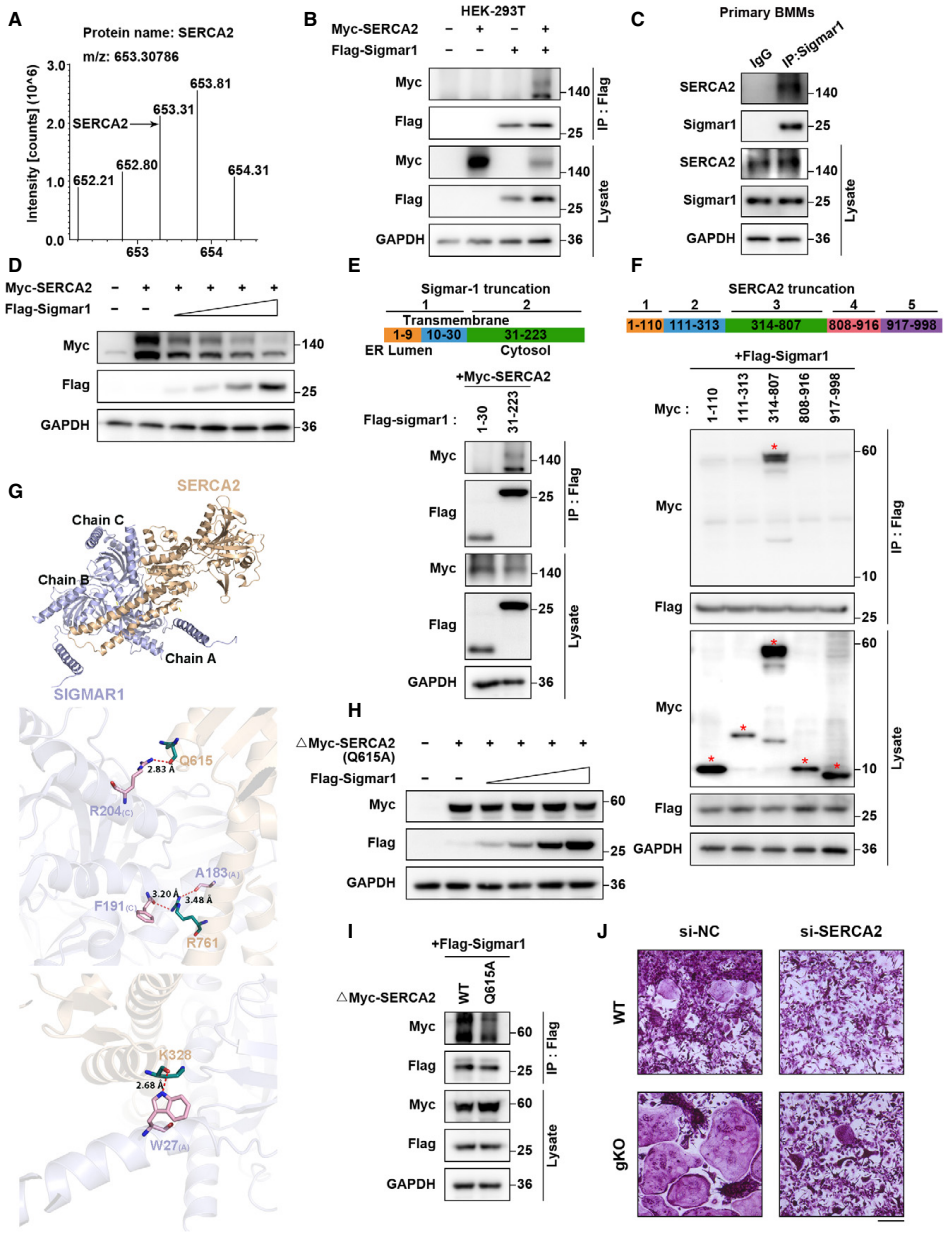
Sigmar1 interacts with SERCA2 and mediates its degradation SERCA2 peptides identified through mass spectrometry are shown.HEK‐293T cells were transfected with the indicated plasmids and then subjected to immunoprecipitation followed by western blotting to detect exogenous interactions between Sigmar1 and SERCA2.The lysates from mouse BMMs were incubated with either anti‐Sigmar1 antibody or normal rabbit IgG, and the pellets were detected with the indicated antibodies.Western blots showing SERCA2 expression in HEK‐293T cells transfected with different amounts of Sigmar1 plasmids. The cells were transfected with 1 μg SERCA2 plasmid and Sigmar1 plasmid (0.125 μg, 0.25 μg, 0.5 μg, and 1 μg).Upper: schematic representation of various Sigmar1 truncations. Bottom: mapping of Sigmar1 domains critical for SERCA2 binding. HEK‐293T cells were transfected with different Sigmar1 truncations, and cell lysates were immunoprecipitated and subjected to western blotting.Upper: schematic representation of various SERCA2 truncations. Bottom: mapping of SERCA2 domains crucial for Sigmar1 binding. Different truncated SERCA2 plasmids were transfected into HEK‐293T cells. After immunoprecipitation, the interaction between truncated SERCA2 and Sigmar1 was detected by western blotting. Red asterisks indicate specific SERCA2 truncation bands.Binding mode of SERCA2 (positions 314–807) on the SIGMAR1 homotrimer predicted by docking. Upper: overall structure of SERCA2 bound to SIGMAR1 in cartoon view. SERCA2 and SIGMAR1 are colored in wheat and light blue, respectively, and the chain identifiers of the SIGMAR1 homotrimer are labeled. Middle and bottom: detailed interaction network between SERCA2 and SIGMAR1. Key residues of SERCA2 (deep teal) and SIGMAR1 (pink) are displayed as sticks, and chain identifiers of residues are shown. H‐bonds are displayed in red dashed lines, and the distances (acceptor to donor heavy atom) of H‐bonds are labeled.Western blots showing Q615A mutant‐truncated SERCA2 expression in HEK‐293T cells transfected with different amounts of Sigmar1 plasmids. The cells were transfected with 1 μg Q615A mutant of truncated SERCA2 plasmid and Sigmar1 plasmid (0.125, 0.25, 0.5, and 1 μg).Interactions between Sigmar1 and WT or Q615 mutants of truncated SERCA2 were detected by Co‐IP assays.TRAP staining to detect osteoclastogenesis of BMMs from WT and gKO mice treated with different siRNAs. Scale bars, 200 μm. Data information: All results are representative data generated from at least three independent experiments. Source data are available online for this figure.

Some studies revealed that Sigmar1 exerted its function through inhibiting IRE1‐xbp1 signaling, and *xbp1* was reported to regulate osteoclastogenesis. Using RT‐qPCR analysis, we found no difference in *xbp1* splicing in the osteoclast formation process in WT and Sigmar1 gKO BMMs (Fig EV3A). To gain insight into the mechanism by which Sigmar1 inhibits osteoclast formation, we screened potential proteins that interacted with Sigmar1 by immunoprecipitation‐mass spectrometry (IP‐MS). SERCA2, an ATPase that allows calcium ions to translocate from the cytosol to the ER lumen, which is an essential process for spiking Ca^2+^ oscillations, was identified as a potential interacting partner (Figs 4A and EV3B). Co‐IP assays showed that both exogenous and endogenous Sigmar1 interacted with SERCA2 in HEK‐293T cells and BMMs, respectively (Fig 4B and C). Interestingly, Western blotting showed that Sigmar1 inhibited SERCA2 expression in a dose‐dependent manner (Fig 4D). In addition, the SERCA2 protein expression level was elevated in Sigmar1 gKO compared with WT mice (Fig EV3C). These results suggested that Sigmar1 interacted with SERCA2 and decreased SERCA2 protein levels.

Then, we sought to identify the specific regions of Sigmar1 and SERCA2 that were critically required for their interaction. We generated two Sigmar1 truncations and five SERCA2 truncations according to their subcellular localization. By co‐IP assays, we found that the C‐terminus of Sigmar1 (31–223 aa) coimmunoprecipitated with SERCA2 (Fig 4E). Correspondingly, the third truncation (314–807 aa) of SERCA2 coimmunoprecipitated with Sigmar1 (Fig 4F). Intriguingly, we found that Sigmar1 also decreased the expression of SERCA2 truncation‐3 in a dose‐dependent manner, which indicated that SERCA2 truncation‐3 was the critical domain for binding to Sigmar1 and leading to SERCA2 degradation (Fig EV3D). To gain further insights into the interaction between Sigmar1 and the SERCA2 truncation (314–807 aa), Sigmar1 was docked with truncated SERCA2 (304–807 aa) (Fig 4G, upper panel). As shown in the middle and lower panels of Fig 4G, three residue pairs between Sigamr1 and truncated SERCA2 were identified as key residues in the formation of hydrogen‐bonding interactions. Taking all of these residue subcellular spatial localizations into consideration, residue R204 of Sigmar1 and residue Q615 of truncated SERCA2, both of which are located in the cytoplasmic region, were selected as putative interaction sites between them. Residue Q615 on SERCA2 was conserved across most mammals (Fig EV3E). To elucidate the role of the Q615 residue on SERCA2, we generated a point mutation in truncated SERCA2 by replacing glutamine with alanine (Q615A). We found that truncated SERCA2 with the Q615A mutation showed resistance to Sigmar1‐induced degradation (Fig 4H). Co‐IP assays also showed a weak interaction between Sigmar1 and the SERCA2 truncation (Q615A), confirming that Q615 on the SERCA2 truncation was essential for Sigmar1 binding with SERCA2 and further regulation of its degradation (Fig 4I). Similar results were obtained when we used full‐length SERCA2 with the Q615A mutation (Fig EV3F and G).

Next, we treated WT BMMs with dimemorfan and found that dimemorfan treatment reduced SERCA2 protein expression but not mRNA expression (Fig EV3H and I), indicating that Sigmar1 inhibited SERCA2 expression through post‐transcriptional regulation. In accordance with downregulated protein expression, SERCA2 activity in BMMs was also reduced following dimemorfan treatment (Fig EV3J). Observing the strong connection between Sigmar1 and SERCA2, we investigated the effect of SERCA2 on Sigmar1‐mediated osteoclastogenesis. Knockdown of SERCA2 by siRNA resulted in reduced osteoclast formation in WT BMMs, and ablation of Sigmar1 deficiency enhanced osteoclastogenesis in gKO BMMs (Figs 4J and EV3K). Altogether, these data suggested that Sigmar1 was conjugated with SERCA2 and promoted its degradation, and SERCA2 played an important role in Sigmar1‐mediated osteoclastogenesis.

### Sigmar1 mediates SERCA2 degradation through the Hrd1/Sel1L‐dependent ERAD pathway

**Figure 5:**
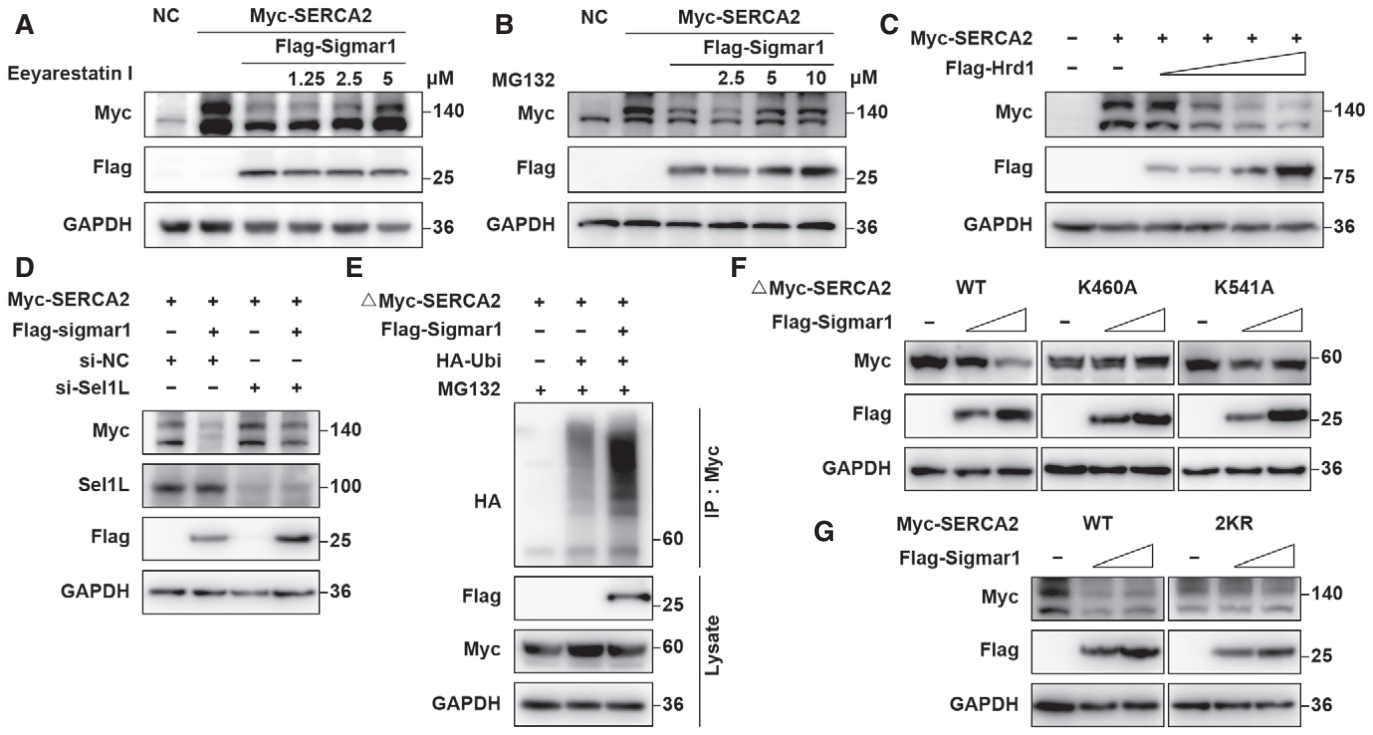
Sigmar1 mediates SERCA2 degradation through the Hrd1/Sel1L‐dependent ERAD pathway A, BWestern blots showing SERCA2 expression in HEK‐293T cells with or without Sigmar1 co‐transfection treated with eeyarestatin I or MG‐132 at the indicated concentration. All inhibitors were applied to cells 8 h prior to protein collection.CWestern blots showing changes in SERCA2 expression in HEK‐293T cells in the presence of different doses of flag‐tagged Hrd1 plasmids.DWestern blots showing alterations of SERCA2 expression in HEK‐293T cells pretreated with negative control siRNA (si‐NC) or Sel1L siRNA in the presence or absence of Sigmar1.ETruncated myc‐tagged SERCA2 ubiquitination levels in HEK‐293T cells transfected with empty vector or Sigmar1 were analyzed by immunoprecipitation. Cells were treated with MG‐132 (10 μM) 8 h before harvest.FExpression of different truncated SERCA2 lysine mutants in HEK‐293T cells transfected with different amounts of Sigmar1 plasmid.GExpression of WT or 2KR (K460A and K541A) mutants of full‐length SERCA2 in HEK‐293T cells transfected with different amounts of Sigmar1 plasmid. Data information: All results are representative data generated from at least three independent experiments. Source data are available online for this figure.

**Figure EV4:**
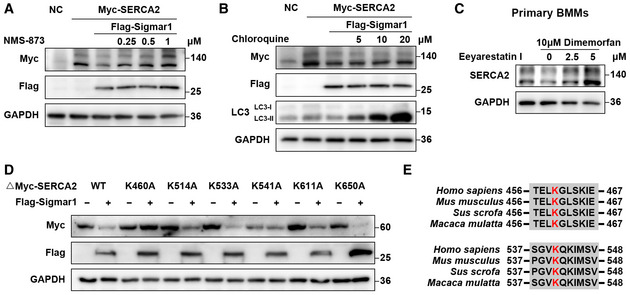
Sigmar1 mediates SERCA2 degradation through the Hrd1/Sel1L‐dependent ERAD pathway A, BWestern blots showing SERCA2 expression in HEK‐293T cells with or without Sigmar1 co‐transfection treated with NMS‐873 or chloroquine at the indicated concentration. All inhibitors were applied to cells 8 h prior to protein collection.CWestern blots showing SERCA2 expression in BMMs treated with dimemorfan or vehicle for 48 h. Eeyarestatin I was added 8 h prior to cell harvest at indicated concentration.DHEK‐293T cells were transfected with different truncated SERCA2 lysine mutants and Sigmar1 and then subjected to western blot analysis.ESequence alignment of Ub sites in SERCA2 orthologs of different species. Data information: All results are representative data generated from at least three independent experiments. Source data are available online for this figure.

As we found that Sigmar1‐mediated SERCA2 degradation, we next investigated the mechanism underlying this process. Because SERCA2 is an ER‐localized protein, we speculated that ERAD might be involved in its degradation. As expected, we found that the ERAD inhibitors eeyarestatin I and NMS‐873 and the proteasome inhibitor MG‐132 effectively rescued the protein level of SERCA2 in a dose‐dependent manner (Figs 5A and B and EV4A), while the lysosome inhibitor chloroquine (CQ) failed to restore SERCA2 protein expression (Fig EV4B). Next, we treated BMMs with dimemorfan and eeyarestatin I. Reduced SERCA2 expression was accompanied by dimemorfan treatment, while eeyarestatin I restored SERCA2 expression (Fig EV4C). These results demonstrated that Sigmar1 and its agonist dimemorfan inhibited SERCA2 expression in both HEK‐293T cells and primary BMMs, and that degradation of SERCA2 was related to the ERAD‐proteasome pathway.

**Figure EV5:**
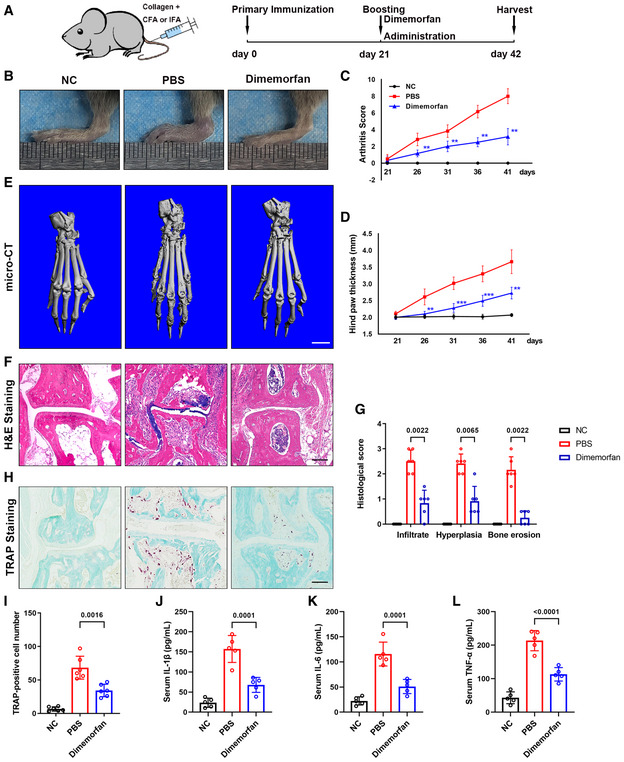
Dimemorfan alleviates joint destruction and osteoclast activity in CIA mice AThe schematic illustrates the protocol for CIA induction and dimemorfan treatment.BPhotographs of representative gross lesions in the hind limbs of CIA mice for clinical assessment.C, DArthritis in PBS‐treated and dimemorfan‐treated mice was induced by chicken type II collagen injection (*n* = 6 mice per group and per time point). After the second immunization, the arthritis score (C) and hind paw thickness (D) were evaluated every 5 days.EMicro‐CT images of paws from CIA mice with different treatments. Scale bars, 2 mm.FH&E staining of ankle joints from the three groups. Scale bars, 50 μm.GCell infiltrate (left), synovial hyperplasia (middle), and bone/cartilage erosion (right) of sections from different groups were analyzed (*n* = 5 biological replicates).HTRAP staining of ankle joints from the three groups. Scale bars, 50 μm.IThe number of TRAP‐positive cells per field of tissue sections stained with TRAP at 100× magnification was analyzed (*n* = 6 biological replicates).J–LSerum IL‐1β, IL‐6, and TNF‐α concentrations measured by ELISA in three groups (*n* = 5 biological replicates). Data information: All results are representative data generated from at least three independent experiments. Data are presented as mean ± SD. The one‐way ANOVA with the Tukey’s multiple comparison test (D and I–L) and nonparameter test (C and G) were used for statistical analysis. **P < 0.01. ***P < 0.001 versus PBS group.

Since Sigmar1 has been reported to coimmunoprecipitate with Hrd1 (Zhou *et al*, 2020) and Hrd1, which interacts with Sel1L to form a complex, is the most important and conserved E3 ubiquitin ligase in the ERAD process (Smith *et al*, 2011). We suspected the potential participation of the Hrd1/Sel1L complex in Sigmar1‐mediated SERCA2 degradation. By overexpressing Hrd1 and SERCA2 in HEK‐293T cells, we found that SERCA2 expression was decreased in a dose‐dependent manner with Hrd1 (Fig 5C). Using a siRNA specific for Sel1L, an obligatory cofactor for Hrd1, to disrupt the Hrd1/Sel1L complex function, Sigmar1 caused a decrease in SERCA2 expression was restored (Fig 5D). These results indicated that the Hrd1/Sel1L complex played a part in SERCA2 degradation.

To further confirm that Sigmar1‐mediated SERCA2 degradation by ubiquitination, we performed a co‐IP assay and found that Sigmar1 promoted truncated SERCA2 ubiquitination, as expected (Fig 5E). Next, we ran the prediction process for the potential ubiquitination site of the SERCA2 truncation on UbiBrowser (http://ubibrowser.ncpsb.org.cn/ubibrowser/) and PLMD (http://plmd.biocuckoo.org/), and six lysine residues (K460, K514, K533, K541, K611, K650) were considered putative ubiquitination sites. Point mutation plasmids of each lysine residue on truncated SERCA2 were constructed, and co‐overexpressed with Sigmar1, K460A, and K541A of SERCA2 truncation clearly rescued Sigmar1‐induced protein reduction (Figs 5F and EV4D). Across common mammals, the K460 and K541 residues on SERCA2 were conserved (Fig EV4E). Consistently, replacing both lysine residues 460 and 541 with alanine (2KR) in full‐length SERCA2 exhibited the same resistance to Sigmar1‐induced SERCA2 degradation (Fig 5G). These results indicated that SERCA2 could be proteasomal degraded by the Hrd1/Sel1L‐dependent ERAD pathway, and that K460 and K541 were the major ubiquitination sites of SERCA2.

### Dimemorfan protects mice from established bone loss in various pathological models

**Figure 6:**
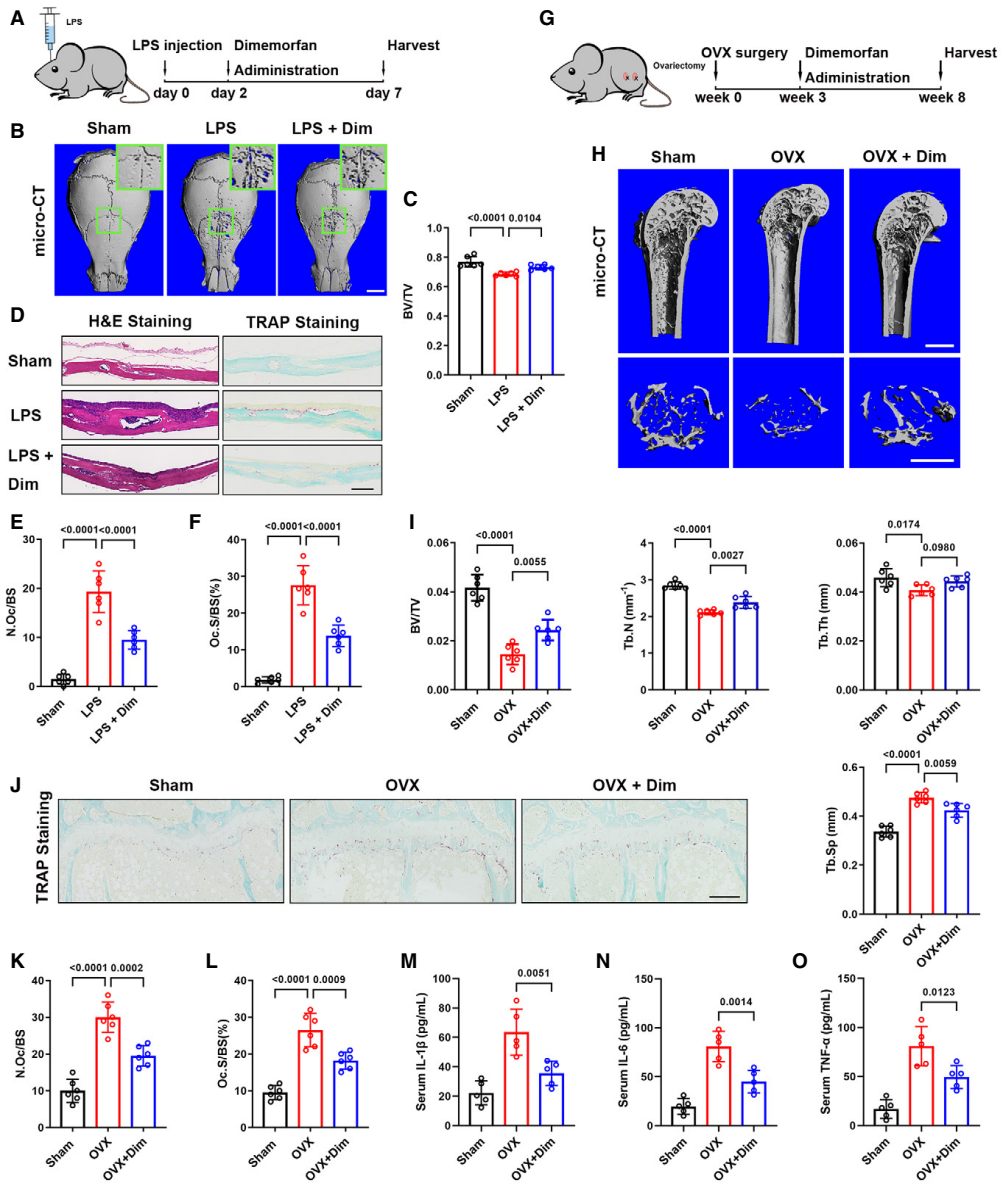
Dimemorfan protects mice from established bone loss in various pathological models ASchematic illustrating the protocol for LPS‐induced osteolysis and dimemorfan treatment.BMicro‐CT images of calvaria from mice that received sham or LPS injection with PBS or dimemorfan treatment. Scale bars, 1 mm.CQuantification of bone volume/tissue volume (BV/TV) of calvaria from different groups (*n* = 6 biological replicates).DH&E and TRAP staining of calvaria from the three groups. Scale bars, 100 μm.E, FQuantification of osteoclast number per bone surface (N. Oc/BS) and percentage of osteoclast surface per bone surface (Oc. S/BS) (*n* = 6 biological replicates).GThe schematic illustrates the protocol for OVX‐induced bone loss and dimemorfan treatment.HMicro‐CT images of proximal femurs from sham or ovariectomized mice with different treatments. Scale bars, 1 mm.IQuantification of bone volume/tissue volume (BV/TV), trabecular number (Tb. N), trabecular separation (Tb. Sp), and trabecular thickness (Tb. Th) (*n* = 6 biological replicates).JTRAP staining of femurs from the three groups. Scale bars, 200 μm.K, LQuantification of osteoclast number per bone surface (N. Oc/BS) and percentage of osteoclast surface per bone surface (Oc. S/BS) (*n* = 6 biological replicates).M–OSerum IL‐1β, IL‐6, and TNF‐α concentrations measured by ELISA in the three groups (*n* = 5 biological replicates). Data information: All results are representative data generated from at least three independent experiments. Data are presented as mean ± SD. The one‐way ANOVA with the Tukey’s multiple comparison test (C, E‐F, I, and K–O) was used for statistical analysis.

To investigate the clinical potential of Sigmar1, we evaluated the therapeutic effect of dimemorfan on various pathological mouse models when the bone loss occurred. In the LPS‐induced acute osteolytic model, WT mice were injected with 25 mg/kg LPS across the calvarial sagittal midline suture under the skull periosteum. Then, dimemorfan was given to mice by intraperitoneal injection 2 days after LPS injection when the skull had been acutely osteolytic (Fig 6A). Craniums were collected 7 days postsurgery, and micro‐CT scanning demonstrated that dimemorfan treatment displayed less porosity and osteolysis on the bone surface with higher BV/TV (Fig 6B and C). H&E staining showed that the vehicle‐treated group had rough surfaces and severe osteolysis compared with the sham group, while in the dimemorfan‐treated group, inflammation was alleviated and showed less inflammatory cell infiltration and bone erosion (Fig 6D, left panel). TRAP staining further confirmed the reduced osteoclast activity in the dimemorfan‐treated group following inflammation stimulation (Fig 6D–F).

Then, we conducted OVX‐induced osteoporosis as a chronic bone‐loss model, and dimemorfan was not given until 3 weeks after OVX surgery to mimic postmenopausal osteoporosis treatment in clinical practice (Fig 6G). After 5 weeks of dimemorfan treatment, all mice were harvested for further analysis. Micro‐CT scanning showed that compared with the sham group, microstructural parameters, such as BV/TV, Tb. N and Tb. Th decreased markedly in the vehicle‐treated group while Tb. Sp increased. OVX mice treated with dimemorfan showed a restoration of osteoporosis by increasing BV/TV, Tb. N and Tb. Th and decreasing Tb. Sp compared with the vehicle‐treated group (Fig 6H and I). Using TRAP staining, the protective effect of dimemorfan was confirmed by reduced osteoclast numbers and areas (Fig 6J–L). ELISA analysis of mice serum showed that IL‐1β, IL‐6, and TNF‐α were elevated after the OVX, and dimemorfan treatment inhibited this phenomenon (Fig 6M–O).

Rheumatic arthritis is always accompanied by elevated osteoclast activity. As a result, collagen‐induced arthritis (CIA) was introduced as an inflammatory, pathological bone erosion model. After booster injection, dimemorfan and PBS were injected intraperitoneally every other day for 3 weeks, and all mice were euthanized 6 weeks after primary immunization (Fig EV5A). Treatment with dimemorfan significantly decreased clinical scores and paw swelling (Fig EV5B–D). Micro‐CT scanning showed less bone erosion on the bone surface in the dimemorfan‐treated group than in the vehicle group (Fig EV5E). Intervention with dimemorfan also reduced the histological score and inflammatory infiltration (Fig EV5F and G). And TRAP staining verified the rescue effect of dimemorfan by decreasing the osteoclast number and distribution (Fig EV5H and I). Further serological experiment showed that CIA caused elevation of IL‐1β, IL‐6, and TNF‐α, treatment of dimemorfan decreased these inflammatory factors (Fig EV5J–L). The above data elucidated that dimemorfan had the potential to alleviate bone loss even if the process had begun, giving it broader clinical application potential.

### Dimemorfan inhibits osteoclast formation in hPBMCs

**Figure 7:**
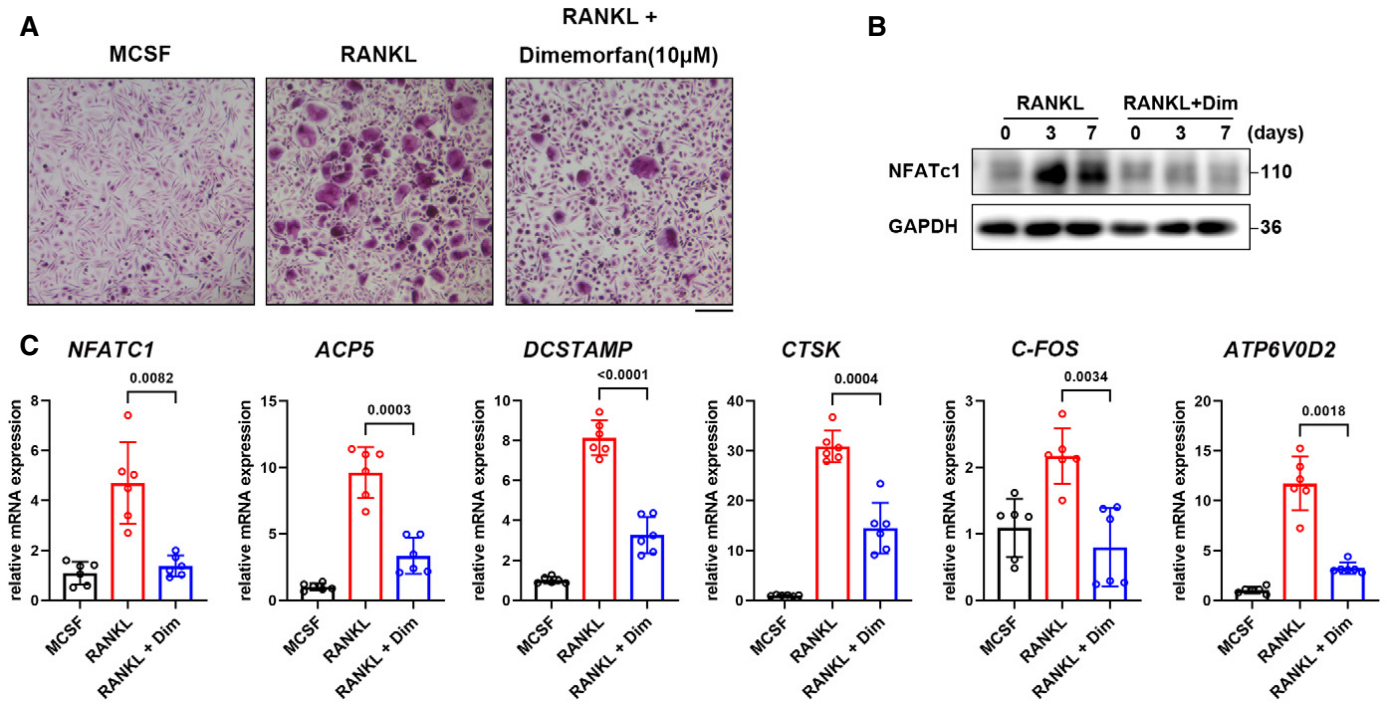
Dimemorfan inhibits osteoclast differentiation in hPBMCs TRAP staining of hPBMCs treated with human RANKL (50 ng/ml) or dimemorfan for 12 days. Scale bars, 500 μm.Western blot analysis of NFATc1 expression during a 7‐day induction of hPBMCs to osteoclasts.hPBMCs were induced to differentiate into osteoclasts for 7 days, and the relative mRNA levels of marker genes were evaluated by RT‐qPCR (*n* = 6 biological replicates). Data information: All results are representative data generated from at least three independent experiments. Data are presented as mean ± SD. The one‐way ANOVA with the Tukey’s multiple comparison test (C) was used for statistical analysis. Source data are available online for this figure.

To further investigate the potential clinical usage of dimemorfan in human for osteoporosis, we examined the effect of dimemorfan on human osteoclastogenic cells. We extracted human peripheral blood mononuclear cells (hPBMCs) from healthy donors and stimulated these cells with human M‐CSF and human RANKL for osteoclast formation. As expected, all cells that received dimemorfan treatment exhibited less osteoclast formation (Fig 7A). Further western blots and RT‐qPCR assays also revealed that proteins and genes related to osteoclastogenesis were downregulated upon dimemorfan treatment (Fig 7B and C).

## Discussion

RANKL‐induced osteoclast formation plays an important role in osteoporosis, and a better understanding of the mechanism underlying osteoclastogenesis can provide multiple choices in treating physiological and pathological bone loss. Sigmar1 has been shown to be involved in multiple pathological conditions, such as amyotrophic lateral sclerosis, Parkinson’s disease, and depression (Al‐Saif *et al*, 2011; Ito *et al*, 2012; Francardo *et al*, 2014). Dimemorfan, a Sigmar1 agonist, is a nonopioid antitussive drug that has been safely used for over 40 years in Japan (Ida, 1997). Dimemorfan has recently been shown to have anti‐amnesia, anti‐convulsant, and anti‐inflammatory effects (Wang *et al*, 2003, 2008; Shin *et al*, 2005). How Sigmar1 and its agonist dimemorfan affect bone homeostasis, however, is not fully understood. Here, we discovered that SERCA2 bound to Sigmar1 and played a role downstream of Sigmar1 in regulating osteoclastogenesis.

In this study, we first unveiled the protective function of Sigmar1 *in vivo*. Under steady conditions, Sigmar1 gKO mice showed a bone mass equivalent to that of WT mice. Further bone section staining and serological tests confirmed that there was no increase or decrease in osteoclast or osteoblast activity *in vivo*, osteocyte number was also unaffected. Under pathological conditions such as OVX‐induced bone loss, however, Sigmar1 gKO mice exhibited severe osteoporosis with a reduced BV/TV and trabecular number, suggesting that Sigmar1 exerted a protective effect on bone homeostasis, especially under pathological conditions. Furthermore, the transfer of Sigmar1 gKO bone marrow cells into WT mice exacerbated the osteoporosis phenotype after the OVX surgery. Next, in the OVX model, we performed an intramedullary injection of AAV to overexpress Sigmar1. As expected, overexpression of Sigmar1 notably rescued OVX‐induced bone loss, further confirming the significant role of Sigmar1 in pathological bone homeostasis.

Normally, Sigmar1 resides in the ER and binds to GRP78, but upon ER stress or changes in calcium levels, Sigmar1 dissociates from GRP78 to exert its functions, e.g., as a molecular chaperone (Hayashi *et al*, 2009). Under pathological conditions, such as ovariectomy surgery, stress increased which allowed more Sigmar1 activation and promoted Sigmar1 to exert protective function, and Sigmar1 deletion under pathological conditions diminished the protection, leading to severe bone loss. However, under normal or unchallenged conditions, stress was much less than in pathological conditions, thus Sigmar1 deletion exhibited an imperceptible effect on bone mass.

In search of the downstream ligand responsible for Sigmar1 regulation in osteoclastogenesis, SERCA2 was identified by immunoprecipitation‐mass spectrometry (IP‐MS). Heterozygous SERCA2 impairs calcium oscillation and leads to defects in osteoclast formation (Yang *et al*, 2009). We found that Sigmar1 downregulated SERCA2 expression in a dose‐dependent manner, as did activation of Sigmar1 by dimemorfan. Using co‐IP, we identified the C‐terminus of Sigmar1 (31–223 aa, located in the cytosol) and the 314–807 aa of SERCA2 that aided the formation of interaction between Sigmar1 and SERCA2. Further molecular docking analysis revealed that the Q615 residue of SERCA2 was of great importance in the interaction between the two proteins. It has been reported that RANKL‐induced calcium signaling is dependent on SERCA2 activity (Yang *et al*, 2009; Kim *et al*, 2013). We speculated that Sigmar1‐induced downregulation of SERCA2 led to impaired calcium signaling, which was confirmed by reduced NFATc1 expression and activation.

There are several mechanisms that lead to protein degradation, such as the ubiquitin–proteasome system, autophagosome system, and ER‐associated degradation. Since Sigmar1 and SERCA2 are both ER‐localized proteins (Schmidt *et al*, 2016; Inoue *et al*, 2019), we speculated that SERCA2 is degraded in the ERAD pathway. Using MG‐132 to suppress the proteasome pathway, degradation of SERCA2 by Sigmar1 was mitigated. Applying NMS‐873 specifically to inhibit VCP function restored SERCA2 downregulation by Sigmar1, since VCP is important for transporting ERAD substrates from the ER to the 26S proteasome (Anderson *et al*, 2015), indicating the ERAD is involved in SERCA2 degradation. As the most conserved E3 ubiquitin ligase in the ERAD system, Hrd1 forms a complex with Sel1L to degrade Sigmar1 in brown adipose tissue (Zhou *et al*, 2020). Hence, we overexpressed Hrd1 with SERCA2 and found that Hrd1 inhibited SERCA2 expression in a dose‐dependent manner. Knockdown of Sel1L by siRNA rescued Sigmar1‐induced SERCA2 degradation, as expected. In this regard, we discovered that Sigmar1 regulated SERCA2 expression by promoting its ubiquitination and degradation through Hrd1/Sel1L‐dependent ERAD.

To evaluate the therapeutic possibility of targeting Sigmar1 *in vivo*, we examined dimemorfan in three pathological mouse models. In acute osteolysis caused by LPS, intraperitoneal injection of dimemorfan resulted in a smoother surface and fewer osteolytic pits on the skull surface. Osteoclast activity was inhibited together with less osteoclast numbers in bone sections. In the second chronic bone‐loss model by ovariectomy surgery, dimemorfan was not given to mice by intraperitoneal injection until 3 weeks to better examine the protective role of dimemorfan in the established bone‐loss model. As expected, the administration of dimemorfan protected OVX mice from excessive bone loss. Osteoporosis caused by ovariectomy was not only mediated by overactivated osteoclast activity but inflammation, serological results indicated that dimemorfan also reduced IL‐1β, IL‐6, and TNF‐α secretion. Apart from osteoporosis, rheumatic arthritis (RA) is also a common disease characterized by inflammatory cell infiltration and bone erosion caused by hyperactivated osteoclast activity. Consequently, we investigated the therapeutic effect of dimemorfan in a CIA model. Consistent with the LPS‐ and OVX‐induced bone‐loss model, dimemorfan exhibited a strong protective effect in the CIA model with a reduced arthritis score, inflammatory infiltration, synovial hyperplasia, and bone erosion. Osteoclast activity was also hampered with dimemorfan treatment. Inflammatory factors were inhibited in this model by dimemorfan too. These *in vivo* mouse models indicated that, in line with the former anti‐inflammatory effect, dimemorfan inhibited osteoclast activity and reduced the inflammatory factors production as well. We supposed this dual effect of activation of Sigmar1 was responsible for the unchanged bone mass in steady conditions between Sigmar1 gKO and WT mice, since under pathological conditions, activation of osteoclast was coupled with different degrees of inflammation. Whereas under steady conditions, there was no excessive osteoclast activity and inflammation, thus Sigmar1 knockout had little effect on the bone mass.

In addition to murine experiments, we examined the anti‐osteoclast effect of dimemorfan on human cells. When hPBMCs from healthy donors underwent osteoclast induction, dimemorfan inhibited this process, along with reduced expression levels of mRNA and proteins related to osteoclastogenesis. Considering the applications in clinical practice for more than 40 years, taking together the above *in vivo* and *in vitro* experiments, Sigmar1 and dimemorfan might be therapeutic targets and choices for the treatment of osteoporosis.

There were some disparate points of view between Sigmar1 and cancer. A few researches suggested that Sigmar1 was highly expressed in breast and lung cancers, and there was a positive correlation between Sigamr1 expression and the cancers’ prognosis and invasive ability (Simony‐Lafontaine *et al*, 2000; Wang *et al*, 2004; Mir *et al*, 2012; Gueguinou *et al*, 2017). Utilization of Sigmar1 ligands may promote tumor progression. Thus, in the future clinical usage of Sigmar1 ligands, such as dimemorfan, or further research, there will be an urgent need to elucidate the possible correction between these drugs and breast cancer to rule out any potential risk.

In conclusion, the findings from our study showed that Sigmar1 exerted a protective effect in pathological conditions and that activation of Sigmar1 inhibited osteoclast formation by promoting SERCA2 degradation through the Hrd1/Sel1L‐dependent ERAD pathway. The Sigmar1 agonist dimemorfan was shown to be beneficial in reducing osteoclast formation and mitigating bone loss in several pathological models. Hence, Sigmar1 agonists, especially dimemorfan, should be considered an anticatabolic agent in bone‐related diseases, in addition to their established role in cough relief.

## Methods

### Reagents

Dimemorfan phosphate (#HY‐B2215, MCE, USA), PRE‐084 hydrochloride (#HY‐18100A, MCE, USA), fluvoxamine maleate (#HY‐B0103A, MCE, USA), MG‐132 (#HY‐13259, MCE, USA), chloroquine diphosphate (#A8628, APExBIO, USA), NMS‐873 (#HY‐15713, MCE, USA), and eeyarestatin I (#SC‐358130, Santa Cruz, USA) were used. Alpha‐modified Eagle’s medium (alpha‐MEM), Dulbecco's modified Eagle’s medium (DMEM), Opti‐MEM, and fetal bovine serum (FBS) were used from Gibco, USA, for cell culture. Recombinant mouse M‐CSF (#CB34, Novoprotein, China) and RANKL (#462‐TEC, R&D, USA), and recombinant human M‐CSF (#C417, Novoprotein, China) and RANKL (#390‐TN‐010, R&D, USA) were acquired.

### Mice

Sigmar1 global knockout mice were obtained from Cyagen Biosciences. All transgenic mice were on a pure C57BL/6 background. DBA/1J mice were purchased from the Shanghai SLAC Laboratory Animal Co., Ltd (Shanghai, China) for collagen‐induced arthritis. All mice were housed in specific pathogen‐free conditions and on a 12 h light/ 12h dark circle at around 22°C. Sigmar1^+/−^ mice were crossed with Sigmar1^+/−^ to generate Sigmar1^+/+^ (wild‐type, WT) and Sigmar1^−/−^ (Sigmar1 gKO) mice. In all experiments, WT littermates of the same sex were used as controls. We euthanized 12‐week‐old transgenic and WT mice for histological and micro‐CT analysis. We also collected blood samples, and serum samples were isolated for serology. Serum ELISAs were performed using a mouse CTX‐I and PINP ELISA kit (Elabscience, China). For dual‐calcein labeling, mice received intraperitoneal injection of calcein (0.25 mg per mouse, #MB4819, Meilunbio, China) 10 and 3 days before euthanasia. All animal studies were approved by the Ethics Committee of Sir Run Run Shaw Hospital, Zhejiang University School of Medicine.

### Cell culture

All cells were cultured in a humidified 5% CO_2_ incubator at 37°C. HEK‐293T cells were obtained from the National Collection of Authenticated Cell Cultures, Chinese Academy of Sciences (#GNHu17), and kept in DMEM with 10% FBS. The absence of mycoplasma contamination was confirmed by PCR and culture, and the cell line identity was authenticated with STR profiling. Murine bone marrow‐derived macrophages (BMMs) were isolated as our previous protocol (Zheng *et al*, 2021). In brief, BMMs were isolated from femurs and tibias of 6‐week‐old C57BL/6 mice and cultured in alpha‐MEM supplemented with 10% FBS and 25 ng/ml mouse M‐CSF for 4 days, which allowed us to the recruit osteoclast precursor cells used in the experiments. In the presence of 50 ng/ml mouse RANKL, murine BMMs were induced to differentiate into osteoclasts for up to 5 days. For murine mesenchymal stem cells (MSCs), isolated bone marrow from 6‐week‐old C57BL/6 mice was cultured with 10% FBS and alpha‐MEM for 4 days, and seeded in 24‐ or 48‐well plates for induction of osteoblasts. Human peripheral blood mononuclear cells (hPBMCs) were purified using Ficoll density gradient centrifugation (#P9011, Solarbio, China) based on the manufacturer’s instructions. The hPBMCs were cultured with 10% FBS and alpha‐MEM, 25 ng/ml human M‐CSF (He *et al*, 2018). For induction of osteoclasts, hPBMCs were treated with 50 ng/ml human RANKL and 25 ng/ml human M‐CSF for up to 12 days. The informed consents were obtained from all the participants, and the blood sample used for research was approved by the Ethics Committee of Sir Run Run Shaw Hospital, Zhejiang University School of Medicine. The human blood study was conducted according to the WMA Declaration of Helsinki and the principles set out in the Department of Health and Human Services Belmont Report.

### Plasmid and siRNA transfection

pENTER‐hSigmar1‐3×FLAG, pcDNA3.4‐mSigmar1‐3×FLAG, and pcDNA3.4‐mSERCA2‐3×MYC were purchased from Vigenebio, China. Different truncated mutants of mouse Sigmar1 and SERCA2 and SERCA2 point mutants were generated using a ClonExpress II One Step Cloning Kit (#C113, Vazyme Biotech Co., Ltd., China). All constructs were verified by DNA sequencing (Tsingke Biotechnology Co., Ltd., China). For siRNA treatment, siRNAs targeting Sel1L and SERCA2 were purchased from RiboBio (Shanghai, China). The sequences of the siRNAs were as follows: Sel1L siRNA (5′‐GGAGAGGAGUUCAAGUUAA‐3′) and SERCA2 siRNA (5′‐GTCCAAGAGTCTCCTTCTA‐3′). HEK‐293T cells or BMMs were seeded in 6‐, 12‐, or 96‐well plates prior to transfection, plasmids or siRNAs were transfected using Lipofectamine™ 3000 Transfection Reagent (#2078159, Invitrogen, USA), and empty vector plasmids or scramble siRNAs were used as negative controls. The culture medium was changed 24 h after transfection. The efficacies of siRNAs were verified using western blot analysis.

### TRAP, alkaline phosphatase, and Alizarin Red staining

For TRAP staining, multinucleated osteoclasts differentiated from murine BMMs or hPBMCs were fixed in 4% paraformaldehyde and stained with TRAP (tartrate‐resistant acid phosphatase) solution (#387A‐1 KT, Sigma‐Aldrich, USA) for 1 h at 37°C. For osteogenic differentiation, MSCs were cultured in an osteogenic medium containing 10 mM b‐glycerophosphate, 50 μM L‐ascorbic acid 2‐phosphate, and 100 nM dexamethasone, the medium was changed every two days. MSCs were induced for 7 days and fixed in 4% paraformaldehyde for ALP staining (#CW0051, CWBIO, China). For mineralization detection, MSCs were induced for 21 days and then fixed in 4% paraformaldehyde. Alizarin Red staining was performed using 2% ARS solution (#ST1078, Beyotime, China).

### Micro‐CT analysis

For micro‐CT analysis, mouse distal femurs and the 5th lumbar spines were fixed with 4% paraformaldehyde for 2 days at room temperature and then scanned by a micro‐CT scanner (#Skyscan 1275, Aartselaar, Belgium) using X‐ray energy of 60 μA/50 kV at a resolution of 9 μm. We selected 0.5 mm from the growth plate to perform qualitative and quantitative trabecular bone analysis. 100 slices from mid‐diaphysis of the femur were used to quantify the cortical bone analysis. The bone morphometric parameters analyzed included BV/TV (trabecular bone volume per total volume), Tb. N (trabecular number), Tb. Th (trabecular thickness), and Tb. Sp (trabecular separation) for trabecular bone and Ct. BV/TV (cortical region BV/TV), and Ct. Th (cortical thickness) for cortical bone. The femurs, skull suture area, and ankles were analyzed for OVX‐, LPS‐, and CIA‐induced bone‐loss models, respectively, as previously described (Zheng *et al*, 2021).

### RNA extraction and quantitative real‐time PCR (RT‐qPCR) assay

We isolated total RNA from BMMs or hPBMCs using an Ultrapure RNA Kit (#CW0581, CWBIO, China) according to the manufacturer’s instructions. The cDNA was synthesized using a HiFi‐Script cDNA Synthesis Kit (#CW2569, CWBIO, China) by reverse transcription. RT‐qPCR quantification was conducted using the cDNA, primers, and the UltraSYBR Mixture (#CW0957, CWBIO, China) according to the manufacturer’s instructions. The relative mRNA expression level was normalized to housekeeping gene (*Gapdh* and *ACTB*) expressions. The primer sequences used in this study are listed in Appendix Table S1.

### Western blot analysis

BMMs, hPBMCs, and HEK‐293T cells were seeded in 6‐well plates (3 × 10^5^ cells/well). After RANKL, dimemorfan stimulation, or plasmid transfection, cells were lysed and proteins were extracted using RIPA (#R0020, Solarbio, China), which contained Phosphatase Inhibitor Cocktail (#CW2383, CWBIO, China) and 100 mM phenylmethanesulfonyl fluoride (PMSF, #ST506, Beyotime, China), and Protease Inhibitor Cocktail (#P8340, Millipore, USA). The lysates were centrifuged and the supernatants were heated at 100°C for 10 min for further analysis. SDS‐polyacrylamide gel electrophoresis was performed as per our previous description (Zhang *et al*, 2020). The bands were detected using an Amersham Imager 600 (GE, USA) and then quantified with ImageJ. Relative antibodies for western blotting were listed below: GAPDH mouse mAb (#AC033, Abclonal, China, 1:1,000), LC3B rabbit Ab (#2775S, CST, USA, 1:1,000), NFATc1 mouse mAb (#sc‐7294, Santa Cruz, USA, 1:1,000), c‐Fos rabbit mAb (#ab222699, Abcam, UK, 1:1,000), SIGMAR1 rabbit mAb (#61994S, CST, USA, 1:1,000), SERCA2 mouse mAb (#sc‐376235, Santa Cruz, USA, 1:1,000), Sel1L mouse mAb (#sc‐377350, Santa Cruz, USA, 1:1,000), HRD1/SYVN1 rabbit pAb (#13473‐1‐Ap, Proteintech, China, 1:1,000), Myc‐tag rabbit pAb (#R1208‐1, HUABIO, China, 1:1,000), Flag‐tag rabbit mAb (#14793S, CST, USA, 1:1,000). Secondary antibodies were anti‐rabbit IgG HRP‐linked antibody (#7074S, CST, USA, 1:5,000), anti‐mouse IgG HRP‐linked antibody (#7076S, CST, USA, 1:5,000).

### Resorption pit experiment

The hydroxyapatite resorption experiment was performed as previously described (Kartner *et al*, 2010). BMMs were seeded on a 96‐well hydroxyapatite‐coated plate (#3989, Corning Inc., USA) with three replicates. BMMs were treated with 25 ng/ml M‐CSF and 50 ng/ml RANKL in the presence or absence of 10 μM dimemorfan for 72 h. Subsequently, each well was washed with 10% sodium hypochlorite to erase cells. Then, areas of the resorption pit were captured with a microscope and quantified with ImageJ software.

### PAGE gel silver staining

Silver gel staining was performed using PAGE Gel Silver Staining Kit (#G7210, Solarbio, China) as per the manufacturer’s instructions.

### Cellular immunofluorescence staining

For anti‐NFATc1 immunofluorescence staining, primary BMMs on 12‐well slides were treated with 25 ng/ml M‐CSF and 50 ng/ml RANKL for 2 days in the presence or absence of 10 μM dimemorfan. After fixation with 4% paraformaldehyde for 20 min, BMMs were permeabilized with 2‰ Triton X‐100, blocked with 5% bovine serum albumin in PBS, and incubated with NFATc1 primary antibody (1:100) overnight at 4°C. The cells were then washed with PBS and incubated with the secondary goat anti‐mouse IgG H&L antibody (Alexa Fluor 488) (#ab150113, Abcam, UK, 1:400) for 30 min. Nuclei were stained with DAPI. The images were captured by confocal microscopy (Nikon Eclipse TI, Japan).

### Co‐immunoprecipitation (co‐IP) assay

HEK‐293T cells and BMMs were seeded on 6‐cm dishes and treated differently for 48 h according to experimental assignments. The cells were then immersed in lysis buffer supplemented with 1 mM PMSF, 1 mM dithiothreitol (DTT) (#HY‐15917, MCE, USA), and a protease inhibitor cocktail. Cell lysates were immunoprecipitated with Flag‐tagged mouse mAb (#M1403‐2, HUABIO, China, 1:100), Myc‐tagged mouse mAb (#30601ES60, YEASON, China, 1:100), SIGMAR1 rabbit mAb (#61994S, CST, USA, 1:100), rabbit (DA1E) mAb IgG isotype control (#3900S, CST, USA) or mouse (G3A1) mAb IgG1 isotype control (#5415S, CST, USA) at 4°C overnight, and then the mixtures were incubated with protein A/G beads for 3 h at 4°C. The beads were washed and centrifuged five times with PBS containing protease inhibitor cocktail at 4°C, resolved with 10% SDS buffer, and then analyzed by western blotting.

### Immunoprecipitation and mass spectrum analysis

HEK‐293T cells were seeded on 6‐cm dishes and transfected with vector plasmid or Flag‐tagged Sigmar1 plasmid. As mentioned above, the cells were lysed and immunoprecipitated with anti‐IgG or anti‐Flag antibody after 48 h. Successful immunoprecipitation of Sigmar1 was verified using silver staining and western blot. The immunoprecipitant protein was then subjected to liquid chromatography with tandem mass spectrometry for proteomics analysis (Novogene, China).

### Immunofluorescence staining and immunohistochemistry staining

Immunofluorescence staining and immunohistochemistry staining were performed following standard procedures. For immunofluorescence staining, bone sections were incubated with anti‐SOST antibody (#21933‐1‐AP, Proteintech, China, 1:100) at 4°C overnight and washed with PBS for three times, and incubated with the secondary goat anti‐mouse IgG H&L antibody (1:400) for 30 min. Nuclei were stained with DAPI. The images were captured by a fluorescence microscope (Nikon, Japan). For immunohistochemistry staining, sections were submerged with sodium citrate at 55°C overnight for antigen retrieving. After being blocked with BSA for 1 h, the sections were incubated with anti‐osteocalcin antibody (#23418‐1‐AP, Proteintech, China, 1:100) at 4°C overnight and washed with PBS for three times and incubated with anti‐rabbit secondary antibody (ZsBio, China). Sections were washed with PBS for three times and stained with DAB solution (ServiceBio, China) at RT for 5–10 min. The images were captured by microscope (Nikon, Japan).

### Homology modeling and molecular docking

The crystal structures of SERCA2 (ATP2A2) and SIGMAR1 homotrimers were obtained from the RCSB Protein Database Bank, PDB code: 5ZTF (chain A, positions 314–807) (Inoue *et al*, 2019) and PDB code: 5HK1 (chain A, B, C) (Schmidt *et al*, 2016), respectively. The structures of the two biopolymers were analyzed and prepared for the docking experiment. All ligands and water molecules in the crystal structures were removed. A docking study of the binding mode between the SERCA2 and the SIGMAR1 homotrimer was conducted using the HDOCK server (Huang & Zou, 2008, 2014; Yan *et al*, 2017a, 2017b, 2020). In HDOCK, a lower docking score means a better predicted binding mode. According to the docking score provided by the HDOCK server, the predicted binding mode with the lowest docking score was selected to analyze the detailed interaction network between the two proteins. The best predicted binding mode was visualized, analyzed, and mapped using the PyMOL program (http://www.pymol.org).

### Measurement of SERCA2 activity

WT BMMs were seeded on 6‐well plates and treated with or without dimemorfan (10 mM) for 48 h. After treatment, BMMs were collected and lysed by sonication in ice‐cold PBS. Cell suspensions were then centrifuged, and the supernatant was used to measure SERCA2 activity. SERCA2 activity was detected using a kit from NJJCBIO (#A070‐4, NJJCBIO, Nanjing, China) according to the manufacturer’s instructions.

### Bone marrow transfer experiment

The bone marrow transfer experiment was conducted as described previously(Lin *et al*, 2018). 6‐week‐old WT female C57BL/6J mice were subjected to sublethal irradiation, and WT or Sigmar1 gKO bone marrow cells were transferred to these mice through tail vein injection. Those mice who were not injected with any cells dead within the first‐week post‐irradiation. 6 weeks after the transfer, we collected mice blood for genotype identification, Sigmar1 gKO cells had positive mut bands with negative wt bands whereas WT cells had wt bands with negative mut bands. Those success transferred with WT or Sigmar1 gKO bone marrow cells were then subjected to OVX surgery and euthanized 6 weeks later for radiological and histological evaluation.

### Ovariectomy (OVX)‐induced osteoporosis model

The OVX‐induced bone‐loss model was established according to a previous description (Yu *et al*, 2014). Briefly, 12‐week‐old C57BL/6 female WT or Sigmar1 gKO mice were anesthetized and subjected to either sham or bilateral ovariectomy. For dimemorfan treatment, WT OVX mice were randomly divided into two groups, and the dimemorfan‐treated group received an intraperitoneal injection of dimemorfan at a dose of 7 mg/kg three times every week. The control OVX group instead received PBS, and all injections were initiated in the fourth week. For AAV treatment, OVX or sham mice were injected via the intramedullary route with AAV expressing GFP or Sigmar1 in both femurs and tibias. Six or eight weeks after model establishment, all mice were euthanized, and femurs and tibias were collected and fixed in 4% paraformaldehyde for micro‐CT and bone histomorphometric analysis.

### LPS‐induced calvarial osteolysis model

The animal model was established as described (Wu *et al*, 2017). Briefly, LPS‐induced calvarial osteolysis was conducted using 12‐week‐old male C57BL/6 mice. The mice were anesthetized and subcutaneously injected with 25 mg/kg LPS (#L2630, Sigma‐Aldrich, USA) and divided into three groups: sham, LPS‐induced saline injection, and LPS‐induced dimemorfan injection. PBS or dimemorfan (7 mg/kg) was injected intraperitoneally every other day starting 2 days after surgery. All mice were euthanized at the end of 7 days. Craniums were collected and fixed in 4% paraformaldehyde for micro‐CT and bone histomorphometry analysis.

### Collagen‐induced arthritis (CIA) model

Eight‐week‐old male DBA/1J mice were immunized with chick type II collagen solution (#20011, Chondrex, USA), as previously described (Brand *et al*, 2007). Briefly, Chick type II collagen solution (4 mg/ml in 0.05 M acetic acid) was emulsified with Complete Freund’s Adjuvant (#7001, Chondrex, USA) (ratio 1:1). The mice were injected intradermally 1.5 cm from the base of the tail with 0.1 ml of the emulsion. After 21 days, Chick type II collagen solution was emulsified with Incomplete Freund’s Adjuvant (#7002, Chondrex, USA). For a booster injection, the mice were injected with 0.05 ml of emulsion 0.5 cm from the base of the tail. After the booster injection, dimemorfan was injected intraperitoneally at a dose of 7 mg/kg three times every week. The mice were then euthanized 21 days after the booster injection for micro‐CT, bone morphological, and histomorphometric analysis (Ierna *et al*, 2010). The arthritis severity was determined by visual inspection (Song *et al*, 2019).

### Study design and statistics of animal experiments

No statistical methods were used to predetermine sample size. At least 5 animals were used per group in all animal experiments. Animals were allocated into groups randomly, and the surgeries, harvest, and analysis of animals were blind to reduce subjective bias. No inclusion or exclusion criteria were conducted on the animals.

### Statistical analysis

All datasets are represented as the mean ± standard deviation (SD) with individual data points. Statistical analyses were performed using SPSS 19.0 (SPSS, Chicago). The normal distribution of the data was determined by the Shapiro–Wilk test. Statistical differences were assessed using the Student’s *t*‐test or one‐way ANOVA followed by the Tukey’s post hoc analysis where appropriate. For those non‐normally distributed data, nonparameter tests were used. *P* values were indicated in the figure legends, and a *P* value < 0.05 was considered statistically significant. All experiments were performed at least three times independently.

## Author contributions

**Xiaoan Wei:** Writing—original draft; Project administration. **Zeyu Zheng:** Validation; Project administration. **Zhenhua Feng:** Validation; Project administration. **Lin Zheng:** Methodology; Project administration. **Siyue Tao:** Methodology; Project administration. **Bingjie Zheng:** Project administration. **Bao Huang:** Project administration. **Xuyang Zhang:** Project administration. **Junhui Liu:** Writing—review & editing. **Yilei Chen:** Writing—review & editing. **Wentian Zong:** Resources. **Zhi Shan:** Writing—review & editing. **Shunwu Fan:** Supervision; Funding acquisition. **Jian Chen:** Data curation; Supervision; Funding acquisition; Writing—review & editing. **Fengdong Zhao:** Conceptualization; Formal analysis; Supervision; Funding acquisition; Writing—review & editing.

In addition to the CRediT author contributions listed above, the contributions in detail are:

XW, ZZ, and ZF performed the experiments. LZ, ST, BZ, BH, and XZ aided in animal experiments. JL, YC, WZ, and ZS helped in editing of the manuscript. SF revised the manuscript. JC and FZ conceived and supervised all experiments and the writing of the manuscript.

## Disclosure and competing interests statement

The authors declare that they have no conflict of interest.

## For more information

- OMIM: https://omim.org/
- PyMol: http://www.pymol.org/
- GenBank: https://www.ncbi.nlm.nih.gov/gene/
- PrimerBank: https://pga.mgh.harvard.edu/primerbank/
- UniProt: https://www.uniprot.org/
- UbiBrowser: http://ubibrowser.ncpsb.org.cn/ubibrowser/
- PLMD: http://plmd.biocuckoo.org/

## Supporting information



Appendix

Expanded View Figures PDF

Source Data for Expanded View and Appendix

Source Data for Figure 4

Source Data for Figure 5

Source Data for Figure 7

## Data Availability

This study includes no data deposited in external repositories.
