# Supplementary material for: Sigma‐1 receptor attenuates osteoclastogenesis by promoting ER‐associated degradation of SERCA2
Source: EMBO Mol Med. 2022 May 25;14(7):e15373. doi: 10.15252/emmm.202115373 (PMC9260208; doi:10.15252/emmm.202115373)
Supplement: Supplementary file 1 — Appendix [file EMMM-14-e15373-s004.pdf]

Sigma-1 receptor attenuates osteoclastogenesis by promoting ER-associated degradation of SERCA2

Xiaoan Wei[1,2†], Zeyu Zheng[1,2†], Zhenhua Feng[1,2†], Lin Zheng[1,2], Siyue Tao[1,2], Bingjie Zheng[1,2], Bao Huang[1,2], Xuyang Zhang[1,2], Junhui Liu[1,2], Yilei Chen[1,2], Wentian Zong[1,2], Zhi Shan[1,2], Shunwu Fan[1,2], Jian Chen[1,2*], Fengdong Zhao[1,2*]

[1]Department of Orthopaedic Surgery, Sir Run Run Shaw Hospital, Zhejiang University School of Medicine, Hangzhou, China.

[2]Key Laboratory of Musculoskeletal System Degeneration and Regeneration Translational Research of Zhejiang Province.

[†]These authors contributed equally to this work.

*Fengdong Zhao and Jian Chen are corresponding authors.

Correspondence:

Fengdong Zhao, zhaofengdong@zju.eu.cn

Jian Chen, chenjian-bio@zju.edu.cn

Table of contents

Appendix table S1: Specific primer sequences for RT–qPCR

Appendix Figure S1. Sigmar1 deletion has no influence on bone mass under steady conditions.

Appendix Figure S2. Sigmar1 deletion promotes osteoclastogenesis in vitro.

Appendix Figure S3. Overexpression of Sigmar1 rescues OVX-induced bone loss.

Appendix Figure S4. Dimemorfan inhibits osteoclast gene expression and function.

Table S1: Specific primer sequences for RT–qPCR

Gene	Stream	Sequence
mouse *Gapdh*	Forward	5’-GGAGAGTGTTTCCTCGTCCC-3’
	Reverse	5’-ATGAAGGGGTCGTTGATGGC-3’
mouse *Nfatc1*	Forward	5’-CCCGTCACATTCTGGTCCAT-3’
	Reverse	5’-CAAGTAACCGTGTAGCTGCACAA-3’
mouse *Ctsk*	Forward	5’-GCTCACAGTAGCCACGCTT-3’
	Reverse	5’-AACGCCGAGAGATTTCATCCA-3’
mouse *C-fos*	Forward	5’-GGGAATGGTGAAGACCGTGT-3’
	Reverse	5’-CCGTTCCCTTCGGATTCTCC-3’
mouse *Dcstamp*	Forward	5’-TTTCCACGAAGCCCTAGCTG-3’
	Reverse	5’-GCGTTCCTACCTTCACGGAG-3’
mouse *Acp5*	Forward	5’-AAGAGATCGCCAGAACCGTG-3’
	Reverse	5’-TTCCAGCCAGCACATACCAG-3’
mouse *Atp6v0d2*	Forward	5’-CAGAGCTGTACTTCAATGTGGAC-3’
	Reverse	5’-AGGTCTCACACTGCACTAGGT-3’
mouse *Atp2a2*	Forward	5’-GAGAACGCTCACACAAAGACC-3’
	Reverse	5’-CAATTCGTTGGAGCCCCAT-3’
human *ACTB*	Forward	5’-AGAGCTACGAGCTGCCTGAC-3’
	Reverse	5’-AGCACTGTGTTGGCGTACAG-3’
human *NFATC1*	Forward	5’-ACTCAGAGGCTCCGAACTC-3’
	Reverse	5’-AAAGTCATCGAGGGGCGTG-3’

human *C-FOS*	Forward	5’-GGGGCAAGGTGGAACAGTTA-3’
	Reverse	5’-AGGTTGGCAATCTCGGTCTG-3’
human *ATP6V0D2*	Forward	5’-CCCTCTATCCAACCTTCGGC-3’
	Reverse	5’-ACGCTCGTAAAACACGTCCT-3’
human *CTSK*	Forward	5’-CCCGCAGTAATGACACCCTT-3’
	Reverse	5’-AAAGCCCAACAGGAACCACA-3’
human *DCSTAMP*	Forward	5’-CCACAGAGGTGTTGTCCTCC-3’
	Reverse	5’-CCACAAGGGCCCAAAAATCG-3’
human *ACP5*	Forward	5’-CGTATTCTCTGACCGCTCCC-3’
	Reverse	5’-TCTTGAAGTGCAGGCGGTAG-3’

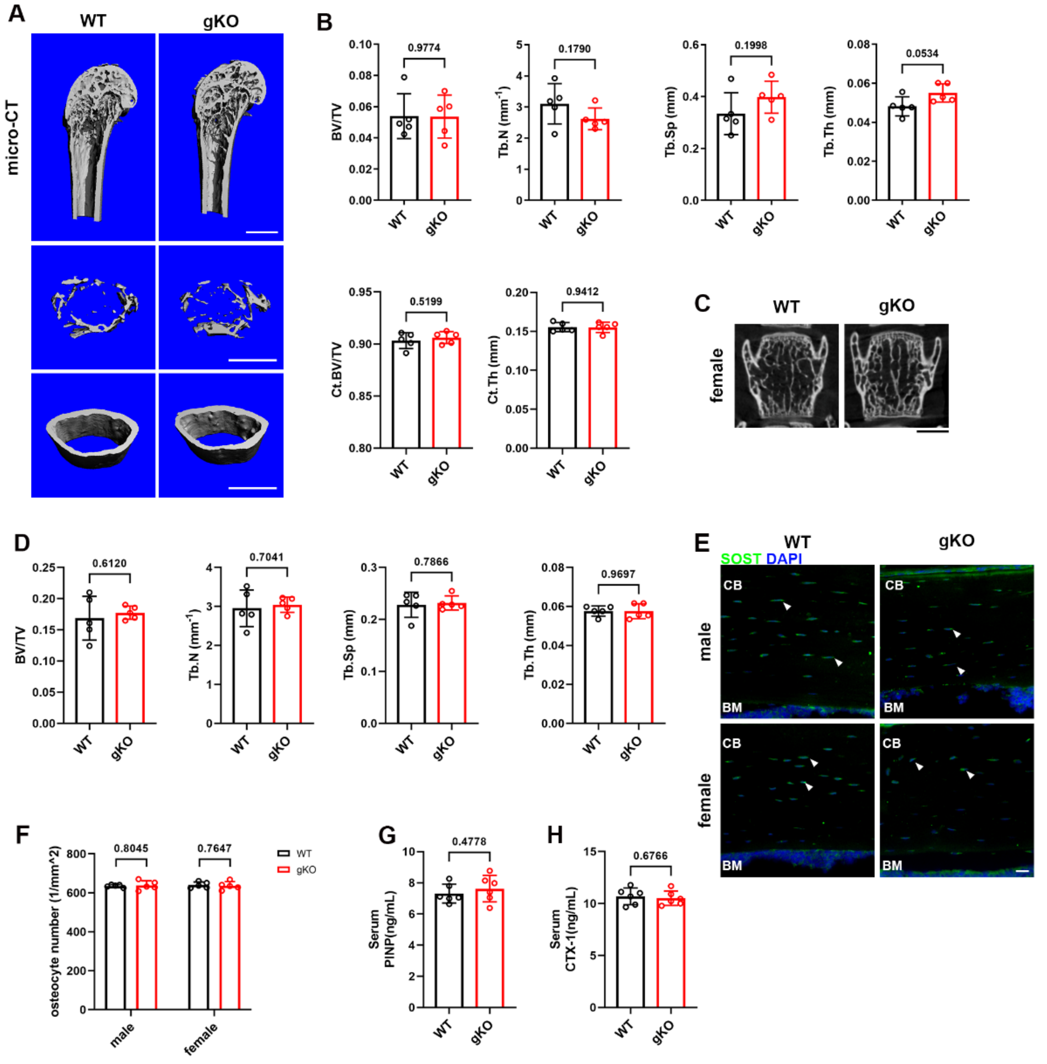

Appendix Figure S1. Sigmar1 deletion has no influence on bone mass under steady conditions.

(A) Micro-CT reconstructed images of proximal and femur from female WT and Sigmar1 gKO mice. Scale bars, 1 mm.

(B) Quantification of bone volume per tissue volume (BV/TV), trabecular number (Tb. N), trabecular separation (Tb. Sp), trabecular thickness (Tb. Th), cortical region BV/TV (Ct. BV/TV) and cortical thickness (Ct. Th, mm) (n = 5 biological replicates).

(C) Coronal images of the fifth lumbar spine. Scale bars, 1 mm.

(D) Quantification of trabecular bone parameters of lumbar spine (n = 5 biological replicates).

(E) Immunofluorescence images of SOST in femurs. Arrows indicate SOST-positive osteocytes. Scale bars, 50 μm.

(F) Quantification of SOST-positive osteocyte in bone sections (n = 5 biological replicates).

(G and H) Serum PINP (procollagen I N-terminal propeptide) and CTX-I (C-terminal telopeptide of type I collagen) concentrations measured by ELISA in female Sigmar1 gKO mice and their WT littermates (n = 6 biological replicates).

Data information: All results are representative data generated from at least three independent experiments. Data are presented as mean ± SD. Unpaired two-tailed Student's t-test (B, D and F-H) was used for statistical analysis.

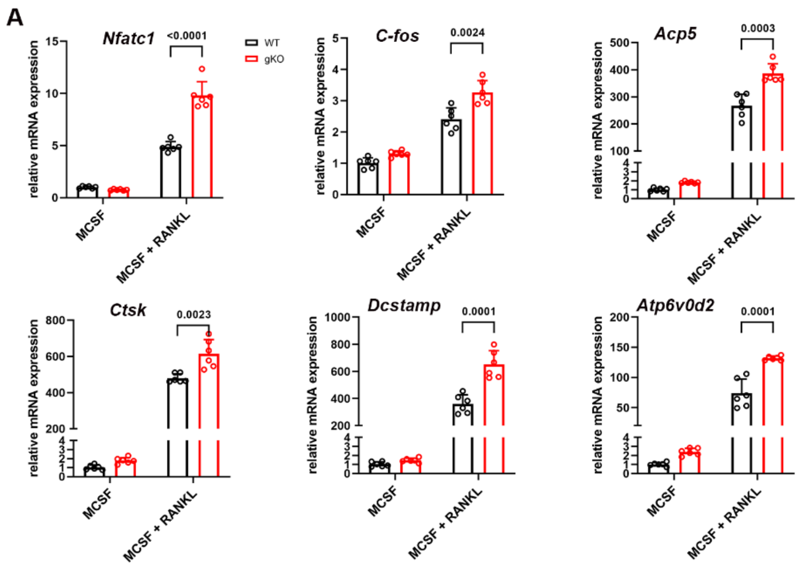

Appendix Figure S2. Sigmar1 deletion promotes osteoclastogenesis in vitro.

(A) BMMs from WT and Sigmar1 gKO mice were induced to differentiate into osteoclasts for 2 days, and the relative mRNA levels of marker genes were evaluated by RT–qPCR (n = 6 biological replicates).

Data information: All results are representative data generated from at least three independent experiments. Data are presented as mean ± SD. Unpaired two-tailed Student's t-test (A) was used for statistical analysis.

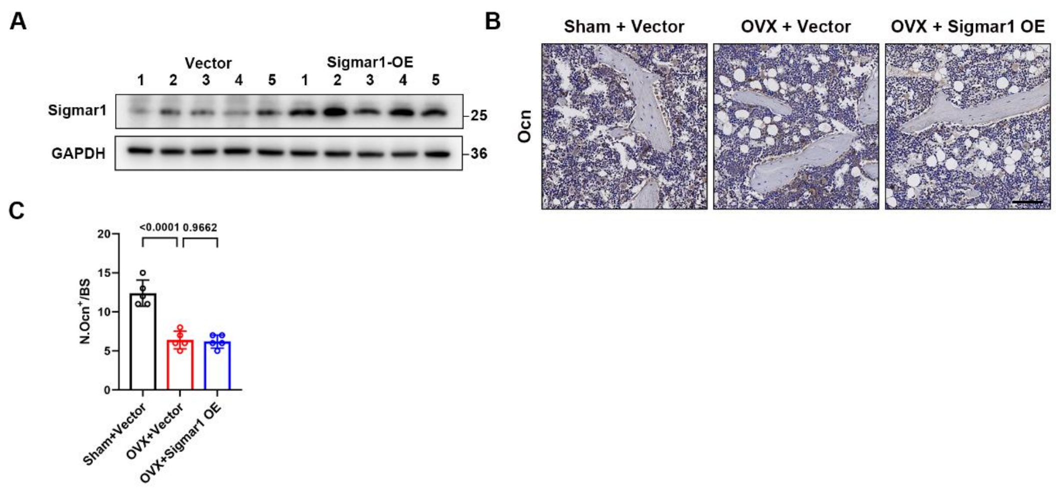

Appendix Figure S3. Overexpression of Sigmar1 rescues OVX-induced bone loss.

(A) Overexpression of Sigmar1 in BMMs by AAV injection was verified by western blotting. (B and C) Immunohistochemistry staining of Ocn in femur sections (B) and quantification of Ocn-positive osteoblast number on trabecular bone surface (C) (n = 5 biological replicates). Scale bars, 200 μm.

Data information: All results are representative data generated from at least three independent experiments. Data are presented as mean ± SD. One-way ANOVA with Tukey's multiple comparisons test (C) was used for statistical analysis.

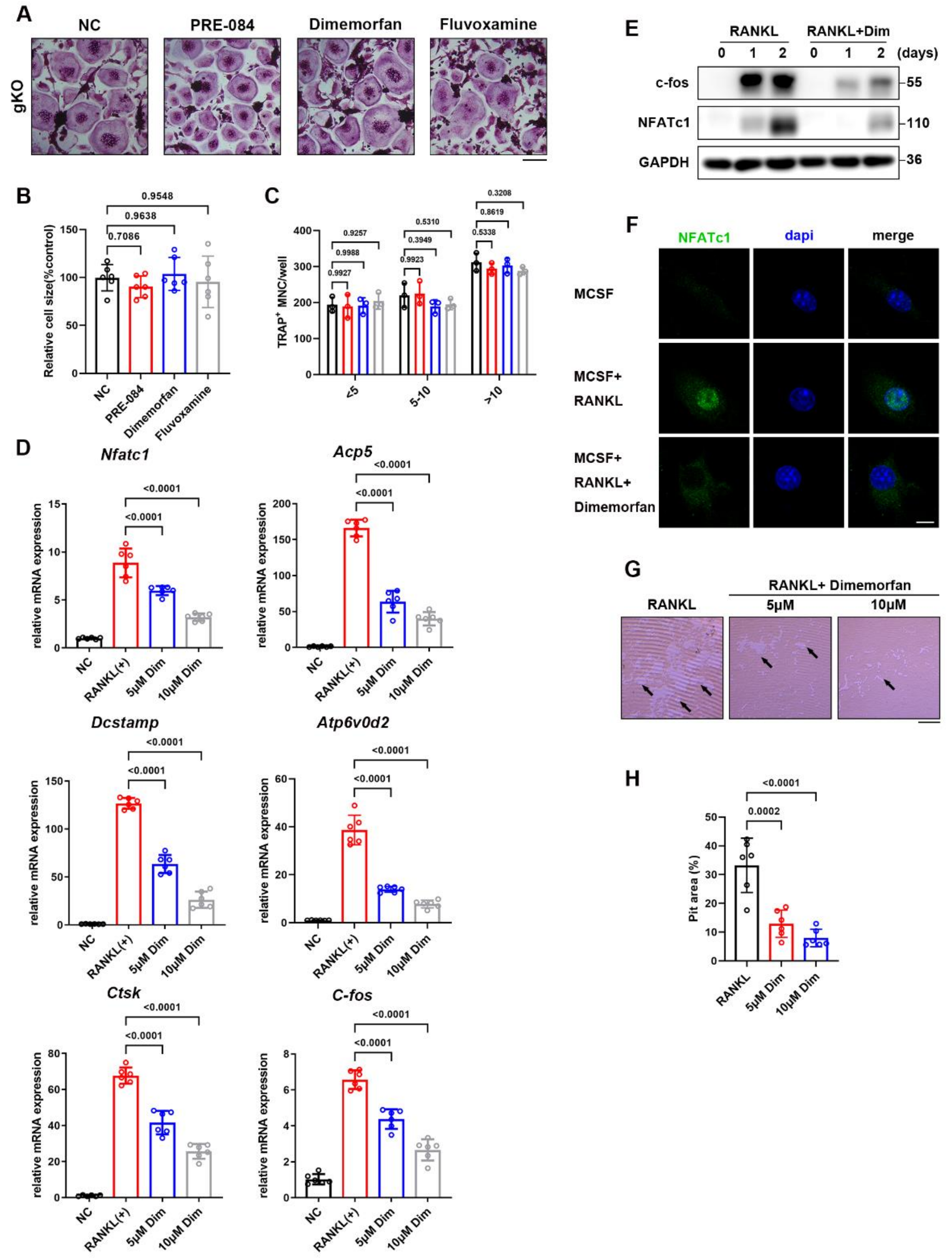

Appendix Figure S4. Dimemorfan inhibits osteoclast gene expression and function.

(A) TRAP staining to detect osteoclastogenesis of Sigmar1 gKO BMMs treated with different Sigmar1 agonists (10μM) or vector. Scale bars, 200 μm.

(B and C) Quantification of the size and nuclei numbers of TRAP-positive multinuclear cells (n = 6 biological replicates).

(D) Expression of marker genes for osteoclastogenesis in primary BMMs treated with 5 or 10 μM dimemorfan for 2 days (n = 6 biological replicates).

(E) Western blot analysis of NFATc1 and c-fos expression in BMMs treated in the presence or absence of 10 μM dimemorfan for indicated time.

(F) Confocal images of NFATc1 immunofluorescence of BMMs with indicated treatment for 48 hours. Scale bars, 10 μm.
(G and H) Representative images and quantification of the relative pit resorption area of hydroxyapatite-coated plates. BMMs were seeded on hydroxyapatite-coated plates and treated with 50 ng/mL RANKL in the presence of PBS or dimemorfan for 3 days. Black arrows indicate bone resorption areas (n = 6 biological replicates). Scale bars, 500 μm.
Data information: All results are representative data generated from at least three independent experiments. Data are presented as mean ± SD. One-way ANOVA with Tukey's multiple comparisons test (B-D and H) was used for statistical analysis.
