# Supplementary material for: Sigma‐1 receptor attenuates osteoclastogenesis by promoting ER‐associated degradation of SERCA2
Source: EMBO Mol Med. 2022 May 25;14(7):e15373. doi: 10.15252/emmm.202115373 (PMC9260208; doi:10.15252/emmm.202115373)
Supplement: Supplementary file 2 — Expanded View Figures PDF [file EMMM-14-e15373-s006.pdf]

Expanded View Figures

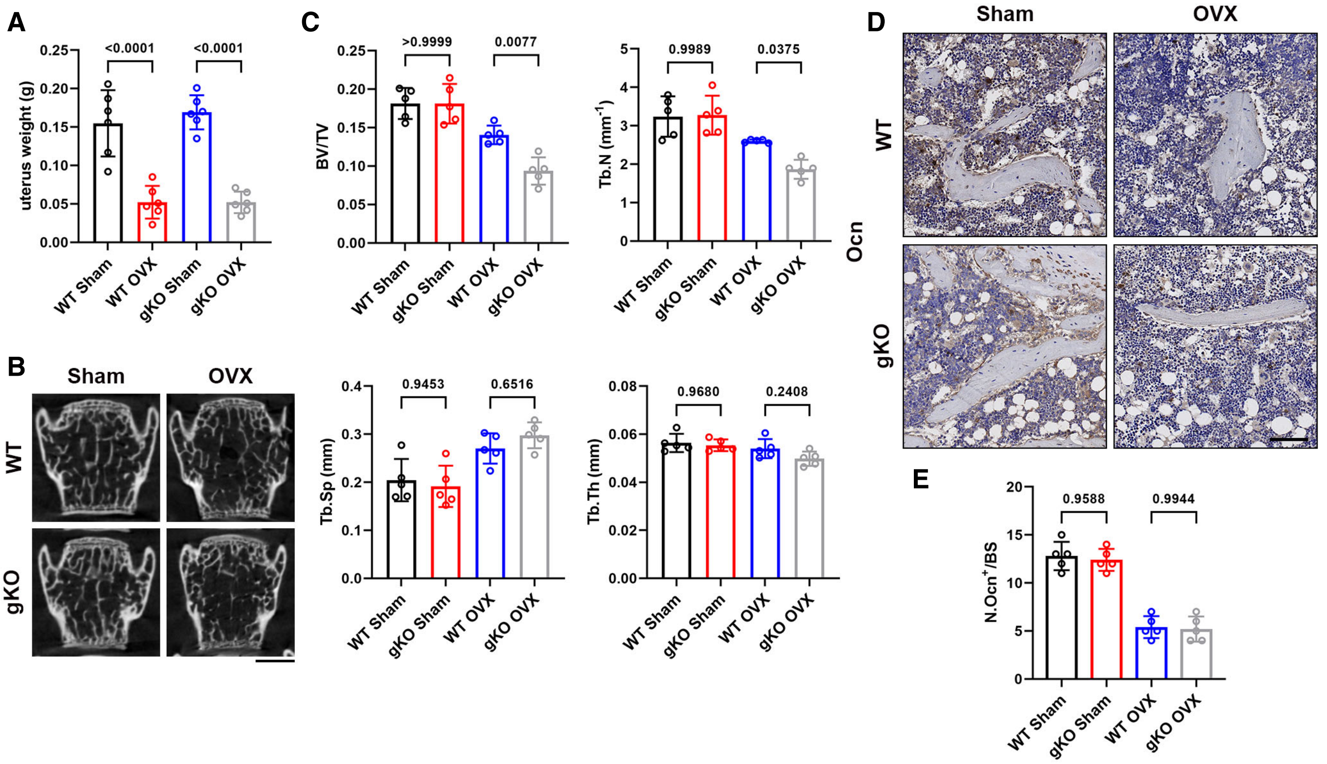

Figure EV1. Sigmar1 deletion results in severe osteoporosis in the OVX model and promotes osteoclastogenesis *in vitro*.

A Uterus weight from different groups (n = 6 biological replicates).

B Coronal images of the fifth lumbar spine. Scale bars, 1 mm.

C Quantification of trabecular bone parameters of lumbar spine (n = 5 biological replicates).

D, E Immunohistochemistry staining of osteocalcin (Ocn) in femur sections (D) and quantification of Ocn-positive osteoblast on trabecular bone surface (E) (n = 5 biological replicates).

Data information: All results are representative data generated from at least three independent experiments. Data are presented as mean ± SD. The one-way ANOVA with the Tukey's multiple comparison test (A, C, and E) was used for statistical analysis.
Source data are available online for this figure.

© 2022 The Authors

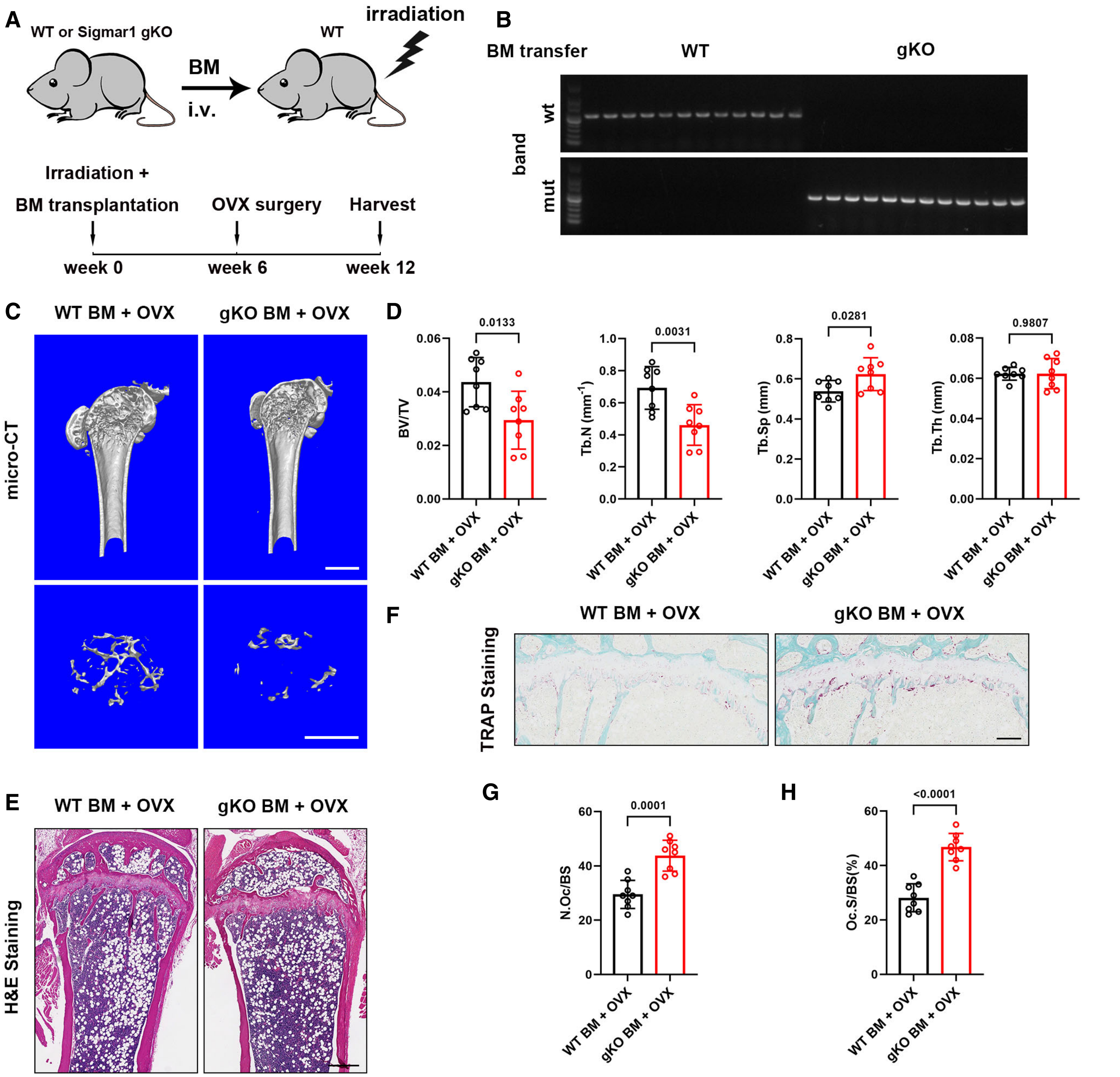

Figure EV2. Bone marrow transfer of Sigmar1 gKO cells exacerbates OVX-induced osteoporosis.

A The schematic illustrates the protocol for bone marrow transfer experiment and OVX surgery.

B PCR bands for identifying success transfer of WT and Sigmar1 gKO bone marrow cells. Sigmar1 gKO cells had negative wt bands (upper), and positive mut bands (lower) and WT cells had the opposite results.

C Micro-CT images of the proximal femur from female ovariectomized mice that transferred with WT or Sigmar1 gKO bone marrow cells previously. Scale bars, 1 mm.

D Quantification of bone volume per tissue volume (BV/TV), trabecular number (Tb. N), trabecular separation (Tb. Sp), and trabecular thickness (Tb. Th) (n = 8 biological replicates).

E H&E staining of femur sections. Scale bars, 200 μm.

F TRAP staining of femur sections. Scale bars, 200 μm.

G, H Quantification of osteoclast number per bone surface (N. Oc/BS) and percentage of osteoclast surface per bone surface (Oc. S/BS) (n = 8 biological replicates).

Data information: All results are representative data generated from at least three independent experiments. Data are presented as mean ± SD. Unpaired two-tailed Student's t-test (D and G and H) was used for statistical analysis.

 © 2022 The Authors

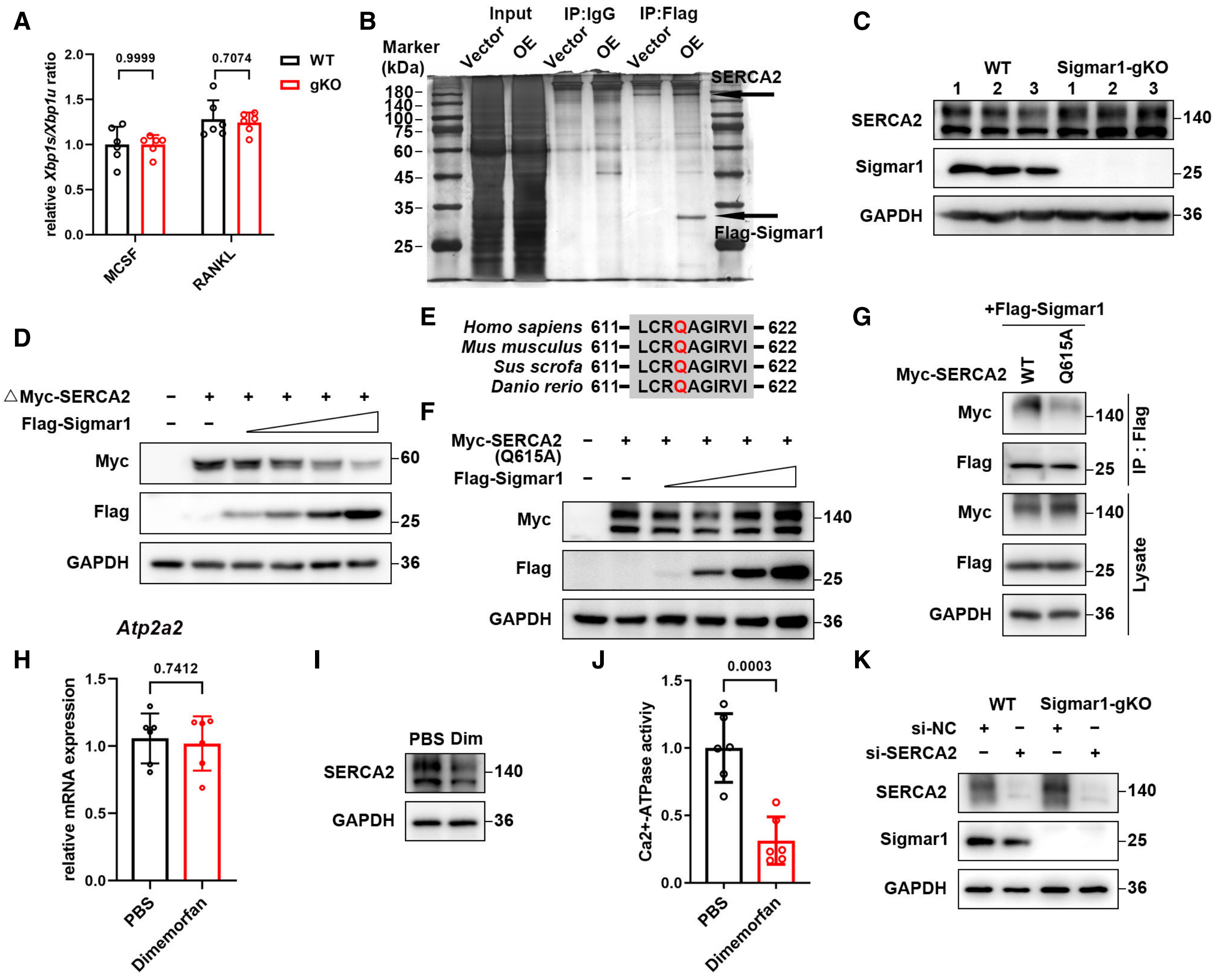

Figure EV3. Sigmar1 interacts with SERCA2 and mediates its degradation.

- A Relative *xbp1* splicing level of WT or Sigmar1 gKO BMMs during osteoclast formation measured by RT-qPCR (n = 6 biological replicates).
- B Immunoprecipitation of flag-Sigmar1 interacting protein was visualized by silver gel staining.
- C Western blots showing relative SERCA2 expression in WT and Sigmar1 gKO BMMs.
- D Western blots showing truncated SERCA2 (314–807 aa) expression in HEK-293T cells transfected with different amounts of Sigmar1 plasmids. The cells were transfected with 1 μg truncated SERCA2 plasmid and Sigmar1 plasmid (0.125, 0.25, 0.5, and 1 μg).
- E Sequence alignment of glutamine residues from SERCA2 orthologs of different species.
- F Western blots showing Q615A mutant full-length SERCA2 expression in HEK-293T cells in with different amounts of Sigmar1 plasmids. The cells were transfected with 1 μg Q615A mutant full-length SERCA2 plasmid and Sigmar1 plasmid (0.125, 0.25, 0.5, and 1 μg).
- G Interactions between Sigmar1 and WT or Q615 mutants of full-length SERCA2 were detected by Co-IP assays.
- H Relative mRNA expression of SERCA2 in BMMs treated with PBS or 10 μM dimemorfan for 2 days (n = 6 biological replicates).
- I Western blots showing SERCA2 expression in BMMs treated with PBS or 10 μM dimemorfan for 2 days.
- J Relative SERCA2 activity in BMMs treated with PBS or dimemorfan was detected by the Ca2+-ATPase assay (n = 6 biological replicates).
- K Knock down of SERCA2 by siRNA in BMMs was verified by western blotting.

Data information: All results are representative data generated from at least three independent experiments. Data are presented as mean ± SD. The unpaired two-tailed Student's *t*-test (A, H and J) was used for statistical analysis.
Source data are available online for this figure.

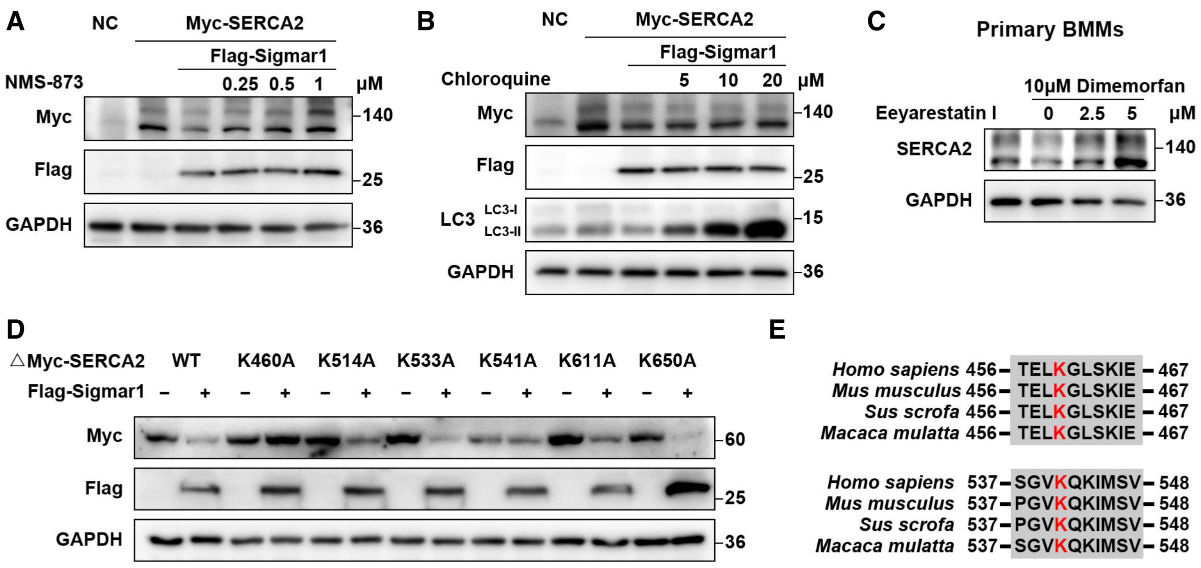

Figure EV4. Sigmar1 mediates SERCA2 degradation through the Hrd1/Sel1L-dependent ERAD pathway.

A, B Western blots showing SERCA2 expression in HEK-293T cells with or without Sigmar1 co-transfection treated with NMS-873 or chloroquine at the indicated concentration. All inhibitors were applied to cells 8 h prior to protein collection.

C Western blots showing SERCA2 expression in BMMs treated with dimemorfan or vehicle for 48 h. Eeyarestatin I was added 8 h prior to cell harvest at indicated concentration.

D HEK-293T cells were transfected with different truncated SERCA2 lysine mutants and Sigmar1 and then subjected to western blot analysis.

E Sequence alignment of Ub sites in SERCA2 orthologs of different species.

Data information: All results are representative data generated from at least three independent experiments.
Source data are available online for this figure.

 © 2022 The Authors

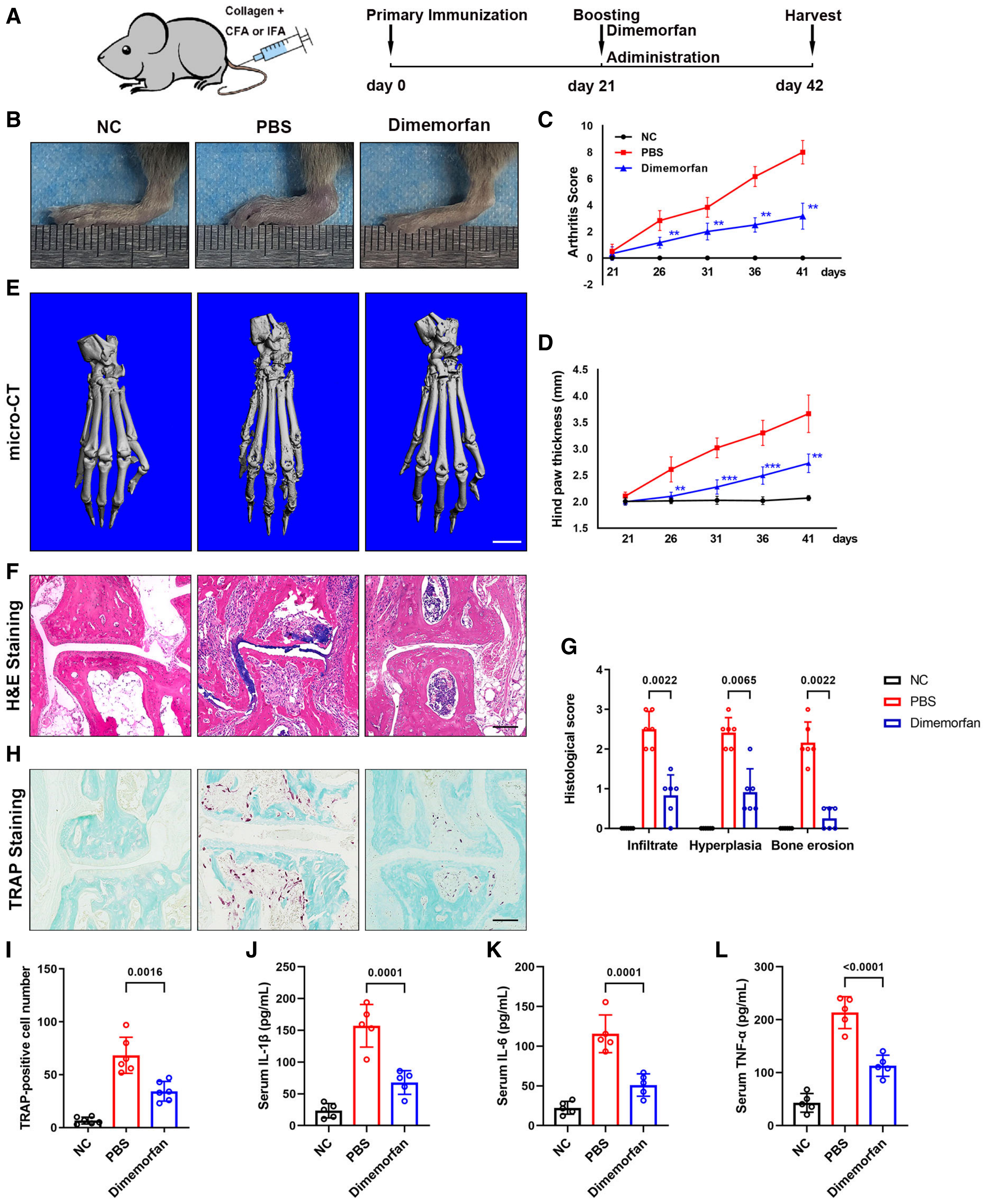

A
Collagen +
CFA or IFA
Primary Immunization
Boosting
Dimemorfan
Adiministration
Harvest
day 0
day 21
day 42
B
NC
PBS
Dimemorfan
C
Arthritis Score
NC
PBS
Dimemorfan
days
D
Hind paw thickness (mm)
days
E
micro-CT
F
H&E Staining
G
Histological score
0.0022
0.0065
0.0022
Infiltrate
Hyperplasia
Bone erosion
H
TRAP Staining
I
TRAP-positive cell number
0.0016
J
Serum IL-1β (pg/mL)
0.0001
K
Serum IL-6 (pg/mL)
0.0001
L
Serum TNF-α (pg/mL)
<0.0001

Figure EV5.

◀ **Figure EV5. Dimemorfan alleviates joint destruction and osteoclast activity in CIA mice.**

A The schematic illustrates the protocol for CIA induction and dimemorfan treatment.
B Photographs of representative gross lesions in the hind limbs of CIA mice for clinical assessment.
C, D Arthritis in PBS-treated and dimemorfan-treated mice was induced by chicken type II collagen injection ($n = 6$ mice per group and per time point). After the second immunization, the arthritis score (C) and hind paw thickness (D) were evaluated every 5 days.
E Micro-CT images of paws from CIA mice with different treatments. Scale bars, 2 mm.
F H&E staining of ankle joints from the three groups. Scale bars, 50 μm.
G Cell infiltrate (left), synovial hyperplasia (middle), and bone/cartilage erosion (right) of sections from different groups were analyzed ($n = 5$ biological replicates).
H TRAP staining of ankle joints from the three groups. Scale bars, 50 μm.
I The number of TRAP-positive cells per field of tissue sections stained with TRAP at 100× magnification was analyzed ($n = 6$ biological replicates).
J–L Serum IL-1β, IL-6, and TNF-α concentrations measured by ELISA in three groups ($n = 5$ biological replicates).

Data information: All results are representative data generated from at least three independent experiments. Data are presented as mean ± SD. The one-way ANOVA with the Tukey's multiple comparison test (D and I–L) and nonparameter test (C and G) were used for statistical analysis. $^{**}P < 0.01$. $^{***}P < 0.001$ versus PBS group.

© 2022 The Authors
