# Supplementary material for: Sigma‐1 receptor attenuates osteoclastogenesis by promoting ER‐associated degradation of SERCA2
Source: EMBO Mol Med. 2022 May 25;14(7):e15373. doi: 10.15252/emmm.202115373 (PMC9260208; doi:10.15252/emmm.202115373)

Fig 4B

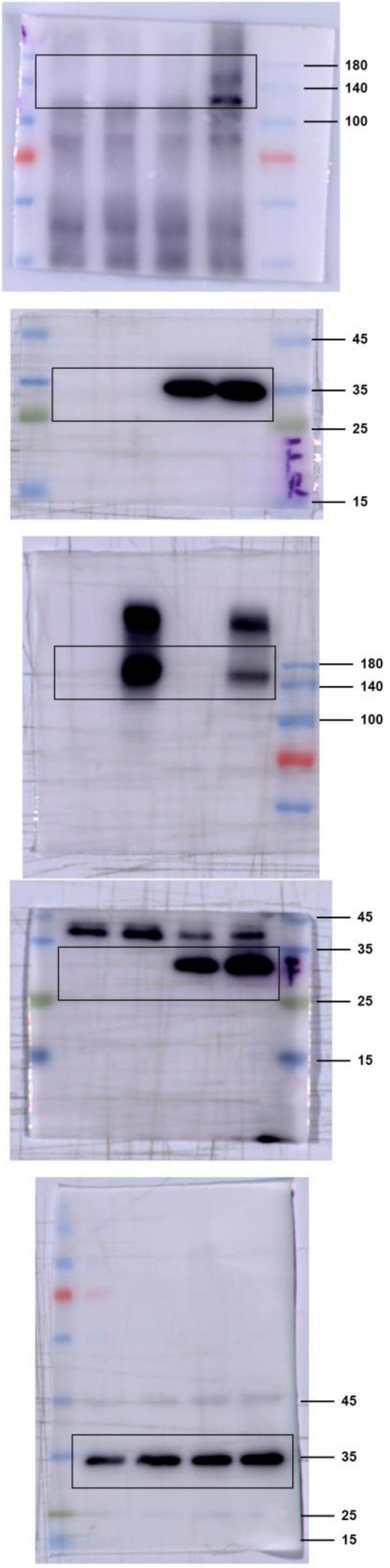

IP:Flag IB:Myc

IP:Flag IB:Flag

Lysate IB:Myc

Lysate IB:Flag

Lysate IB:GAPDH

Fig 4C

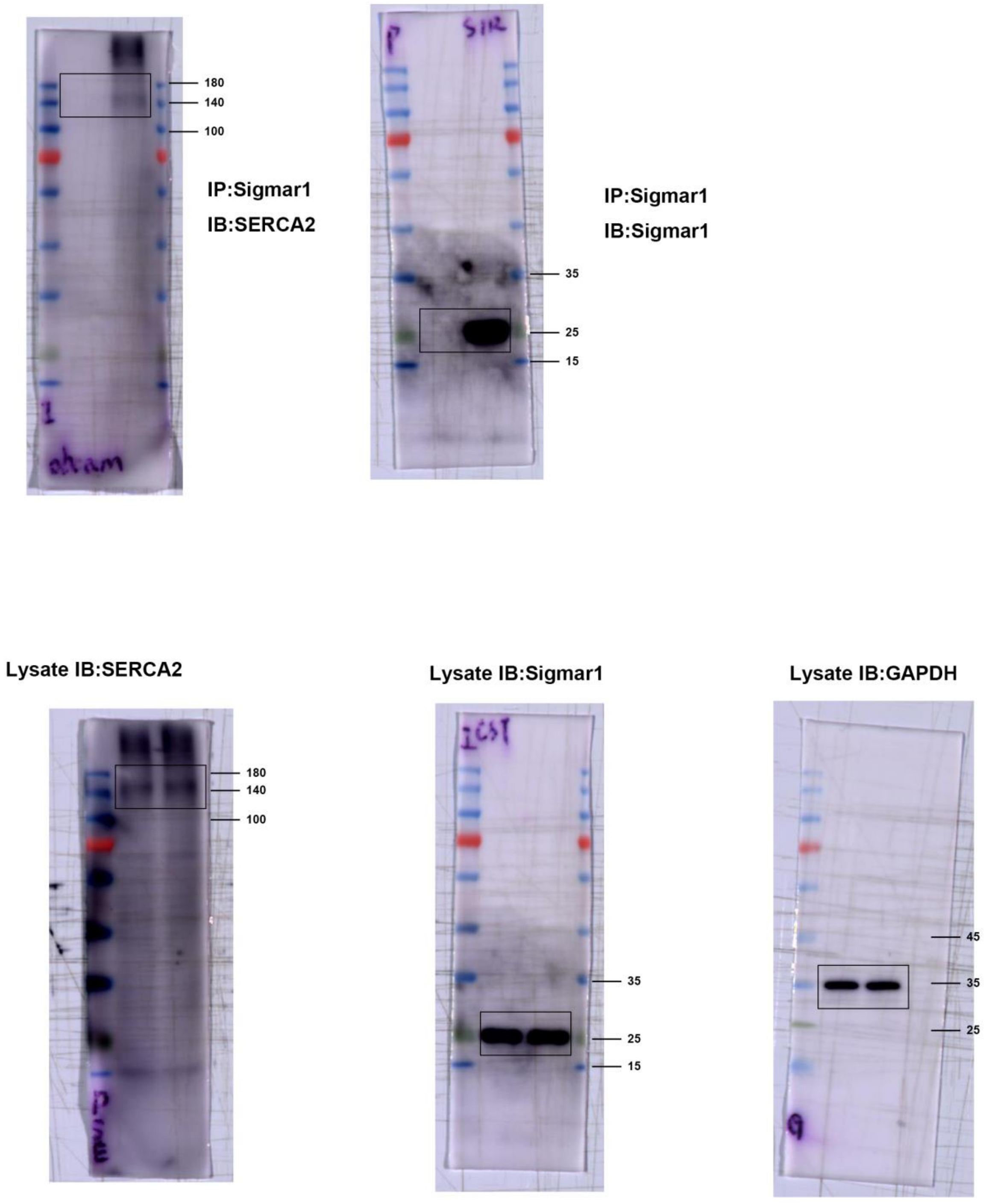
180
140
100
IP:Sigmar1
IB:SERCA2
sham
P
SIR
35
25
15
IP:Sigmar1
IB:Sigmar1
Lysate IB:SERCA2
180
140
100
Lysate IB:Sigmar1
35
25
15
Lysate IB:GAPDH
45
35
25

Fig 4D

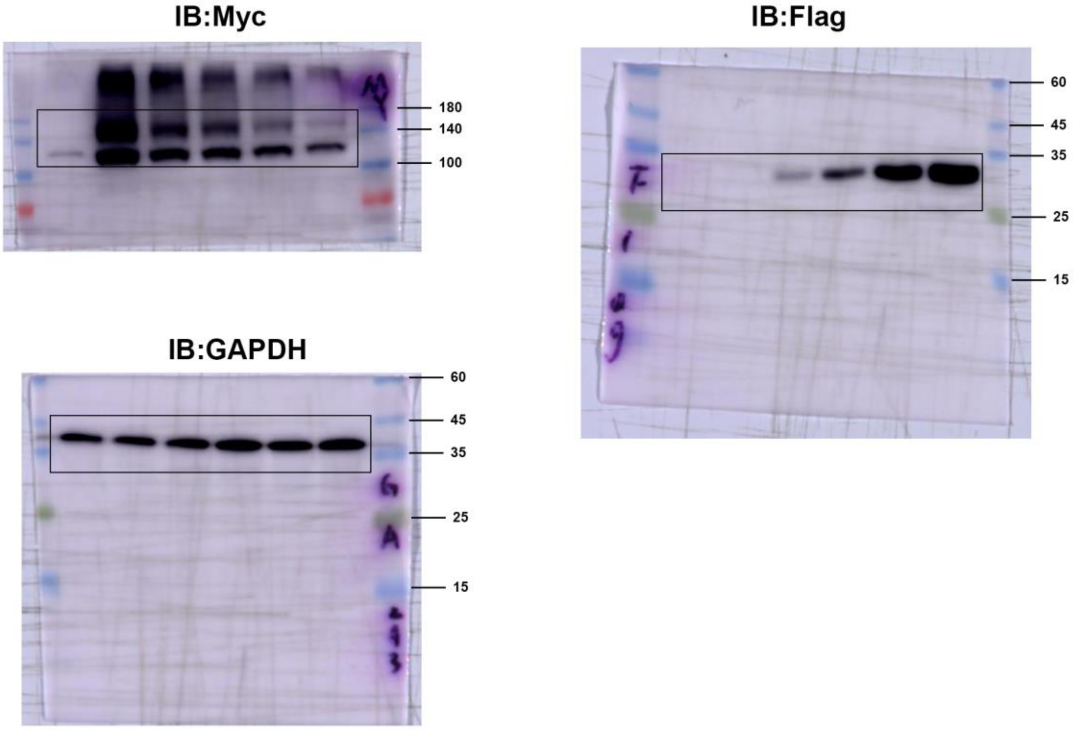

Fig 4E

IP:Flag
IB:Myc

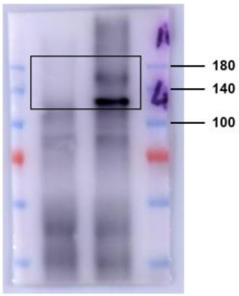

IP:Flag
IB:Flag

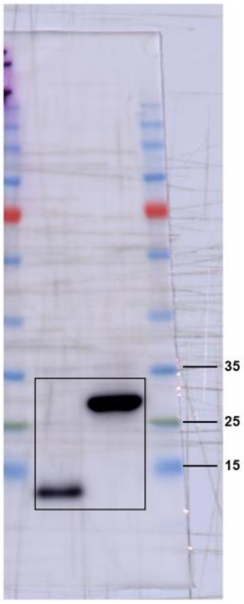

Lysate IB:Myc

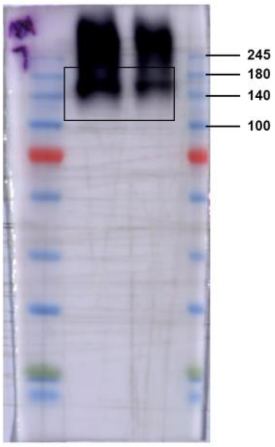

Lysate IB:Flag

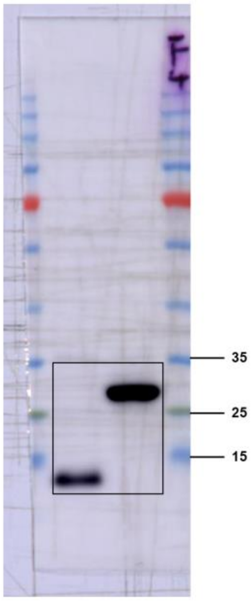

Lysate IB:GAPDH

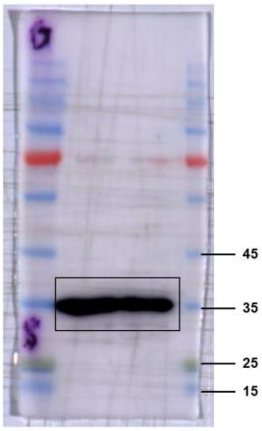

Fig 4F

IP:Flag
IB:Myc

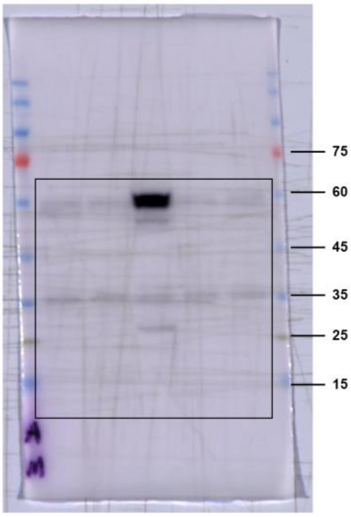

IP:Flag
IB:Flag

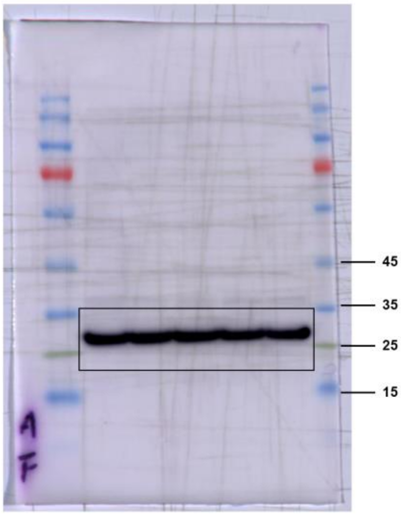

Lysate IB:Myc

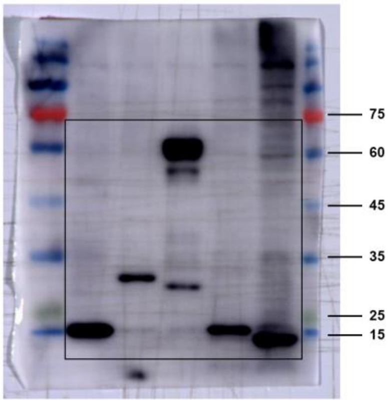

Lysate IB:Flag

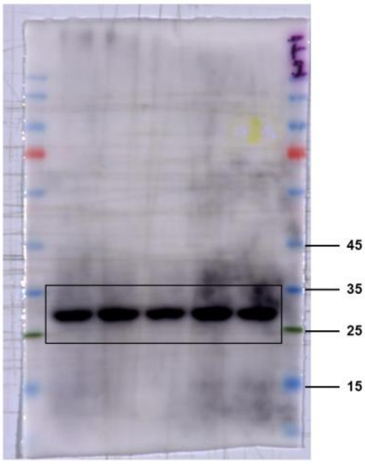

Lysate IB:GAPDH

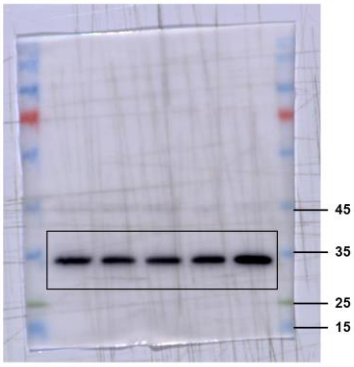

Fig 4H

IB:Myc

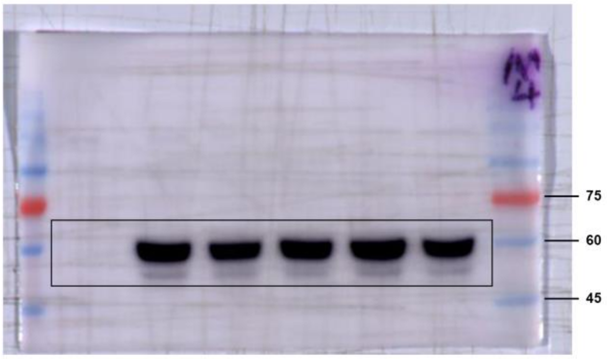

IB:Flag

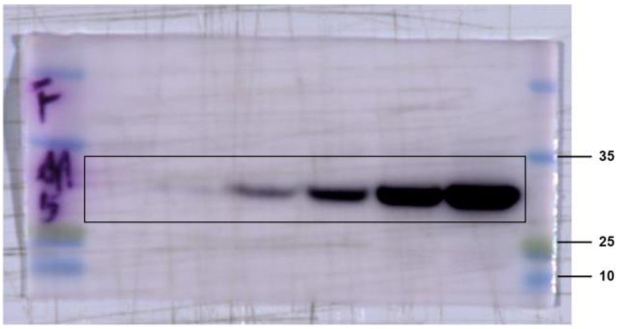

IB:GAPDH

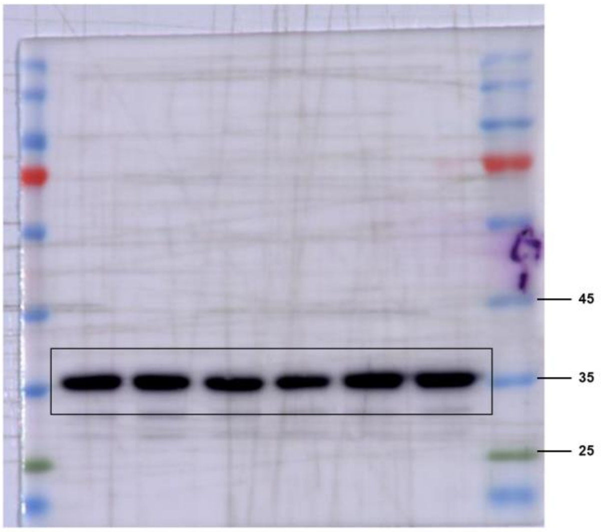

Fig 4I

IP:Flag

IB:Myc

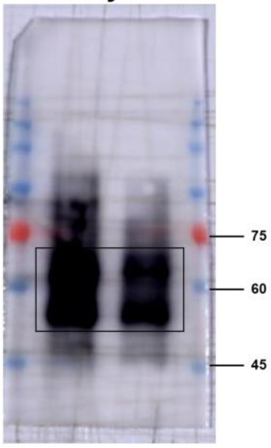

IP:Flag

IB:Flag

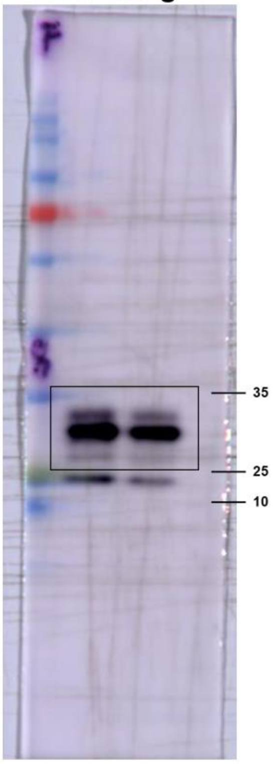

Lysate IB:Myc

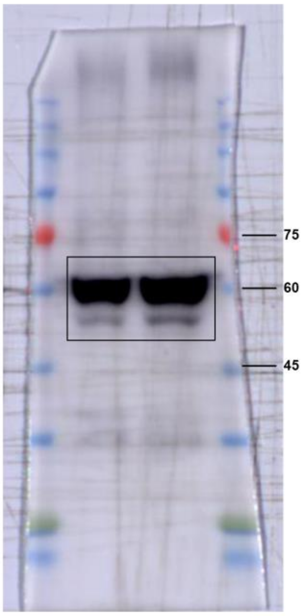

Lysate IB:Flag

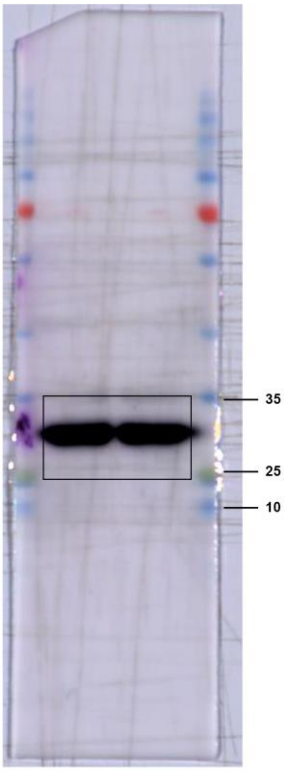

Lysate IB:GAPDH

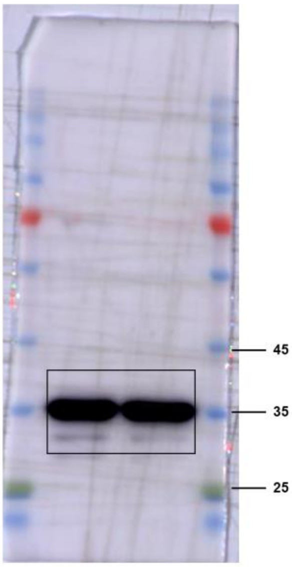

Supplement: Supplementary file 4 — Source Data for Figure 4 [file EMMM-14-e15373-s001.pdf]
