# Supplementary material for: Sigma‐1 receptor attenuates osteoclastogenesis by promoting ER‐associated degradation of SERCA2
Source: EMBO Mol Med. 2022 May 25;14(7):e15373. doi: 10.15252/emmm.202115373 (PMC9260208; doi:10.15252/emmm.202115373)
Supplement: Supplementary file 5 — Source Data for Figure 5 [file EMMM-14-e15373-s002.pdf]

Fig 5A

IB:Myc

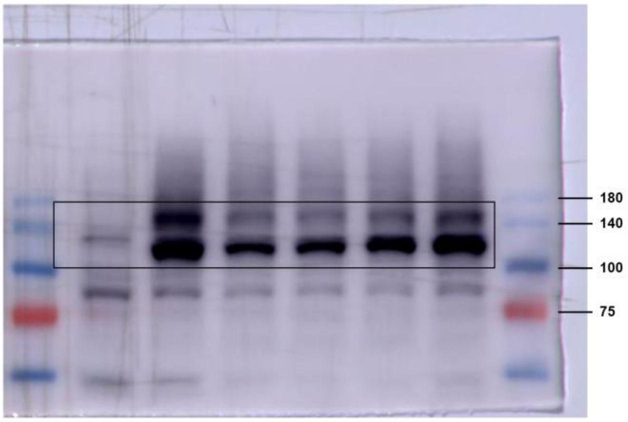

IB:Flag

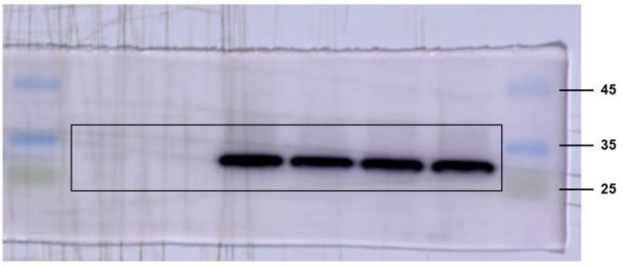

IB:GAPDH

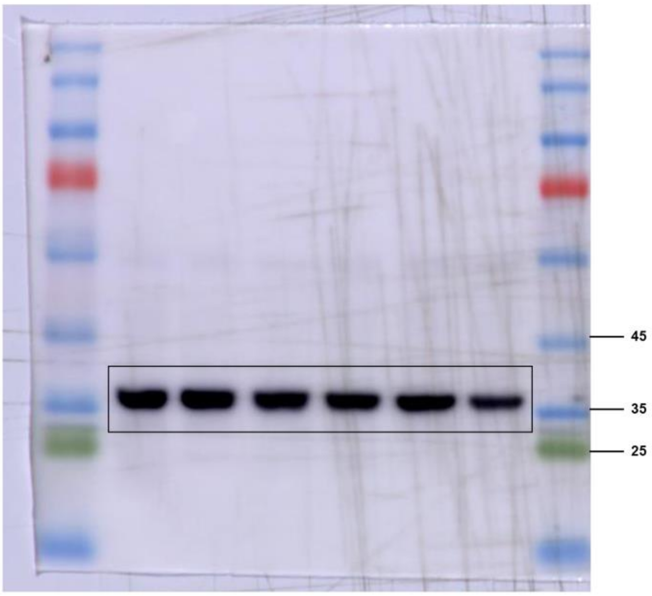

Fig 5B

IB:Myc

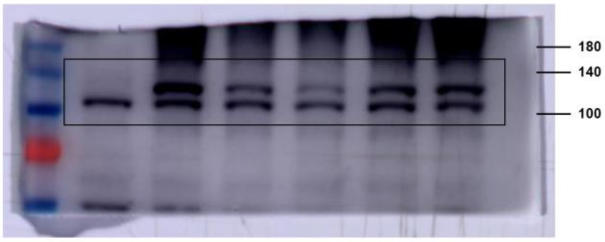

IB:Flag

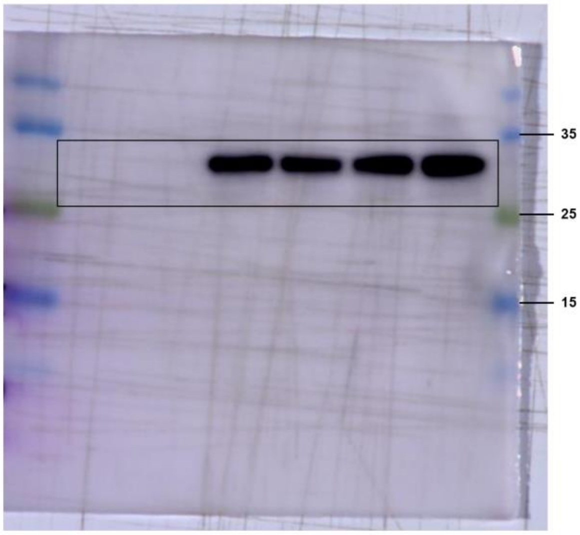

IB:GAPDH

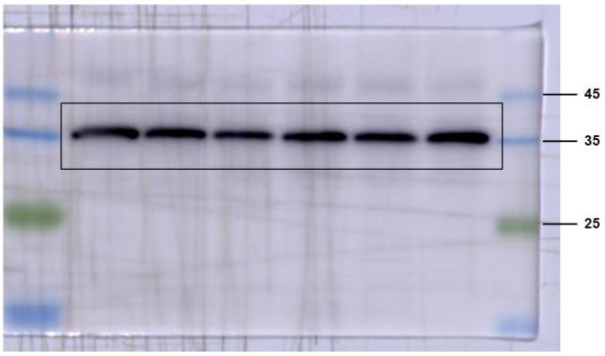

Fig 5C

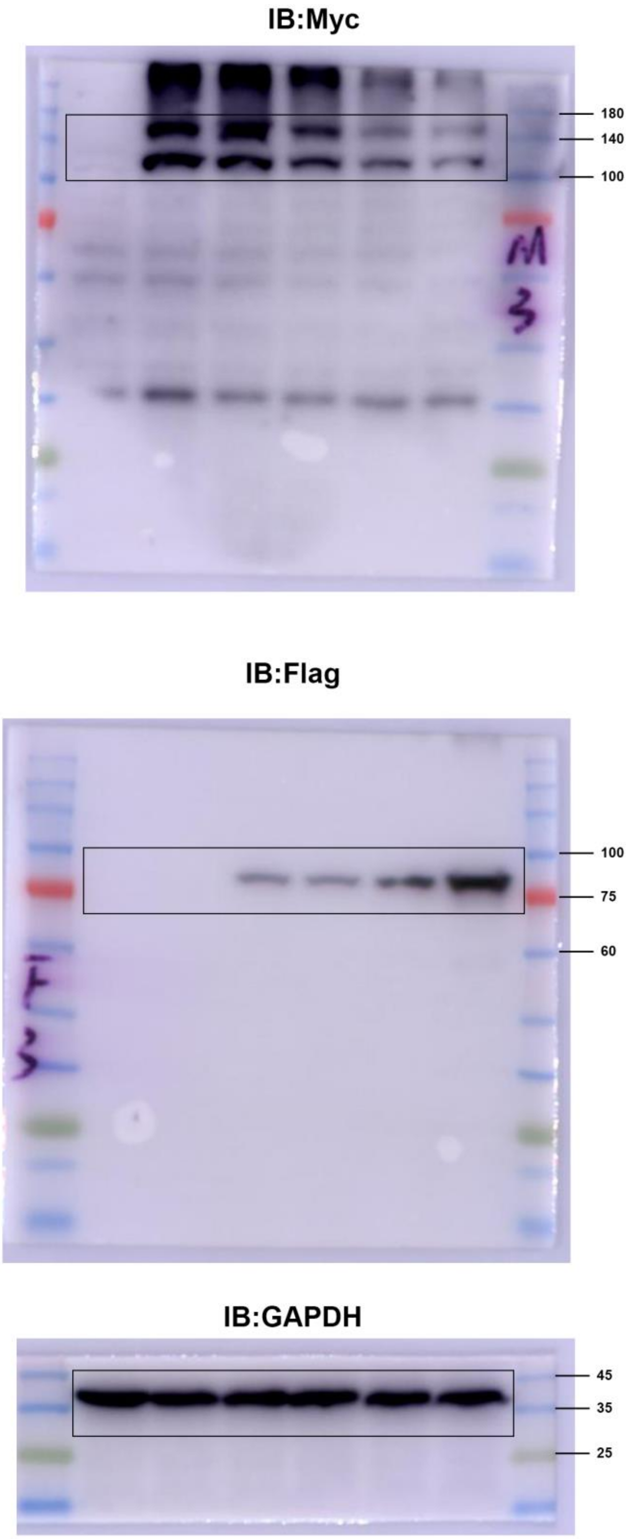

Fig 5D

IB:Myc

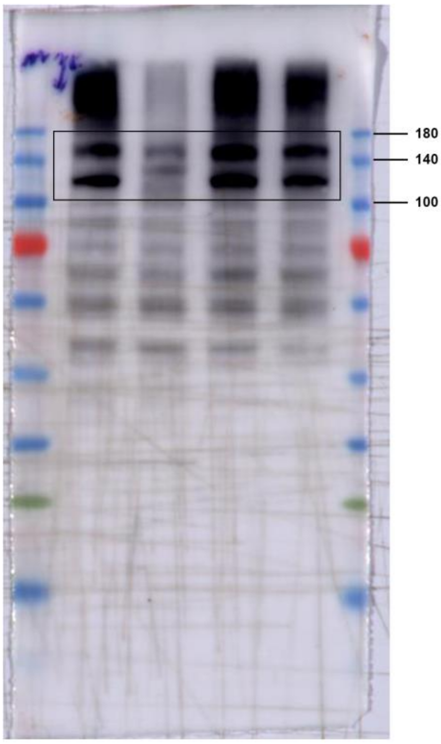

IB:Sel1L

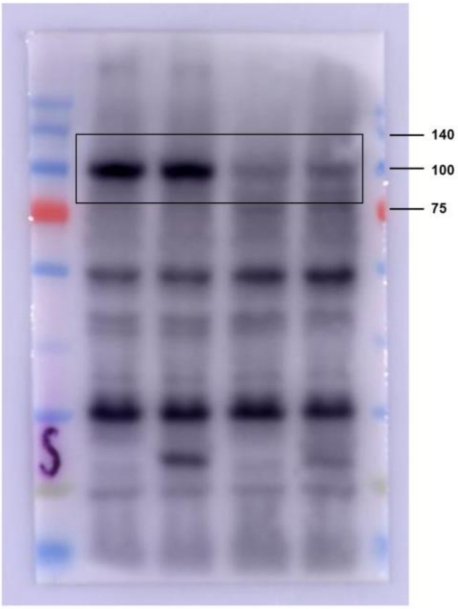

IB:Flag

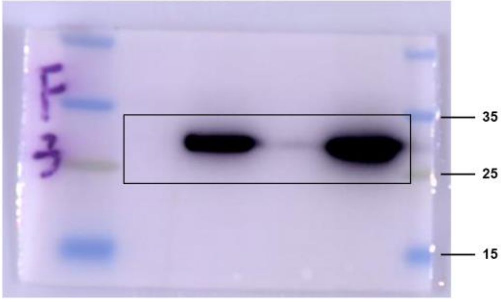

IB:GAPDH

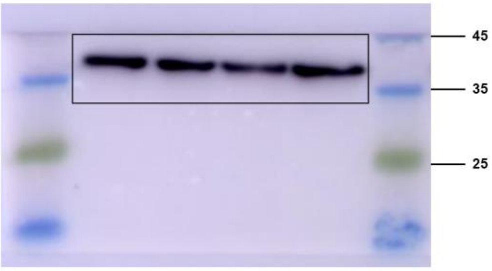

Fig 5E

IP:Flag

IB:HA

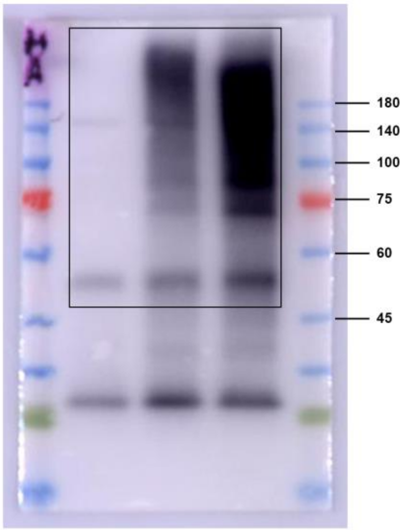

Lysate IB:Flag

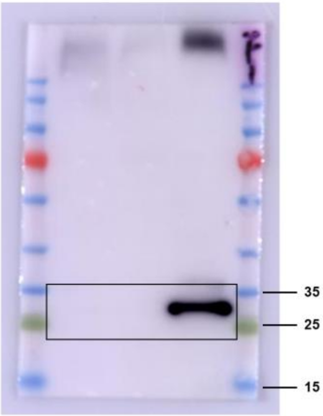

Lysate IB:Myc

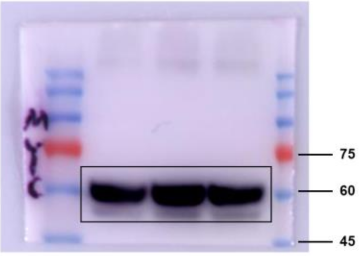

Lysate IB:GAPDH

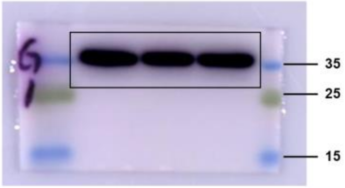

Fig 5F

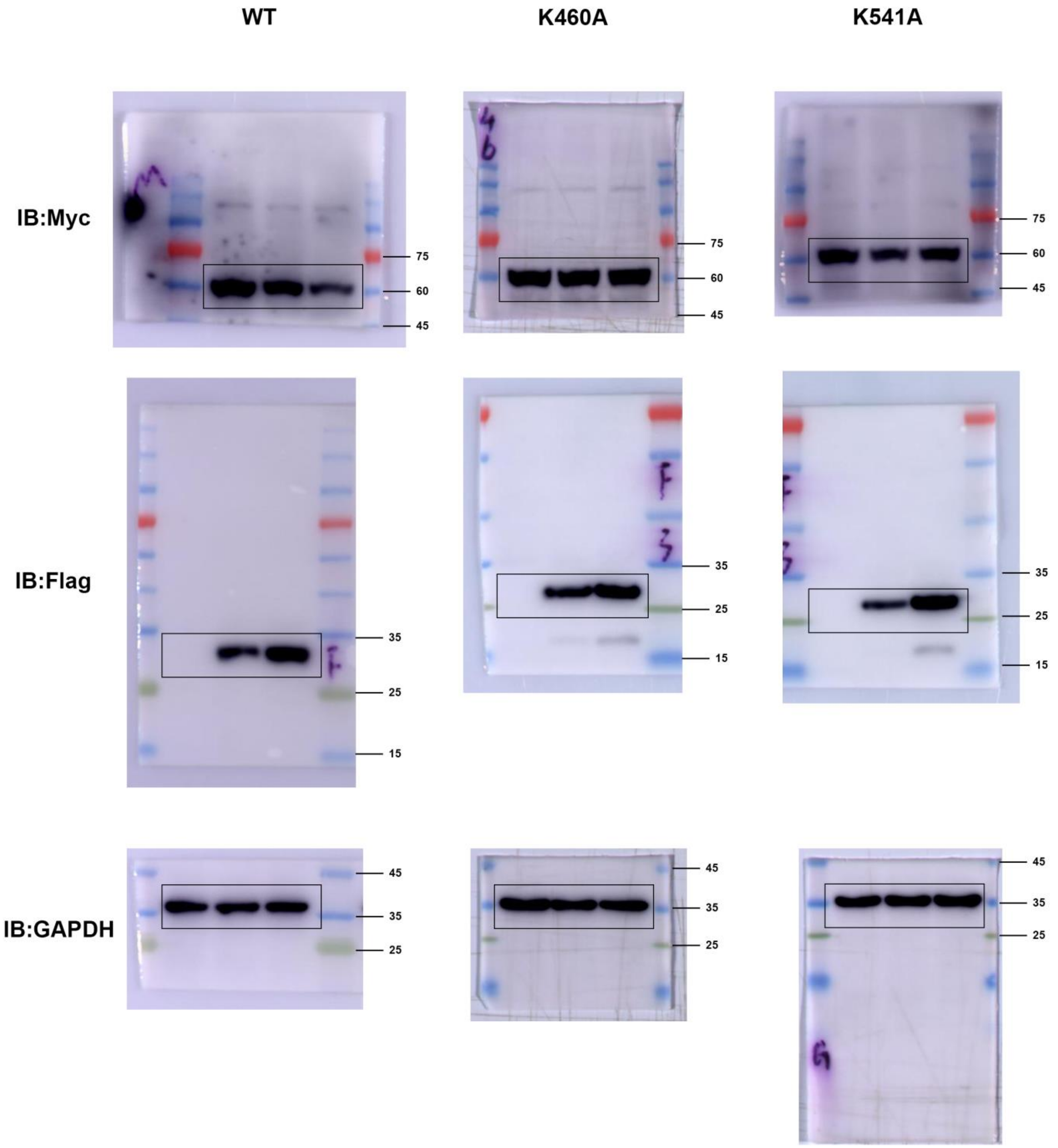

Fig 5G

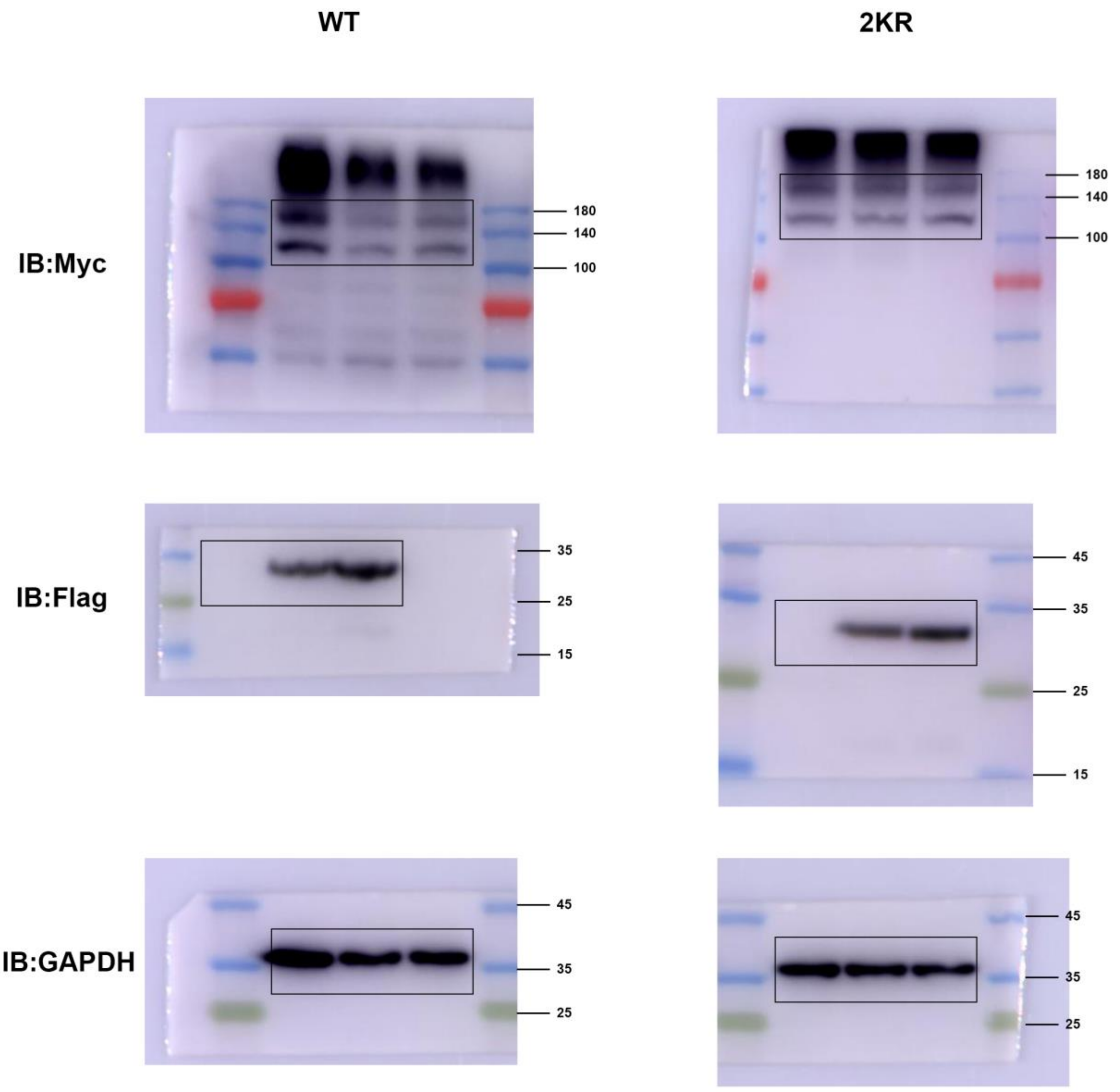
WT
2KR
IB:Myc
180
140
100
180
140
100
IB:Flag
35
25
15
45
35
25
15
IB:GAPDH
45
35
25
45
35
25
