# Supplementary figures and images for: Sigma‐1 receptor attenuates osteoclastogenesis by promoting ER‐associated degradation of SERCA2
Source: EMBO Mol Med. 2022 May 25;14(7):e15373. doi: 10.15252/emmm.202115373 (PMC9260208; doi:10.15252/emmm.202115373)

Fig 7B

IB:NFATc1

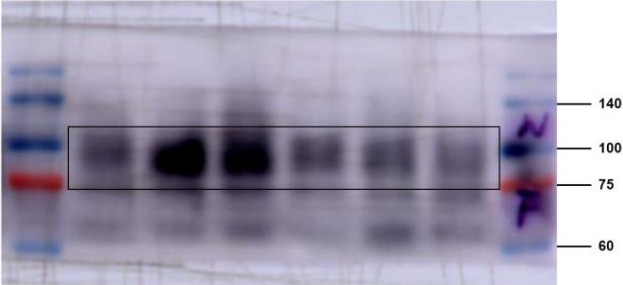

IB:GAPDH

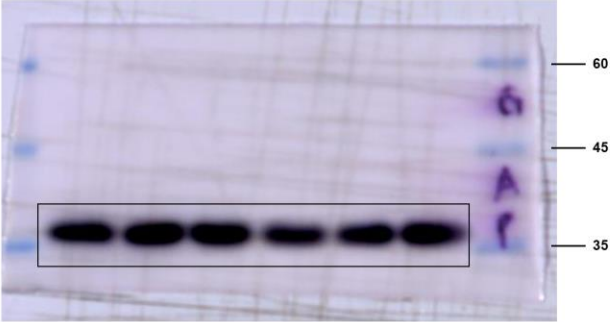

Supplement: Supplementary file 6 — Source Data for Figure 7 [file EMMM-14-e15373-s005.pdf]
