# Supplementary material for: Sigma‐1 receptor attenuates osteoclastogenesis by promoting ER‐associated degradation of SERCA2
Source: EMBO Mol Med. 2022 May 25;14(7):e15373. doi: 10.15252/emmm.202115373 (PMC9260208; doi:10.15252/emmm.202115373)
Supplement: Supplementary file 7 [file EMMM-14-e15373-s003.pdf]

EMBO Molecular Medicine

Sigma-1 receptor attenuates osteoclastogenesis by promoting ER-associated degradation of SERCA2

Xiaoan Wei, Zeyu Zheng, Zhenhua Feng, Lin Zheng, Siyue Tao, Bingjie Zheng, Bao Huang, Xuyang Zhang, Junhui Liu, Yilei Chen, Wentian Zong, Zhi Shan, Shunwu Fan, Jian Chen, and Fengdong Zhao
DOI: 10.15252/emmm.202115373

Corresponding authors: Fengdong Zhao (zhaofengdong@zju.edu.cn) , Jian Chen (chenjian-bio@zju.edu.cn)

Review Timeline:

Submission Date:	3rd Nov 21
Editorial Decision:	25th Nov 21
Revision Received:	6th Apr 22
Editorial Decision:	9th May 22
Revision Received:	10th May 22
Accepted:	11th May 22

Editor: Zeljko Durdevic

Transaction Report:

(Note: With the exception of the correction of typographical or spelling errors that could be a source of ambiguity, letters and reports are not edited. Depending on transfer agreements, referee reports obtained elsewhere may or may not be included in this compilation. Referee reports are anonymous unless the Referee chooses to sign their reports.)

25th Nov 2021

Dear Prof. Zhao,

Thank you for the submission of your manuscript to EMBO Molecular Medicine. We have now received feedback from the three reviewers who agreed to evaluate your manuscript. As you will see from the reports below, the referees acknowledge the interest of the study but also raise important critique that should be addressed in a major revision.

Further consideration of a revision that addresses reviewers' concerns in full will entail a second round of review. EMBO Molecular Medicine encourages a single round of revision only and therefore, acceptance or rejection of the manuscript will depend on the completeness of your responses included in the next, final version of the manuscript. For this reason, and to save you from any frustrations in the end, I would strongly advise against returning an incomplete revision.

We would welcome the submission of a revised version within three months for further consideration. Please let us know if you require longer to complete the revision.

Use this link to login to the manuscript system and submit your revision: Link Not Available

I look forward to receiving your revised manuscript.

Yours sincerely,

Zeljko Durdevic

Zeljko Durdevic
Editor
EMBO Molecular Medicine

***** Reviewer's comments *****

Referee #1 (Remarks for Author):

The authors have not adequately explained why deletion of the Sigma 1 receptor in normal mice does not result in a discernible bone phenotype. In addition, although it is been demonstrated that the Sigma 1 receptor interacts with SERCA 2 and influences it degradation, it is unclear whether SERCA 2 itself alters osteoclast biology.

Referee #2 (Comments on Novelty/Model System for Author):

More repeats of Western Blot data should be shown, otherwise the quality is high.

Referee #2 (Remarks for Author):

In this study the role of Sigma-1 receptor was investigated in bone homeostasis in mice. While in the steady state at the age of 12 month bone parameters seem to be similar at challenging conditions, bone loss was accelerated presumably by increased osteoclast formation. The authors show ex vivo that osteoclastogenesis is enhanced and that agonists in wild type cells decreased cell size. Overexpression of Sigma-1 receptor also slightly reduced OVX mediated trabecular bone loss. Mechanistically they show in cells that Sigmar1 interacts with SERCA2, mediating its degradation and mapped the protein portions responsible. Furthermore they suggest that the Hred1/Sel1L / ERAD pathway is involved. Treatment of disease models with the Sigma-1 receptor agonists Dimemorfan improved bone mass.

Overall this is an intriguing study. However, that the effects of Sigma-1 receptor and the Dimemorfan are entirely mediated by the function in osteoclasts is not entirely clear. Since inhibition of Sigma-1R also increases inflammatory responses, in part the observed effects could be indirect. The authors should find experiments to exclude this possibility or to determine the degree of anti-inflammatory and anti-osteoclastogenic effects in the models they used.

Specific Major Points

The statement that "knockout of Sigmar1 exacerbated bone loss under pathological conditions by promoting osteoclastogenesis" is formally not proven. The authors should try to analyze either a conditional knockout of Sigmar1, in the osteoclasts or at least perform bone marrow transfer experiments of mutant cells into wild type mice and vice versa.

A larger osteoclast does not necessarily means larger efficiency of resorption. Therefore resorption assays have to be performed to proof the point. Intriguingly, this was shown for the drug treatment (Fig. S2E) but not for the Sigmar1 KO cells.

To test the specificity of the drugs used shown in Fig. 3, it would be great to show their effects also in Sigmar1 KO cells.

The overexpression data shown in Fig. S2A by Western blot should be shown for all animals in the experiment to see possible variations. Furthermore, a more specific assessment e.g. in expression in isolated myloid cells would strengthen the case concerning the efficiency of the overexpression in osteoclast progenitor cells. For example primary osteoclasts could be isolated from AAV infected mice and the degree of transgenic overexpression determined. Since the effects were not that strong in Fig. 3C concerning BV/TV, this would be important.

Mechanistically S1R was reported by Rosen et al. 2019 to inhibit IRE1. Is this also evident in osteoclasts? Determination of downstream targets, such as XBP1 splicing could clarify this.

Dimemorfan has strong effects on ameliorating bone loss and decreasing osteoclast activity (Fig. 6). Are there also effects on

osteoblasts and bone formation?

Specific Minor Points:

The authors state no difference in bone parameters in non-challenged mice. The authors could also provide data for vertebrae at least by micro computer tomography.

Similarly also for OVX data from vertebrae would complement the analysis of the phenotype.

Osteocytes are important regulators of bone mass and should be determine in trabecular and cortical bone.

For the OVX the uterus weights should be shown as readout for efficient OVX.

The procedure of the IP-MS is not explained, nor it is clear in which cell types this was carried out.

The decrease of SERCA2 protein in Fig. S3H should be shown in several biological replicates.

In Fig. 5C the increase of ubiquitination by Sigmar1 is not clear and should be clarified.

The effects of Dimemorfan in the CIA model also indicate an immunosuppressive effect that might be independent on osteoclast effects. This should be discussed accordingly.

Referee #3 (Comments on Novelty/Model System for Author):

The authors used several well established mouse models to test their hypothesis. Further, they used human cells for in vitro experiments to show the translational relevance of their findings.

Referee #3 (Remarks for Author):

The authors evaluated the role of the Sigmar1 on bone homeostasis, bone pathology and osteoclast formation in vivo and in vitro. The research question is novel and very clinical< relevant, since Sigmar1 agonists are already in clinical trials. The authors used many different in vivo and in vitro approaches to test their hypothesis. The manuscript is very well written. I would recommend acceptance of this manuscript after some minor revisions.

Specific points to address:

- Fig. 2: I am missing in vivo analysis of number of osteoblasts and osteoclasts in this mouse model. This should be added to complete the dataset and give further evidence about the role of Sigmar1 in vivo

- Fig. 3: Also here, I am missing in vivo cellular analysis (NOc and NOb). Further, it should be stated directly in all figure legends, which statistical test was used. In Fig. 3, I am wondering if all three groups were compared by ANOVA and if so, if there is a difference between sham+vector and OVX+sigmar1 OE

- In general regarding statistics: please state the used test and n-number in the figure legends, in some graphs it is hard to count the n-number. Also, can the authors justify why only n=5 was used in many experiments? This n-number is quite low.

- Discussion: The authors should mention the involvement of Sigmar1 in breast cancer and that Sigmar1 agonists might have a negative effect on breast cancer. This might be important for clinical translation

- Discussion: I don't fully agree with the statement "since bone loss caused by a lack of estrogen is unlikely to be an inflammatory process per se." The authors are right that the direct effects of estrogen on bone cells is very important, but postmenopausal osteoporosis is also in inflammatory process (see PMID: 34024716 or PMID: 34276675). In light of that and based on the anti-inflammatory action of Dimemofan, I would recommend to analyze inflammatory cytokines in the blood of the animals used for Fig. 6. This would also help to explain why basal bone homeostasis is not influenced by Sigmar1, but bone pathology.

Response letter

Dear Zeljko Durdevic and reviewers,

Thank you for your letter and for the reviewers' comments concerning our manuscript. These comments and advice are of great importance and helpful to improve our work. We've studied each comment carefully and made corresponding corrections, we hope these corrections would meet with your satisfaction and approval. All revised portions have been marked red in the latest edition. Please see our point-by-point response to the reviewers' comments and concerns below.

***** Reviewer's comments *****

Referee #1 (Remarks for Author):

We deeply thank you for your constructive comments and suggestions on our manuscript that can help improve our work and guide our future research. Please see our point-to-point response to your issues below.

The authors have not adequately explained why deletion of the Sigma 1 receptor in normal mice does not result in a discernible bone phenotype.

-We speculated that the unchanged bone mass in Sigmar1 gKO versus WT mice under normal conditions was attributed to two perspectives. Firstly, Sigamr1 exerted its molecular chaperone effect whenever ER stress happened, and pathological factors such as ovariectomy resulted in increased stress, which allowed more Sigmar1 activation and promoted Sigmar1 to exert protective function. And Sigmar1 deletion under pathological conditions diminished the protection, leading to severe bone loss. However, under normal or unchallenged conditions, stress was much less than pathological conditions. In line with previous research (Rosen *et al*, 2019), our RT-qPCR experiment showed that no elevation in *xbp1* splicing ratio was found between untreated Sigmar1 gKO and WT BMMs (Fig EV3A), and *xbp1* splicing ratio represented relative IRE1 endonuclease activity and ER stress level. Secondly, our further serological analysis revealed that dimemorfan treatment in OVX and CIA models reduced inflammatory factors (Figs 6M-O and EV5J-L), which indicated that Sigmar1 also showed anti-inflammatory effect in vivo. Since under pathological conditions, activation of osteoclast was coupled with different degree of inflammation. Whereas under steady conditions, there was no excessive osteoclast activity and inflammation. Thus, knockout of Sigmar1 exhibited little or imperceptible effect on bone mass under normal conditions. We have added these explanations accordingly in the Discussion Section.

Figures for reviewers removed

In addition, although it is been demonstrated that the Sigma 1 receptor interacts with SERCA 2 and influences it degradation, it is unclear whether SERCA 2 itself alters osteoclast biology.

--We have introduced SERCA2 functions in regulating osteoclastogenesis in the Introduction Section.

Sacro/endoplasmic reticulum Ca2+-ATPases (SERCAs) are a family of proteins that are involved in calcium homeostasis in multiple cells (Periasamy *et al*, 2017). These proteins are encoded by a multigenic family consisting of SERCA1-3 (*Atp2a1-3*). SERCA2b, encoded by *Atp2a2*, is ubiquitously expressed in smooth muscle and nonmuscle tissues, including neurons (Kim *et al*, 2013). By catalyzing adenosine triphosphate (ATP), SERCA2 is the major calcium transport to reuptake calcium ions from the cytoplasm to the endoplasmic reticulum, and this process is essential for intracellular calcium oscillation

(Dolmetsch *et al*, 1998; Negishi-Koga & Takayanagi, 2009). Knocking down SERCA2 remarkably suppressed calcium oscillation at both frequency and peak values, in contrast, overexpression of SERCA2 notably promoted calcium oscillation(Morita & Kudo, 2010; Zhao *et al*, 2001). Heterozygote SERCA2 (+/-) mice exhibited an osteopetrosis phenotype, and bone marrow-derived macrophages (BMMs) from these heterozygote mice displayed weak calcium oscillations and less osteoclast formation in the presence of receptor activator of nuclear factor-κ B ligand (RANKL) than those from wild-type (WT) littermates (Yang *et al*, 2009). A recent study demonstrated that TMEM64 bound to SERCA2 and regulated SERCA2 activity, and knockout of TMEM64 resulted in reduced SERCA2 activity and decreased osteoclast formation (Kim *et al.*, 2013).

In Fig 4J, SERCA2 was knockdown by siRNA in WT BMMs, and these BMMs showed impaired osteoclastogenesis comparing to those transfected with negative control siRNA. In general, SERCA2 is of great importance for maintaining Ca^{2+} oscillation, which allows NFATc1 translocate into nucleus and promotes osteoclastogenesis.

Figure for reviewers removed

Reference :

Dolmetsch RE, Xu K, Lewis RS (1998) Calcium oscillations increase the efficiency and specificity of gene expression. *Nature* 392: 933-936

Kim H, Kim T, Jeong BC, Cho IT, Han D, Takegahara N, Negishi-Koga T, Takayanagi H, Lee

JH, Sul JY *et al* (2013) Tmem64 modulates calcium signaling during RANKL-mediated osteoclast differentiation. *Cell Metab* 17: 249-260

Morita M, Kudo Y (2010) Growth factors upregulate astrocyte [Ca2+]i oscillation by increasing SERCA2b expression. *Glia* 58: 1988-1995

Negishi-Koga T, Takayanagi H (2009) Ca2+-NFATc1 signaling is an essential axis of osteoclast differentiation. *Immunol Rev* 231: 241-256

Periasamy M, Maurya SK, Sahoo SK, Singh S, Sahoo SK, Reis FCG, Bal NC (2017) Role of SERCA Pump in Muscle Thermogenesis and Metabolism. *Comprehensive Physiology* 7: 879-890

Rosen DA, Seki SM, Fernández-Castañeda A, Beiter RM, Eccles JD, Woodfolk JA, Gaultier A (2019) Modulation of the sigma-1 receptor-IRE1 pathway is beneficial in preclinical models of inflammation and sepsis. *Sci Transl Med* 11

Yang YM, Kim MS, Son A, Hong JH, Kim KH, Seo JT, Lee SI, Shin DM (2009) Alteration of RANKL-induced osteoclastogenesis in primary cultured osteoclasts from SERCA2+/- mice. *J Bone Miner Res* 24: 1763-1769

Zhao XS, Shin DM, Liu LH, Shull GE, Muallem S (2001) Plasticity and adaptation of Ca2+ signaling and Ca2+-dependent exocytosis in SERCA2(+/-) mice. *Embo j* 20: 2680-2689

Referee #2 (Comments on Novelty/Model System for Author):

We deeply thank you for your constructive comments and suggestions on our manuscript that can help improve our work and guide our future research. Please see our point-to-point response to your issues below.

More repeats of Western Blot data should be shown, otherwise the quality is high.
--We have performed each experiment for at least three biological repeats independently, and due to the limitation of the length of the paper, we demonstrated the representative data in the manuscript. In the meantime, we have uploaded all the original Western Blot data. Besides, we have replaced Western Blot data in Fig 5E and Appendix Fig S3A to make sure adequate quality of the figures.

Referee #2 (Remarks for Author):

In this study the role of Sigma-1 receptor was investigated in bone homeostasis in mice. While in the steady state at the age of 12 month bone parameters seem to be similar at challenging conditions, bone loss was accelerated presumably by increased osteoclast formation. The authors show ex vivo that osteoclastogenesis is enhanced and that agonists in wild type cells decreased cell size. Overexpression of Sigma-1 receptor also slightly reduced OVX mediated trabecular bone loss. Mechanistically they show in cells that Sigmar1 interacts with SERCA2, mediating its degradation and mapped the protein portions responsible. Furthermore they suggest that the Hred1/Sel1L / ERAD pathway is involved. Treatment of disease models with the Sigma-1 receptor agonists Dimemorfan improved bone mass.

Overall this is an intriguing study. However, that the effects of Sigma-1 receptor and the Dimemorfan are entirely mediated by the function in osteoclasts is not entirely clear. Since inhibition of Sigma-1R also increases inflammatory responses, in part the observed effects could be indirect. The authors should find experiments to exclude this possibility or to determine the degree of anti-inflammatory and anti-osteoclastogenic effects in the models they used.
--Thank you for your positive comments on our work. We fully agree with your suggestions, we performed ELISA tests for IL-1β, IL-6 and TNF-α in OVX and CIA mouse models (Figs 6M-O and EV5J-L). These inflammatory was elevated after the establishment of OVX or CIA models, dimemorfan reduced these inflammatory factors secretion. These results indicated that activation of Sigmar1 by dimemorfan in pathological models showed anti-osteoclast as well as anti-inflammatory effects. We have determined that Sigamr1 deletion or dimemorfan inhibited osteoclast formation directly in vitro, however, we found it difficult to illustrate two effects separately in vivo, since under pathological conditions, inflammatory factors exhibited pro-osteoclast function, and anti-inflammatory effect of dimemorfan augmented its anti-osteoclast function. Thus, we concluded that Sigmar1 and dimemorfan functioned as anabolic guardians of bone volume, and under pathological conditions, they exhibited anti-inflammatory effect as well. We have discussed this issue

accordingly in the Discussion Section.

Figures for reviewers removed

Specific Major Points

The statement that "knockout of Sigmar1 exacerbated bone loss under pathological conditions by promoting osteoclastogenesis" is formally not proven. The authors should try to analyze either a conditional knockout of Sigmar1, in the osteoclasts or at least perform bone marrow transfer experiments of mutant cells into wild type mice and vice versa.

--Many thanks to your constructive suggestions. Due to the time limits, we find it really difficult to analyze conditional knockout of Sigmar1 mice. Thus, we conducted bone marrow transfer experiments, WT or Sigmar1 gKO bone marrow cells were injected into sublethal irradiated WT mouse. And 6 weeks later, successful transplantation was confirmed by PCR. Ovariectomy surgery was also performed on these mice, all mice were

analyzed 6 weeks after the surgery. As shown in Fig EV2, WT mice transferred with Sigmar1 gKO bone marrow displayed severe osteoporosis phenotype compared to those transferred with WT bone marrow, which is similar to our previous Sigmar1 gKO OVX experiment results. HE staining and TRAP staining also indicated fewer trabeculae and more osteoclasts. These results suggest that knockout of Sigmar1 promotes bone loss under pathological conditions.

Figure for reviewers removed

A larger osteoclast does not necessarily means larger efficiency of resorption. Therefore resorption assays have to be performed to proof the point. Intriguingly, this was shown for the drug treatment (Fig. S2E) but not for the Sigmar1 KO cells.

--We have added resorption assay data for WT versus Sigmar1 gKO BMMs, as shown in Fig 2I and J. Knockout of Sigmar1 increased resorption area percentage compared with WT BMMs, indicating a stronger bone resorbing capacity.

Figure for reviewers removed

To test the specificity of the drugs used shown in Fig. 3, it would be great to show their effects also in Sigmar1 KO cells.

--We performed the relevant experiments, as shown in Appendix Fig S4A-C. In Sigmar1 gKO BMMs, administration of dimemorfan, fluvoxamine and PRE-084 didn't exhibit inhibitory effect comparing to vehicle group, indicating specificity of these drugs in inhibiting osteoclastogenesis by activating Sigmar1.

Figures for reviewers removed

The overexpression data shown in Fig. S2A by Western blot should be shown for all animals in the experiment to see possible variations. Furthermore, a more specific assessment e.g. in expression in isolated myloid cells would strengthen the case concerning the efficiency of the overexpression in osteoclast progenitor cells. For example primary osteoclasts could be isolated from AAV infected mice and the degree of transgenic overexpression determined. Since the effects were not that strong in Fig. 3C concerning BV/TV, this would be important.

--We’ve isolated primary BMMs from AAV infected mice and extracted protein for western

blotting analysis. As shown in Appendix Fig S3A, BMMs infected with Sigmar1-AAV exhibited higher Sigmar1 protein expression comparing to AAV-Vector injected BMMs.

Figures for reviewers removed

Mechanistically S1R was reported by Rosen et al. 2019 to inhibit IRE1. Is this also evident in osteoclasts? Determination of downstream targets, such as XBP1 splicing could clarify this.

--Using RT-qPCR, we determined that, in line with previous research (Rosen *et al*, 2019), under steady conditions, knockout of Sigmar1 didn't affect *xbp*1 splicing. During osteoclast formation, IRE-XBP1 pathway was activated and regulated osteoclastogenesis (Tohmonda *et al*, 2015). Here, we also found that *xbp1* splicing was not altered in the process of osteoclastogenesis (Fig EV3A).

Figure for reviewers removed

Dimemorfan has strong effects on ameliorating bone loss and decreasing osteoclast activity (Fig. 6). Are there also effects on osteoblasts and bone formation?

--By osteogenic differentiation assay, we found that dimemorfan treatment didn't cause significant difference compared with vehicle group (Fig 3J).

Figure for reviewers removed

Specific Minor Points:

The authors state no difference in bone parameters in non-challenged mice. The authors could also provide data for vertebrae at least by micro computer tomography.
--We appreciate your suggestions and we've performed micro-CT analysis on the vertebrae from these non-challenged mice. In accordance with femur data, vertebrae from two genotype show similar trabecular bone mass (Fig 1C and D and Appendix Fig S1C and D).

Figures for reviewers removed

Similarly also for OVX data from vertebrae would complement the analysis of the phenotype.

-We performed micro-CT analysis for vertebrae from Sigmar1 gKO-OVX experiment and the relevant data was shown in Fig EV1B and C.

Figures for reviewers removed

Osteocytes are important regulators of bone mass and should be determine in trabecular and cortical bone.

--Thank you for reminding us with this omit part, we performed immunofluorescence staining on slices with anti-SOST antibody to illustrate osteocyte in situ. As shown in Appendix Fig S1E and F, the counts of those SOST-positive osteocytes per unit area were similar between WT and Sigmar1 gKO in both male and female mice.

Figure for reviewers removed

For the OVX the uterus weights should be shown as readout for efficient OVX.
--We’ve added weights of uterus in revised manuscript (please see Fig EV1A in the revised version).

Figures for reviewers removed

The procedure of the IP-MS is not explained, nor it is clear in which cell types this was carried out.

--The missing part of IP-MS in Materials and Methods has been added in the revised manuscript. Although we performed the IP-MS experiments in HEK-293T cells, we verified the interaction between Sigmar1 and SERCA2 latter in both HEK-293T cells and BMMs.

Immunoprecipitation and mass spectrum analysis

HEK-293T cells were seeded on 6-cm dishes and transfected with vector plasmid or Flag-tagged Sigmar1 plasmid. As mentioned above, the cells were lysed and immunoprecipitated with anti-IgG or anti-Flag antibody after 48 hours. Successful immunoprecipitation of Sigmar1 was verified using silver staining and western blot. The immunoprecipitant protein was then subjected to liquid chromatography with tandem mass spectrometry for proteomics analysis (Novogene, China).

The decrease of SERCA2 protein in Fig. S3H should be shown in several biological replicates.

--We have done several biological replicates and found similar results.

Figure for reviewers removed

In Fig. 5C the increase of ubiquitination by Sigmar1 is not clear and should be clarified.

--We have repeated the experiments and confirmed the increase of ubiquitination of SERCA2 (please see Fig 5E in the revised version).

Figure for reviewers removed

The effects of Dimemorfan in the CIA model also indicate an immunosuppressive effect that might be independent on osteoclast effects. This should be discussed accordingly.

--We performed ELISA tests for IL-1β, IL-6 and TNF-α in the CIA model. As shown in the Fig EV5J-L, the increase of these pro-inflammatory after CIA induction was inhibited by dimemorfan treatment. H&E staining of paws sections indicated that dimemorfan alleviated inflammatory cell infiltration in the joint area, these results suggested that dimemorfan exerted protection in CIA model not only through osteoclastogenesis inhibition but immunosuppressive effect. We have discussed this issue accordingly in the Discussion Section.

Figure for reviewers removed

Reference：

Rosen DA, Seki SM, Fernández-Castañeda A, Beiter RM, Eccles JD, Woodfolk JA, Gaultier A (2019) Modulation of the sigma-1 receptor-IRE1 pathway is beneficial in preclinical models of inflammation and sepsis. *Sci Transl Med* 11

Tohmonda T, Yoda M, Iwawaki T, Matsumoto M, Nakamura M, Mikoshiba K, Toyama Y, Horiuchi K (2015) IRE1α/XBP1-mediated branch of the unfolded protein response regulates

osteoclastogenesis. *J Clin Invest* 125: 3269-3279

Referee #3 (Comments on Novelty/Model System for Author):

We deeply thank you for your constructive comments and suggestions on our manuscript that can help improve our work and guide our future research. Please see our point-to-point response to your issues below.

The authors used several well established mouse models to test their hypothesis. Further, they used human cells for in vitro experiments to show the translational relevance of their findings.

Referee #3 (Remarks for Author):

The authors evaluated the role of the Sigmar1 on bone homeostasis, bone pathology and osteoclast formation in vivo and in vitro. The research question is novel and very clinical< relevant, since Sigmar1 agonists are already in clinical trials. The authors used many different in vivo and in vitro approaches to test their hypothesis. The manuscript is very well written. I would recommend acceptance of this manuscript after some minor revisions.
--We appreciated your positive comments.

Specific points to address:

- Fig. 2: I am missing in vivo analysis of number of osteoblasts and osteoclasts in this mouse model. This should be added to complete the dataset and give further evidence about the role of Sigmar1 in vivo
--We appreciate your suggestion. To show osteoblasts and osteoclasts in vivo, we performed immunohistochemistry staining for osteocalcin (OCN) and TRAP staining. The results were demonstrated in Figs 2C-E and EV 1D-E. Sigmar1 knockout in OVX mice resulted in more osteoclast number, whereas the osteoblast number decreased after OVX surgery and showed no difference between WT and Simgar1 gKO mice.

Figures for reviewers removed

- Fig. 3: Also here, I am missing in vivo cellular analysis (NOc and NOb). Further, it should be stated directly in all figure legends, which statistical test was used. In Fig. 3, I am wondering if all three groups were compared by ANOVA and if so, if there is a difference between sham+vector and OVX+sigmar1 OE

--We've added in vivo cellular analysis for Fig.3 (as shown in Fig 3D-F and Appendix Fig S 3B and C). TRAP and Ocn staining of the femur sections indicated increased osteoclast number and decreased osteoblast number after the OVX, injection of Sigmar1-overexpressing AAV reduced osteoclast number and had little effect on osteoblast number. All statistical tests were filled in each figure legend. In Fig 3, ANOVA is compared with three groups, and there was a significant difference between sham+vector group and OVX+Sigmar1 OE group ($p<0.001$).

Figures for reviewers removed

- In general regarding statistics: please state the used test and n-number in the figure legends, in some graphs it is hard to count the n-number. Also, can the authors justify why only n=5 was used in many experiments? This n-number is quite low.

--Thank you for your constructive suggestions. We've clarified test and n-number we used in every figure legend. As for animal experiments' n-number, we chose n=5 by consulting other researches (Ikebuchi *et al*, 2018; Luo *et al*, 2016; Nishikawa *et al*, 2015) and based on our previous experience in animal experiments (Zheng *et al*, 2021). In our future study design, we would assign more animals in each group (like n=6 or n=8) for a higher reliability.

- Discussion: The authors should mention the involvement of Sigmar1 in breast cancer and that Sigmar1 agonists might have a negative effect on breast cancer. This might be important for clinical translation

--We agreed with your opinion, we have concluded the possibility of side effect by activation of Sigma-1 receptor on breast cancer in the revised Discussion Section (please see in the revised version).

There were some disparate points of view between Sigmar1 and cancer. A few researches suggested that Sigmar1 was highly expressed in breast and lung cancers, and there was a positive correlation between Sigamr1 expression and the cancers' prognosis and invasive ability (Gueguinou *et al*, 2017; Mir *et al*, 2012; Simony-Lafontaine *et al*, 2000; Wang *et al*, 2004). Utilization of Sigmar1 ligands may promote the tumor progression. Thus, in the future clinical usage of Sigmar1 ligands, such as dimemorfan, or further researches, there will be an urgent need to elucidate the possible correlation between these drugs and the breast cancer to rule out any potential risk.

- Discussion: I don't fully agree with the statement "since bone loss caused by a lack of estrogen is unlikely to be an inflammatory process per se." The authors are right that the direct effects of estrogen on bone cells is very important, but postmenopausal osteoporosis is also in inflammatory process (see PMID: 34024716 or PMID: 34276675). In light of that and based on the anti-inflammatory action of Dimemofan, I would recommend to analyze inflammatory cytokines in the blood of the animals used for Fig. 6. This would also help to explain why basal bone homeostasis is not influenced by Sigmar1, but bone pathology.

--We agreed with your constructive suggestions, we performed ELISA test for IL-1β, IL-6 and TNF-α in OVX and CIA models (Figs 6M-O and EV5J-L). These models caused elevated inflammatory cytokines in mice serum, whereas administration of dimemorfan alleviated the effect. Regarding of these results, we speculate that Sigmar1 reduces pro-inflammatory factor production under pathological conditions and lack of Sigmar1 hampers this effect. Whereas in normal mice, osteoclast and inflammatory activities were not that strong, thus Sigmar1 knockout didn't show perceptible effect on bone mass. We have discussed this issue accordingly in the Discussion Section.

Figures for reviewers removed

Reference :

Gueguinou M, Crottès D, Chantôme A, Rapetti-Mauss R, Potier-Cartereau M, Clarysse L, Girault A, Fourbon Y, Jézéquel P, Guérin-Charbonnel C *et al* (2017) The SigmaR1 chaperone drives breast and colorectal cancer cell migration by tuning SK3-dependent Ca(2+) homeostasis. *Oncogene* 36: 3640-3647

Ikebuchi Y, Aoki S, Honma M, Hayashi M, Sugamori Y, Khan M, Kariya Y, Kato G, Tabata Y,

Penninger JM *et al* (2018) Coupling of bone resorption and formation by RANKL reverse signalling. *Nature* 561: 195-200

Luo J, Yang Z, Ma Y, Yue Z, Lin H, Qu G, Huang J, Dai W, Li C, Zheng C *et al* (2016) LGR4 is a receptor for RANKL and negatively regulates osteoclast differentiation and bone resorption. *Nat Med* 22: 539-546

Mir SU, Ahmed IS, Arnold S, Craven RJ (2012) Elevated progesterone receptor membrane component 1/sigma-2 receptor levels in lung tumors and plasma from lung cancer patients. *International journal of cancer* 131: E1-9

Nishikawa K, Iwamoto Y, Kobayashi Y, Katsuoka F, Kawaguchi S, Tsujita T, Nakamura T, Kato S, Yamamoto M, Takayanagi H *et al* (2015) DNA methyltransferase 3a regulates osteoclast differentiation by coupling to an S-adenosylmethionine-producing metabolic pathway. *Nat Med* 21: 281-287

Simony-Lafontaine J, Esslimani M, Bribes E, Gourgou S, Lequeux N, Lavail R, Grenier J, Kramar A, Casellas P (2000) Immunocytochemical assessment of sigma-1 receptor and human sterol isomerase in breast cancer and their relationship with a series of prognostic factors. *British journal of cancer* 82: 1958-1966

Wang B, Rouzier R, Albarracin CT, Sahin A, Wagner P, Yang Y, Smith TL, Meric-Bernstam F, Marcelo Aldaz C, Hortobagyi GN *et al* (2004) Expression of sigma 1 receptor in human breast cancer. *Breast cancer research and treatment* 87: 205-214

Zheng Z, Zhang X, Huang B, Liu J, Wei X, Shan Z, Wu H, Feng Z, Chen Y, Fan S *et al* (2021) Site-1 protease controls osteoclastogenesis by mediating LC3 transcription. *Cell death and differentiation* 28: 2001-2018

9th May 2022

Dear Prof. Zhao,

Thank you for the submission of your revised manuscript to EMBO Molecular Medicine. I am pleased to inform you that we will be able to accept your manuscript pending the following final amendments:

1) In the main manuscript file, please do the following:
- Remove text highlight colour, also in the Appendix.
- Add callouts for EV Figure 1B-E.
- In M&M, provide the antibody dilutions that were used for each antibody.
- In M&M, add statistical paragraph that should reflect all information that you have filled in the Authors Checklist, especially regarding randomization, blinding, replication.
- In M&M, include a statement that informed consent was obtained from all human subjects and that in addition to the WMA Declaration of Helsinki the experiments also conformed to the principles set out in the Department of Health and Human Services Belmont Report.
2) Figures: Please state in the figure legends that Fig EV1D WT Sham image is reused in Appendix Fig S1B.
3) Synopsis: Please check your synopsis text and image and submit their final versions with your revised manuscript. Please be aware that in the proof stage minor corrections only are allowed (e.g., typos).
4) Source data: Please zipp all EV Figures source data and upload it as one file.
5) As part of the EMBO Publications transparent editorial process initiative (see our Editorial at http://embomolmed.embopress.org/content/2/9/329), EMBO Molecular Medicine will publish online a Review Process File (RPF) to accompany accepted manuscripts. This file will be published in conjunction with your paper and will include the anonymous referee reports, your point-by-point response and all pertinent correspondence relating to the manuscript. Let us know whether you agree with the publication of the RPF and as here, if you want to remove or not any figures from it prior to publication. Please note that the Authors checklist will be published at the end of the RPF.
6) Please provide a point-by-point letter INCLUDING my comments as well as the reviewer's reports and your detailed responses (as Word file).

I look forward to reading a new revised version of your manuscript as soon as possible.

Yours sincerely,

Zeljko Durdevic

Zeljko Durdevic
Editor
EMBO Molecular Medicine

***** Reviewer's comments *****

Referee #2 (Remarks for Author):

The authors improved the study a lot and addressed my concerns successfully. Congratulations.

Referee #3 (Comments on Novelty/Model System for Author):

The authors used a variety of well established research models.

Referee #3 (Remarks for Author):

The authors have addressed all of my previous concerns. Especially the inflammatory cytokine data added more relevant information to the manuscript.

The authors performed the requested editorial changes.

2nd Revision - Editorial Decision 11th May 2022

We are pleased to inform you that your manuscript is accepted for publication and is now being sent to our publisher to be included in the next available issue of EMBO Molecular Medicine.

EMBO Press Author Checklist

Corresponding Author Name: Fengdong Zhao, Jian Chen	
Journal Submitted to: EMBO Molecular Medicine	
Manuscript Number: EMM-2021-15373	

USEFUL LINKS FOR COMPLETING THIS FORM
The EMBO Journal - Author Guidelines
EMBO Reports - Author Guidelines
Molecular Systems Biology - Author Guidelines
EMBO Molecular Medicine - Author Guidelines

Reporting Checklist for Life Science Articles (updated January 2022)
This checklist is adapted from Materials Design Analysis Reporting (MDAR) Checklist for Authors. MDAR establishes a minimum set of requirements in transparent reporting in the life sciences (see Statement of Task: 10.31222/osf.io/9sm4x). Please follow the journal's guidelines in preparing your manuscript.
Please note that a copy of this checklist will be published alongside your article.

Abridged guidelines for figures
1. Data
The data shown in figures should satisfy the following conditions:
- the data were obtained and processed according to the field's best practice and are presented to reflect the results of the experiments in an accurate and unbiased manner.
- ideally, figure panels should include only measurements that are directly comparable to each other and obtained with the same assay.
- plots include clearly labeled error bars for independent experiments and sample sizes. Unless justified, error bars should not be shown for technical replicates.
- if n<5, the individual data points from each experiment should be plotted. Any statistical test employed should be justified.
- Source Data should be included to report the data underlying figures according to the guidelines set out in the authorship guidelines on Data Presentation.

2. Captions
Each figure caption should contain the following information, for each panel where they are relevant:
- a specification of the experimental system investigated (eg cell line, species name).
- the assay(s) and method(s) used to carry out the reported observations and measurements.
- an explicit mention of the biological and chemical entity(ies) that are being measured.
- an explicit mention of the biological and chemical entity(ies) that are altered/varied/perturbed in a controlled manner.
- the exact sample size (n) for each experimental group/condition, given as a number, not a range;
- a description of the sample collection allowing the reader to understand whether the samples represent technical or biological replicates (including how many animals, litters, cultures, etc.).
- a statement of how many times the experiment shown was independently replicated in the laboratory.
- definitions of statistical methods and measures:
  - common tests, such as t-test (please specify whether paired vs. unpaired), simple χ2 tests, Wilcoxon and Mann-Whitney tests, can be unambiguously identified by name only, but more complex techniques should be described in the methods section;
  - are tests one-sided or two-sided?
  - are there adjustments for multiple comparisons?
  - exact statistical test results, e.g., P values = x but not P values < x;
  - definition of 'center values' as median or average;
  - definition of error bars as s.d. or s.e.m.

Please complete ALL of the questions below.
Select "Not Applicable" only when the requested information is not relevant for your study.

Materials

Newly Created Materials	Information included in the manuscript?	In which section is the information available? (Reagents and Tools Table, Materials and Methods, Figures, Data Availability Section)
New materials and reagents need to be available; do any restrictions apply?	Not Applicable	

Antibodies	Information included in the manuscript?	In which section is the information available? (Reagents and Tools Table, Materials and Methods, Figures, Data Availability Section)
For **antibodies** provide the following information:<br>- Commercial antibodies: RRID (if possible) or supplier name, catalogue number and or/clone number<br>- Non-commercial: RRID or citation	Yes	Materials and Methods

DNA and RNA sequences	Information included in the manuscript?	In which section is the information available? (Reagents and Tools Table, Materials and Methods, Figures, Data Availability Section)
**Short novel DNA or RNA including primers, probes**: provide the sequences.	Not Applicable	

Cell materials	Information included in the manuscript?	In which section is the information available? (Reagents and Tools Table, Materials and Methods, Figures, Data Availability Section)
**Cell lines:** Provide species information, strain. Provide accession number in repository **OR** supplier name, catalog number, clone number, and/**OR** RRID.	Yes	Materials and Methods
**Primary cultures:** Provide species, strain, sex of origin, genetic modification status.	Yes	Materials and Methods
Report if the cell lines were recently **authenticated** (e.g., by STR profiling) and tested for mycoplasma contamination.	Yes	Materials and Methods

Experimental animals	Information included in the manuscript?	In which section is the information available? (Reagents and Tools Table, Materials and Methods, Figures, Data Availability Section)
**Laboratory animals or Model organisms:** Provide species, strain, sex, age, genetic modification status. Provide accession number in repository **OR** supplier name, catalog number, clone number, **OR** RRID.	Yes	Materials and Methods
**Animal observed in or captured from the field:** Provide species, sex, and age where possible.	Not Applicable	
Please detail **housing and husbandry conditions**.	Yes	Materials and Methods

Plants and microbes	Information included in the manuscript?	In which section is the information available? (Reagents and Tools Table, Materials and Methods, Figures, Data Availability Section)
**Plants:** provide species and strain, ecotype and cultivar where relevant, unique accession number if available, and source (including location for collected wild specimens).	Not Applicable	
**Microbes:** provide species and strain, unique accession number if available, and source.	Not Applicable	

Human research participants	Information included in the manuscript?	In which section is the information available? (Reagents and Tools Table, Materials and Methods, Figures, Data Availability Section)
If collected and within the bounds of privacy constraints report on age, sex and gender or ethnicity for all study participants.	Yes	Materials and Methods

Core facilities	Information included in the manuscript?	In which section is the information available? (Reagents and Tools Table, Materials and Methods, Figures, Data Availability Section)
If your work benefited from core facilities, was their service mentioned in the acknowledgments section?	Not Applicable	

Design

Study protocol	Information included in the manuscript?	In which section is the information available? (Reagents and Tools Table, Materials and Methods, Figures, Data Availability Section)
If study protocol has been **pre-registered, provide DOI in the manuscript**. For clinical trials, provide the trial registration number **OR** cite DOI.	Not Applicable	
Report the **clinical trial registration number** (at ClinicalTrials.gov or equivalent), where applicable.	Not Applicable	

Laboratory protocol	Information included in the manuscript?	In which section is the information available? (Reagents and Tools Table, Materials and Methods, Figures, Data Availability Section)
Provide DOI OR other citation details if **external detailed step-by-step protocols** are available.	Yes	Materials and Methods

Experimental study design and statistics	Information included in the manuscript?	In which section is the information available? (Reagents and Tools Table, Materials and Methods, Figures, Data Availability Section)
Include a statement about **sample size** estimate even if no statistical methods were used.	Yes	Materials and Methods
Were any steps taken to minimize the effects of subjective bias when allocating animals/samples to treatment (e.g. **randomization procedure**)? If yes, have they been described?	Yes	Materials and Methods
Include a statement about **blinding** even if no blinding was done.	Yes	Materials and Methods
Describe **inclusion/exclusion criteria** if samples or animals were excluded from the analysis. Were the criteria pre-established?<br><br>If sample or data points were omitted from analysis, report if this was due to attrition or intentional exclusion and provide justification.	Not Applicable	
For every figure, are **statistical tests** justified as appropriate? Do the data meet the assumptions of the tests (e.g., normal distribution)? Describe any methods used to assess it. Is there an estimate of variation within each group of data? Is the variance similar between the groups that are being statistically compared?	Yes	Materials and Methods

Sample definition and in-laboratory replication	Information included in the manuscript?	In which section is the information available? (Reagents and Tools Table, Materials and Methods, Figures, Data Availability Section)
In the figure legends: state number of times the experiment was **replicated** in laboratory.	Yes	Figures
In the figure legends: define whether data describe **technical or biological replicates**.	Yes	Figures

Ethics

Ethics	Information included in the manuscript?	In which section is the information available? (Reagents and Tools Table, Materials and Methods, Figures, Data Availability Section)
Studies involving **human participants**: State details of **authority granting ethics approval** (IRB or equivalent committee(s), provide reference number for approval.	Yes	Materials and Methods
Studies involving **human participants**: Include a statement confirming that **informed consent** was obtained from all subjects and that the experiments conformed to the principles set out in the WMA Declaration of Helsinki and the Department of Health and Human Services Belmont Report.	Yes	Materials and Methods
Studies involving **human participants:** For publication of **patient photos**, include a statement confirming that consent to publish was obtained.	Not Applicable	
Studies involving experimental **animals**: State details of **authority granting ethics approval** (IRB or equivalent committee(s), provide reference number for approval. Include a statement of compliance with ethical regulations.	Yes	Materials and Methods
Studies involving **specimen and field samples:** State if relevant **permits** obtained, provide details of authority approving study; if none were required, explain why.	Not Applicable	

Dual Use Research of Concern (DURC)	Information included in the manuscript?	In which section is the information available? (Reagents and Tools Table, Materials and Methods, Figures, Data Availability Section)
Could your study fall under dual use research restrictions? Please check biosecurity documents and list of **select agents and toxins** (CDC): https://www.selectagents.gov/sat/list.htm	Not Applicable	
If you used a select agent, is the security level of the lab appropriate and reported in the manuscript?	Not Applicable	
If a study is subject to dual use research of concern regulations, is the name of the **authority granting approval and reference number** for the regulatory approval provided in the manuscript?	Not Applicable	

Reporting

The MDAR framework recommends adoption of discipline-specific guidelines, established and endorsed through community initiatives. Journals have their own policy about requiring specific guidelines and recommendations to complement MDAR.

Adherence to community standards	Information included in the manuscript?	In which section is the information available? (Reagents and Tools Table, Materials and Methods, Figures, Data Availability Section)
State if relevant guidelines or checklists (e.g., **ICMJE, MIBBI, ARRIVE, PRISMA**) have been followed or provided.	Not Applicable	
For **tumor marker prognostic studies,** we recommend that you follow the **REMARK** reporting guidelines (see link list at top right). See author guidelines, under 'Reporting Guidelines'. Please confirm you have followed these guidelines.	Not Applicable	
For **phase II and III randomized controlled trials**, please refer to the **CONSORT** flow diagram (see link list at top right) and submit the CONSORT checklist (see link list at top right) with your submission. See author guidelines, under 'Reporting Guidelines'. Please confirm you have submitted this list.	Not Applicable	

Data Availability

Data availability	Information included in the manuscript?	In which section is the information available? (Reagents and Tools Table, Materials and Methods, Figures, Data Availability Section)
Have **primary datasets** been deposited according to the journal's guidelines (see 'Data Deposition' section) and the respective accession numbers provided in the Data Availability Section?	Not Applicable	
Were **human clinical and genomic datasets** deposited in a public access-controlled repository in accordance to ethical obligations to the patients and to the applicable consent agreement?	Not Applicable	
Are **computational models** that are central and integral to a study available without restrictions in a machine-readable form? Were the relevant accession numbers or links provided?	Not Applicable	
If publicly available data were reused, provide the respective **data citations in the reference list**.	Not Applicable	
