# Supplementary material for: Sigma‐1 receptor attenuates osteoclastogenesis by promoting ER‐associated degradation of SERCA2
Source: EMBO Mol Med. 2022 May 25;14(7):e15373. doi: 10.15252/emmm.202115373 (PMC9260208; doi:10.15252/emmm.202115373)

Fig EV3C

IB:SERCA2

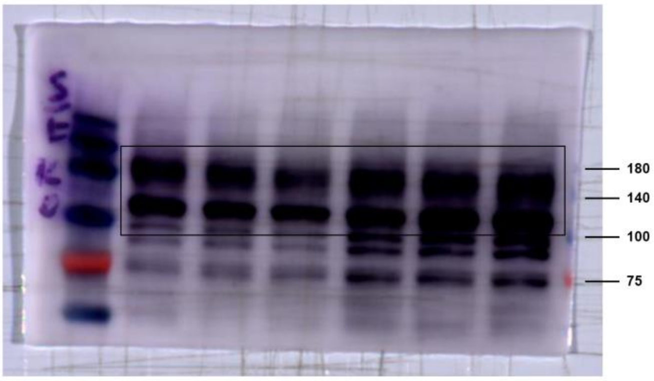

IB:Sigmar1

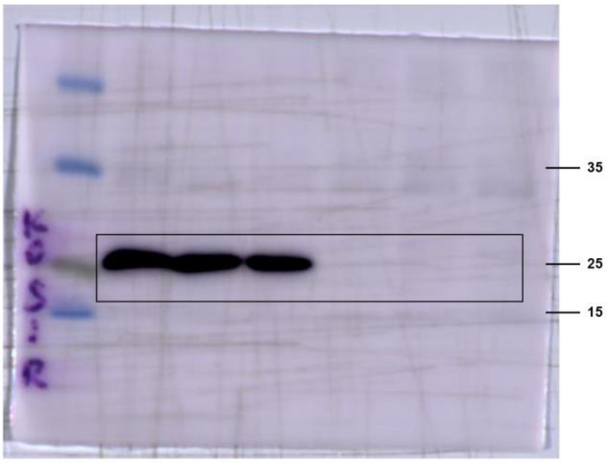

IB:GAPDH

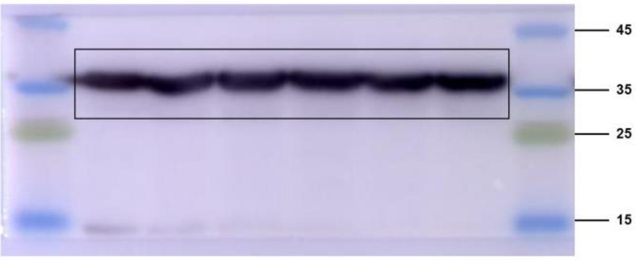

Fig EV3D

IB:Myc

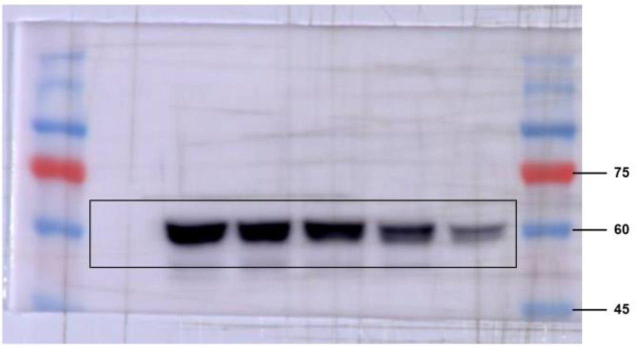

IB:Flag

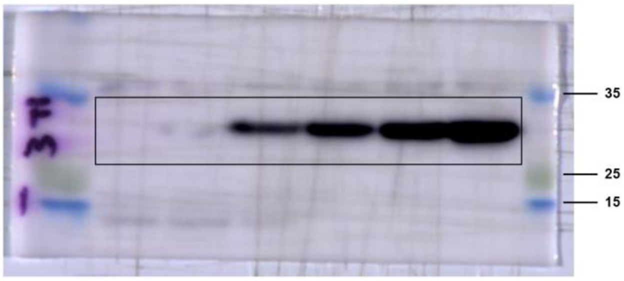

IB:GAPDH

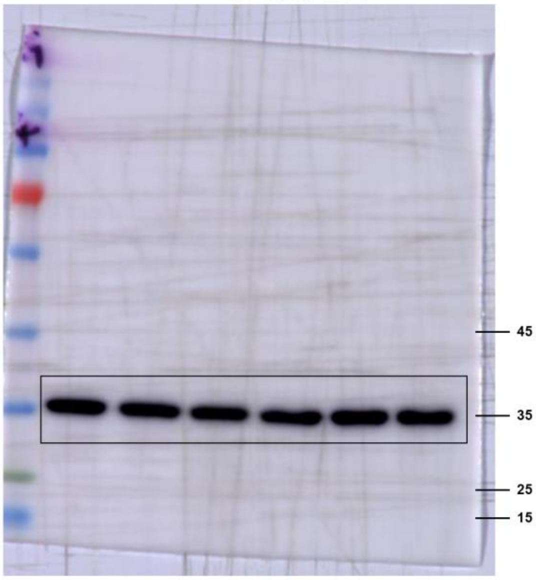

Fig EV3F

IB:Myc

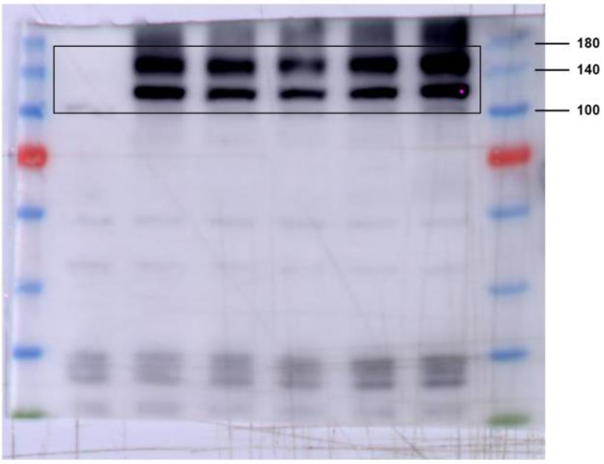

IB:Flag

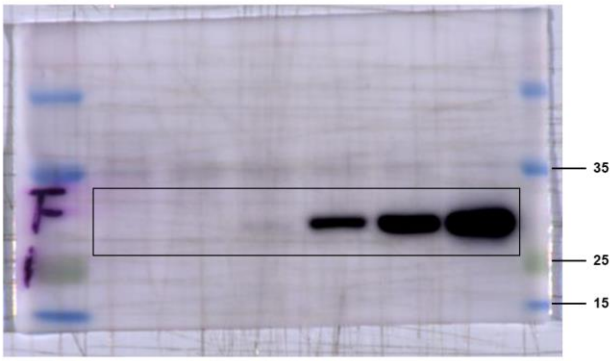

IB:GAPDH

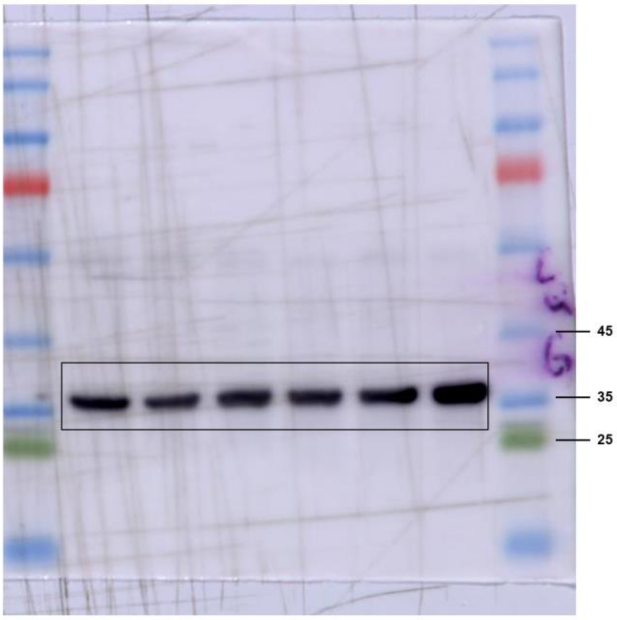

Fig EV3G

IP:Flag
IB:Myc

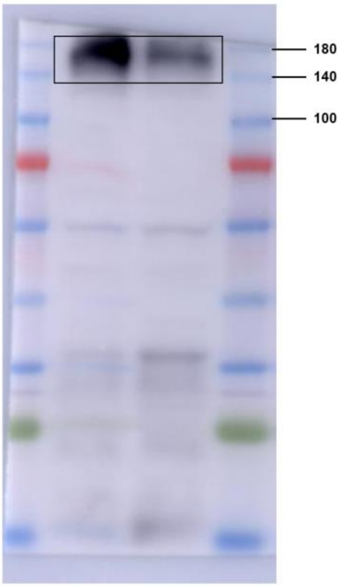

IP:Flag
IB:Flag

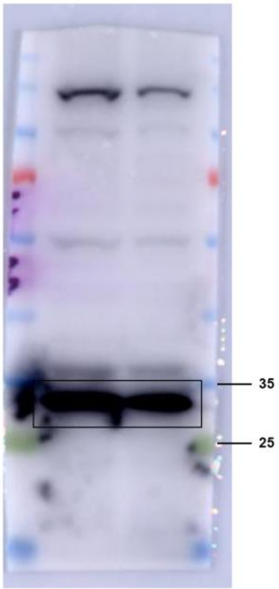

Lysate IB:Myc

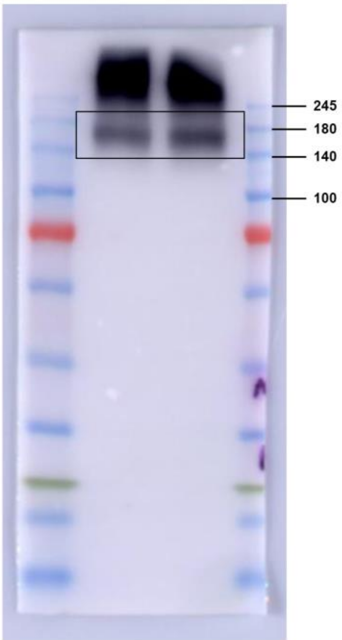

Lysate IB:Flag

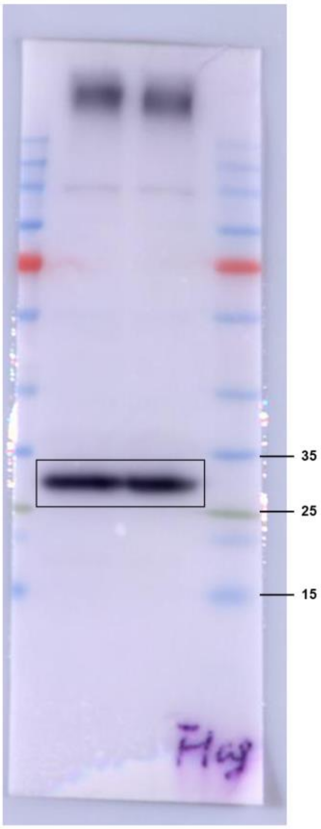

Lysate IB:GAPDH

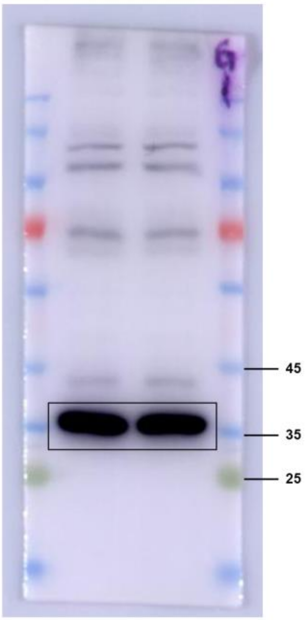

Fig EV3I

IB:SERCA2

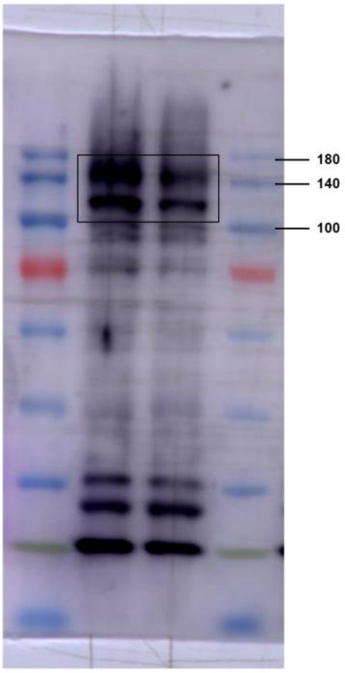

IB:GAPDH

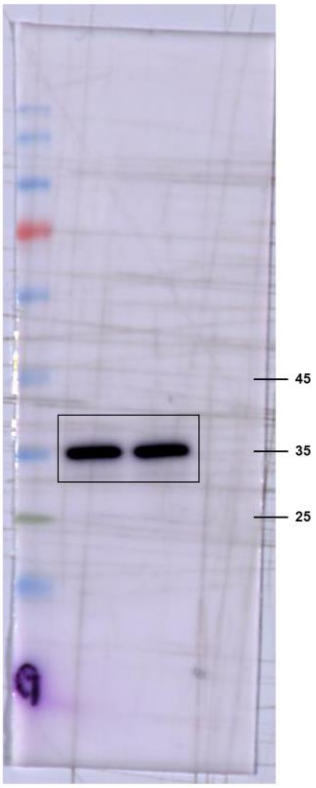

Fig EV3K

IB:SERCA2

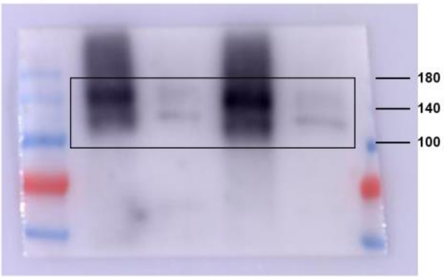

IB:Sigmar1

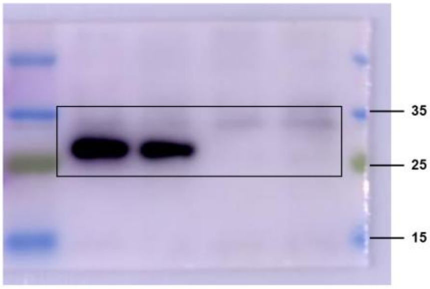

IB:GAPDH

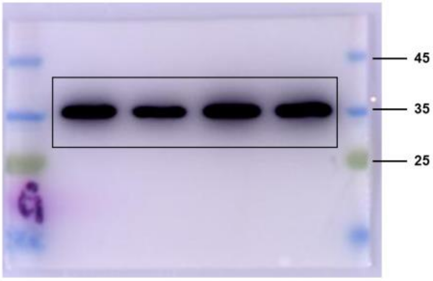

Supplement: Supplementary file 3 — Source Data for Expanded View and Appendix [file EMMM-14-e15373-s007.zip › EMM-2021-15373-V3_SourceDataForExpandedView.zip!/EMM-2021-15373-V3_SourceDataForExpandedView/EMM-2021-15373-V3_Figure_EV3_Source_Data-sd.pdf]
