# Supplementary material for: Sigma‐1 receptor attenuates osteoclastogenesis by promoting ER‐associated degradation of SERCA2
Source: EMBO Mol Med. 2022 May 25;14(7):e15373. doi: 10.15252/emmm.202115373 (PMC9260208; doi:10.15252/emmm.202115373)

Appendix Fig S3A

IB:Sigmar1

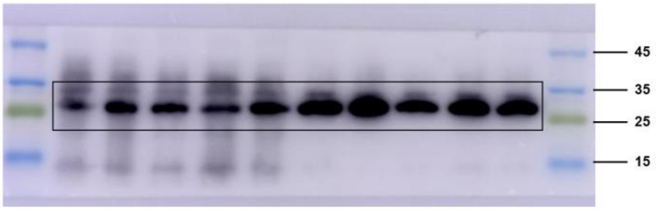

IB:GAPDH

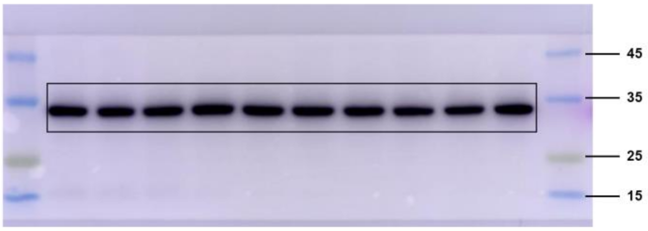

Appendix Fig S4E

IB:c-fos

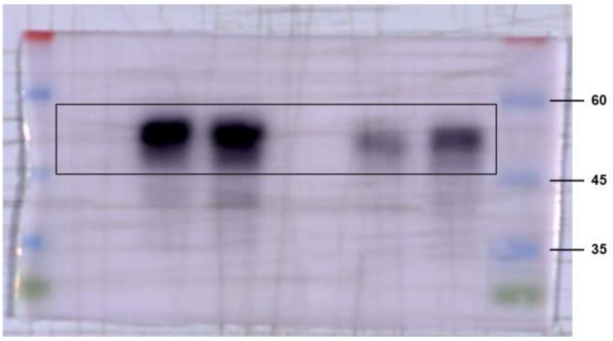

IB:NFATc1

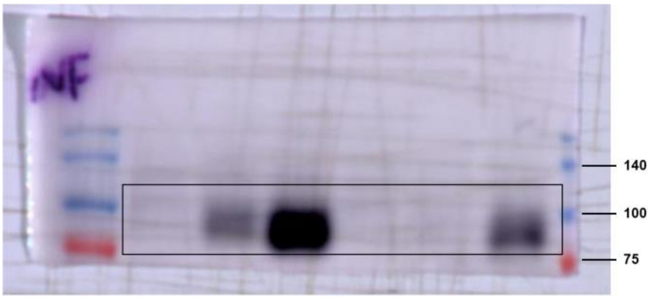

IB:GAPDH

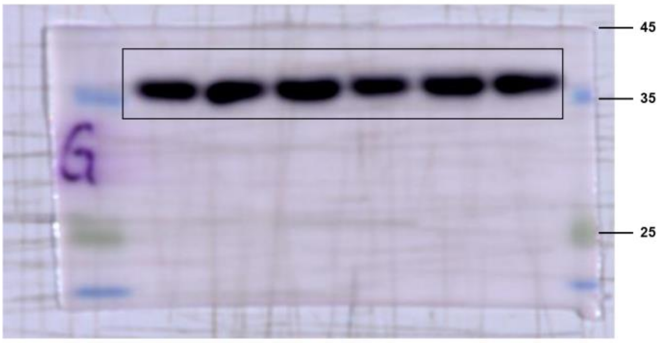

Supplement: Supplementary file 3 — Source Data for Expanded View and Appendix [file EMMM-14-e15373-s007.zip › EMM-2021-15373-V3_AppendixFigureSourceData.zip!/EMM-2021-15373-V3_AppendixFigureSourceData/EMMM-2021-15373_SourceDataForAppendix.pdf]
